# Current status on manufacturing routes to produce metal matrix composites: State-of-the-art

**DOI:** 10.1016/j.heliyon.2023.e13558

**Published:** 2023-02-09

**Authors:** V.K. Parikh, Vivek Patel, D.P. Pandya, Joel Andersson

**Affiliations:** a Department of Mechanical Engineering, School of Engineering and Technology, Navrachana University, Vadodara, Gujarat, India; b Department of Engineering Science, University West, Trollhättan 46186, Sweden

**Keywords:** Manufacturing techniques, Metal matrix composites (MMC), Functionally graded material (FGM), Microstructure, Mechanical properties, Tribological properties

## Abstract

Owing to its excellent properties, Metal Matrix Composites (MMC) has gained popularity and finds application in aerospace, aircraft, shipbuilding, biomedical, biodegradable implant materials and many more. To serve the industrial needs, the manufactured MMC should have homogenous distribution along with minimum agglomeration of reinforcement particles, defect-free microstructure, superior mechanical, tribological and corrosive properties. The techniques implemented to manufacture MMC highly dominate the aforementioned characteristics. According to the physical state of the matrix, the techniques implemented for manufacturing MMC can be classified under two categories i.e. solid state processing and liquid state process. The present article attempts to review the current status of different manufacturing techniques covered under these two categories. The article elaborates on the working principles of state-of-the-art manufacturing techniques, the effect of dominating process parameters and the resulting characteristic of composites. Apart from this, the article does provide data regarding the range of dominating process parameters and resulting mechanical properties of different grades of manufactured MMC. Using this data along with the comparative study, various industries and academicians will be able to select the appropriate techniques for manufacturing MMC.

## Highlights

• Importance of composite materials, manufacturing issues and importance of this review article.
• Comprehensive review of solid state processing techniques involving Friction Stir Processing and Powder Metallurgy.
• Comprehensive review of various casting processes under liquid state processing.
• Effect of dominating process parameters on performance characteristics of composites.
• Comparison of processing techniques, critical analysis and future scope for individual manufacturing routes.

## Graphical abstract

**Figure:**
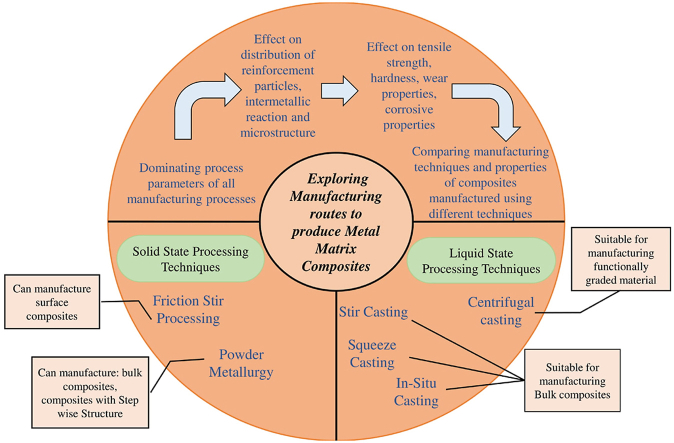

## 1 Introduction

**Fig. 1:**
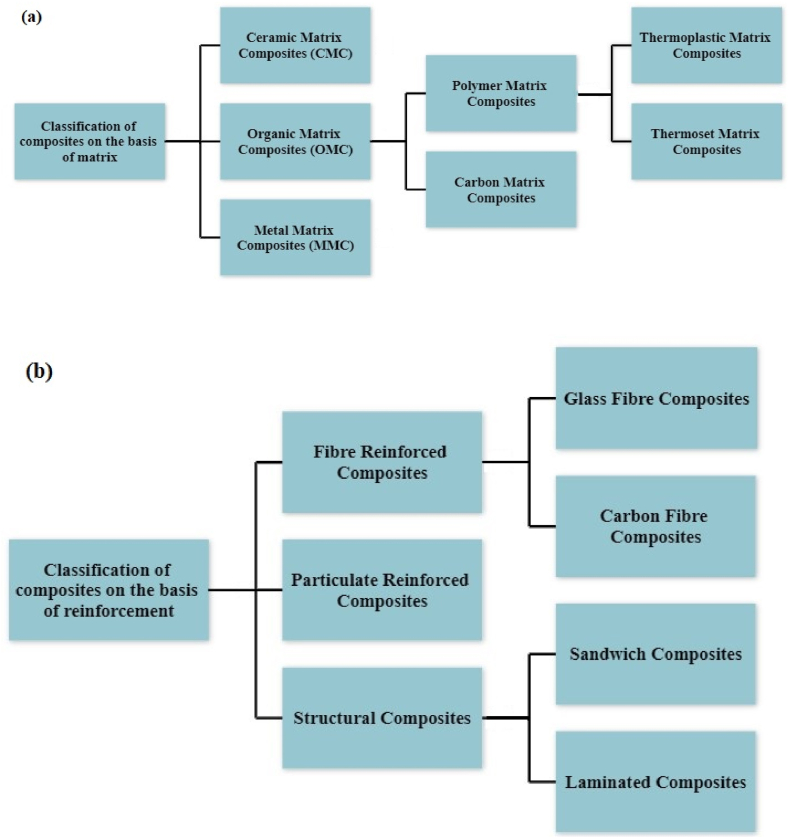
Classification of composite material on the basis of (a) Matrix and (b) Reinforcement.

The development in science and technology results in the evolution of advanced materials which ultimately satisfy industrial needs. Composite material is one such advanced material that is found to have potential application in several industries and is presently replacing the use of conventional material [[1], [2], [3], [4]]. Composite can be defined as materials consisting of a mixture of two or more distinct materials. Materials having distinguished physical and chemical compositions are combined to have excellent properties of resulting composites. Thus, the resulting composites will have the merits of individual materials and will avoid or minimize the demerits of those individual materials. Technically the base alloys are termed as matrix, whereas the secondary phase materials are termed as reinforcement phase. Fig. 1(a) and (b) show the general classification of composite material on the basis of matrix and reinforcement.

Ceramic Matrix Composites (CMC) normally find applications in manufacturing heat-shield for space vehicles, several components of gas turbines such as combustion chamber, turbine blades and stator vanes, components of the burner, flame holder, disk brake, sliding contact bearing and many more [5]. Aerospace industries, automotive industries, marine industries, sports and leisure use Polymer Matrix Composites (PMC) [6]. Metal Matrix Composites (MMC) founds their application in major industries including automobile, aircraft, marine, nuclear, chemical and cryogenics applications [7,8]. Particulate reinforced MMC is the most popular among various derivatives of composites. The particulate of reinforcement present in the matrix can be an organic compound, ceramic particles or some metallic particles [9,10]. In recent articles, it has been reported that among various composites, MMC reinforced with ceramic particles found to have a wide application [[11], [12], [13], [14]]. Particulate reinforced MMC tends to have several excellent properties such as higher specific modulus, superior strength, stable behaviour at elevated temperature, superior tribological properties and lower wear rate as compared to conventional alloys [[15], [16], [17]]. The commonly used particulate for reinforcement are Silicon Carbide (SiC), Aluminium Oxide (Al_2_O_3_), Zirconia (ZrO_2_), Boron Carbide (B_4_C), Titanium Carbide (TiC), Titanium Diboride (TiB_2_) and Aluminum Nitride (AlN) [[18], [19], [20]].

Owing to the research and development in existing or new processing techniques, a consistent increase in demand for MMC or replacement of conventional alloys by MMC can be observed. The characteristic of composites such as distribution of secondary phase particles in the matrix phase, mechanical properties, morphological characteristics, tribological properties and many more depends upon the techniques implemented to manufacture the composite. Thus, considering the potential of MMC it became necessary to review the major manufacturing techniques and their individual features. Existing literature shows that manufacturing techniques can be classified as solid state processing and liquid state processing [[21], [22], [23]]. Also, there exist several ways such as electrophoretic deposition, electrochemical deposition, electroless deposition, spray deposition and so on using which secondary phase particles can be deposited on the surface of matrix materials. However, these are normally being used for manufacturing polymer and epoxy based composites and not MMC. Owing to this, the aforementioned techniques for manufacturing composites will not be discussed in the present article. There exist several review articles related to distinguish manufacturing techniques. These review articles discuss methodology, microstructure characterization, evaluation of mechanical and tribological properties, merits and shortfall of the individual techniques. The subsequent part of this section provides a brief overview of existing review articles.

Padhy et al. [23] reviewed several developed and developing technologies classified under friction stir based technologies. These processes were reviewed on the basis of process parameters, the microstructure of specimens, feasibility and application of various processes. Sharma et al. [24] provided a comprehensive review of surface composites that were manufactured using friction stir processing. The review focused on several aspects related to micro-composites, in-situ composites, nano-composites and hybrid composites. They also emphasized aspects related to tool wear, tool materials and their limitations, cost effective tool material, the mechanism behind the strengthening of surface composite and future trends in the field of friction stir processing. Arifin et al. [25] reviewed the manufacturing of hydroxyapatite and titanium alloy (HA/Ti) composite using powder metallurgy. It was concluded that sintering parameters, especially sintering temperature dominate the phase formation during the diffusion process. Bains et al. [26] reported that apart from the liquid state processing method, all other methods involve the use of expensive experimental setups. Dhandapani et al. [27] reported that compared to other methods, liquid state methods have several advantages such as being simple to use, comparatively cheaper, more flexible and no limitation on shape, size and quantities.

Kandpal et al. [28] identified various dominating process parameters which affect the quality of stir cast composites. They reviewed the effect of process parameters, microstructure and mechanical properties of Aluminium Marix Composite (AMC) manufactured using the stir casting process. It was concluded that parameters such as stirring temperature, stirring speed and stirring time need to be selected with utmost care. Annigeri et al. [29] reviewed the stir casting process and major importance was given to several aspects related to melting condition of base alloy, conditions of secondary phase particles, design of components such as the stirrer, the furnace, the crucible, consequence of process parameters on resulting composites, issues related to casting such as porosity and voids, mould condition, merits and demerits of the stir casting process. Yigezu et al. [30] in their review article specifically address several issues such as the interfacial reaction between matrix and reinforcement particles, casting defects, distribution and agglomeration of secondary phase particles and wettability issues faced during the manufacturing of MMC using the stir casting process. Arunachalam et al. [31] reviewed several aspects related to the design of the furnace, properties of composites, challenges and research opportunities. Naebe and Shirvanimoghaddam [32] successfully reviewed several solid phase, liquid phase, gas based and biopolymeric based processing techniques involved in manufacturing the functionally graded materials. Several processing techniques such as chemical vapour deposition/infiltration, surface reaction process, centrifugal casting, tape casting, slip casting, Gel casting, electrophoretic deposition, laser deposition, spark plasma sintering, powder metallurgy electrospinning and compression moulding were critically analyzed and valuable comments were made regarding manufacturing process and properties of composites.

**Fig. 2:**
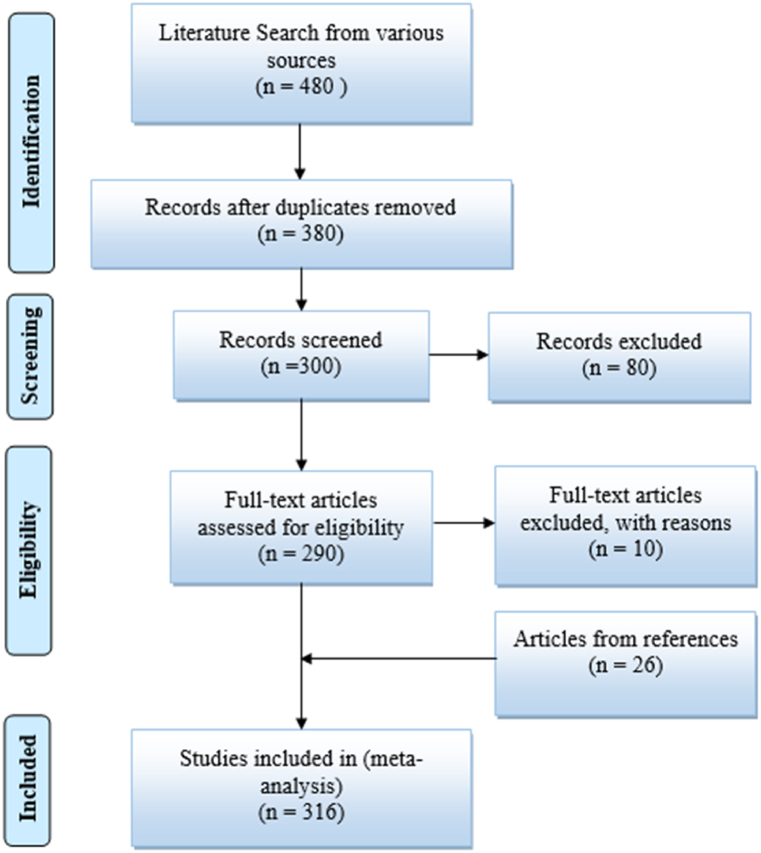
Flow chart of PRISMA method.

From the existing literature, it is clear that the published articles are restricted to reviewing certain specific manufacturing techniques and issues regarding the same. The present article attempts to review commonly adopted both solid state and liquid state techniques used for manufacturing composites. Manufacturing techniques such as Friction Stir Processing, Powder Metallurgy, Centrifugal Casting, Stir Casting, Squeeze Casting and In-situ method has been considered for review. Lastly, the article provides a comparative study which will be helpful for selecting the appropriate technique for manufacturing MMC. It should be noted that the systematic review serves many roles. The systematic review can provide a synthesis of the state of knowledge in the considered field, using which further research priorities can be identified [33]. Even a systematic review can address the questions that might not be answered by individual articles. In the present article, the review of existing literature was carried out by implementing the Preferred Reporting Items for Systematic reviews and Meta-Analysis (PRISMA) method [34]. The flowchart for the PRISMA method is shown in Fig. 2. Various articles from the span of 1981–2022 has been evaluated and reviewed in present review article.

## 2 Solid state processing techniques

To manufacture the surface composites using solid state processing techniques, Friction Stir Processing have been implemented whereas, bulk composites or functionally graded materials can be manufactured using Powder Metallurgy. Manufacturing of bulk composites under solid state processing occurs by blending or mixing the elementary powder in several stages, followed by consolidation. The considered techniques under solid state processing are discussed in the subsequent sections.

### 2.1 Friction stir processing

**Fig. 3:**
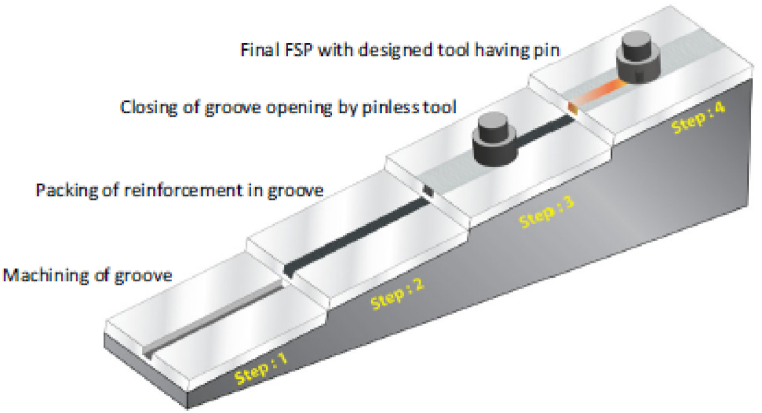
Steps involved in the manufacturing of surface composite using friction stir processing [43].

This solid state surface processing technique follows the principle of Friction Stir Welding [35] and was first reported by Mishra et al. [36]. Friction stir processing has proven to be a capable technique that modifies the microstructure and mechanical properties of surface composites [[37], [38], [39], [40]]. The working of friction stir processing is similar to friction stir welding which involves penetration of a non-consumable rotating tool having a shoulder and pin in the workpiece. Mishra et al. [41] and Ma [42] reported that the traverse motion of the rotating tool and stirring action of the tool will result in the processing of the material. The stirring action results in plastic deformations which will initiate dynamic recrystallization. Initialization of dynamic recrystallization will lead to refinement of microstructure. The different steps involved in the manufacturing of surface composites using friction stir processing are shown in Fig. 3. The first step shown in Fig. 3 indicates the machining of the groove on the surface of the workpiece material. Once the machining of the groove is completed, the groove will be filled or packed with reinforcement materials before performing friction stir processing. In the third step, a pinless rotating tool will be provided with a traverse motion along the groove which will lead to the closing of the groove. Finally, the workpiece material will undergo a second pass with a rotating tool having a pin during which refinement of matrix and reinforcement particles will take place.

However, advancement in the last few decades shows the development of several methods using which the secondary phase can be introduced to the matrix phase during friction stir processing. Some of the commonly implemented methods for introducing secondary phase particles are [44]:

Groove filling method: Groove having predefined dimensions is machined on the surface of the matrix material. Followed by machining, secondary phase particles are introduced into the groove. After filling the groove, friction stir processing is performed on the groove.

Groove filling and closing method: As per the previous method a groove is created and secondary phase particles are introduced into the groove. After filling, the groove is closed by a pinless tool. After closing the groove friction stir processing is performed with a tool having a pin.

**Fig. 4:**
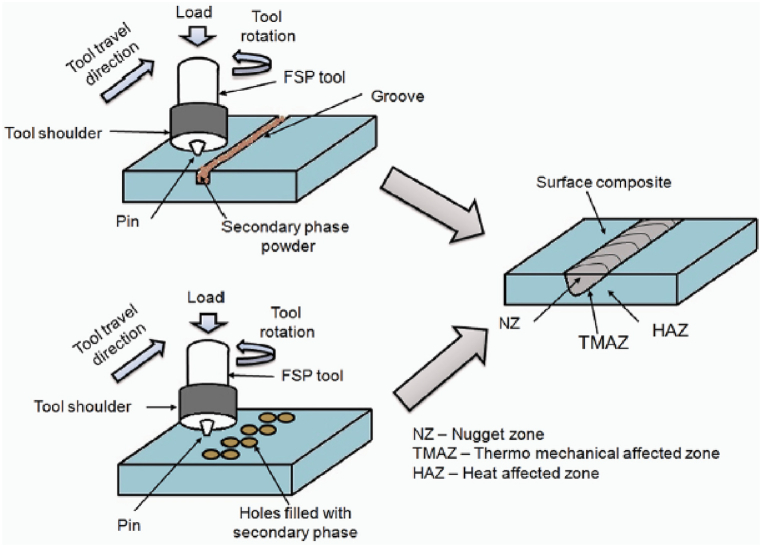
Distinguish method for introducing secondary particle phase in the matrix phase [44].

Hole filling method: Instead of machining groove, little holes are drilled on the matrix surface. Secondary particles are introduced in those holes and then friction stir processing is performed. The same has been represented in Fig. 4.

Hole filling and closing method: Similar to the groove filling and closing method, the hole filled with secondary phase particles is first closed by performing friction stir processing using the pinless tool and the friction stir processing is performed using a tool having a pin.

Sandwich Method: In this method laminates or layers of secondary phase are used. This secondary phase is placed between the matrix material and a sandwich type of arrangement is made. On the performance of friction stir processing, the secondary phase is dispersed throughout the matrix.

It is evidence from the literature that the most common and efficient way used by researcher is either hole filling and closing method or groove filling and closing method [[45], [46], [47], [48]]. Hole filling and closing method deals with the penetration of reinforcement particles whereas, groove filling and closing method provides promising results in adding the reinforcement particles. From the existing literature it has been observed that the microstructure and mechanical properties doesn't affect much irrespective of the technique adopted for adding the reinforcement particles. It should be noted that the groove dimension plays important role in deciding the volume fraction of reinforcement particle incorporated in the matrix. Thus, it becomes necessary to understand the relation between volume of groove and the content of reinforcement particles that will be incorporated in the matrix. Theoretically, by assuming 100% packing of reinforcement particles, the volume fraction of reinforcement particles can be given by equations (1)–(3) [[49], [50], [51], [52]].

(1) $$Volume\;Fraction=\frac{Cross\;Sectional\;Area\;of\;Groove}{Cross\;Sectional\;Area\;of\;Stir\;Zone}\times 100$$

For Rectangular or Square Groove

(2) CrossSectionalAreaofGroove=Width×Depth

For V Groove

(3) $$Cross\;Sectional\;Area\;of\;Groove=\frac{Width\times Depth}{2}$$

In a similar manner, the volume fraction for hole method can be determined by using equations (4)–(6) [[53], [54], [55], [56]]. However, it should be noted that there is established procedure reported for measuring the exact volume fraction of reinforcement particles in the surface composites. The reported equations just provide estimation of the volume fraction which has discrepancy of about 10–30% [[57], [58], [59]]. Apart from this, there exists several other techniques such as point counting, metallographic image analysis and acid dissolution using which volume fraction of reinforcement particles can be measured [[60], [61], [62]]. Furthermore, Sharma et al. [63] in detail has discuss various approaches to measure the volume fraction of reinforcement particles in surface composites.

(4) $$Volume\;Fraction=\frac{Volume\;of\;single\;hole\times total\;number\;of\;holes}{Cross\;Sectional\;Area\;of\;Stir\;Zone\times total\;lenght\;of\;FSP\;Seam}\times 100$$

(5) $$Volume\;of\;Single\;Hole=\frac{\pi }{4}\times {Diameter\;of\;hole}^{2}\times Depth\;of\;the\;hole$$

(6) TotallengthofFSPSeam=PlateLenght−ToolShoulderDiameter

The microstructure of resulting surface composites depends upon certain process parameters such as tool rotational speed, tool traverse speed, axial load, dimension of the tool, tool pin profile, number of passes, size and weight/volume fraction of reinforcement particles and many more. Not only this, but the aforementioned process parameters also alter the mechanical properties, corrosion properties and wear properties of resulting surface composites. Both, tool rotational speed and traverse speed are the sensitive parameters. The increase in rotational speed and decrease in traverse speed tends to generate more heat in processing zone. This will ultimately lead to grain growth and softening of the material [64,65]. On the other hand, reduction in the tool rotational speed and increase in traverse speed results in generation of lack of heat. Hence, careful selection of these two parameter becomes necessary. The tool rotational and traverse speed influences the strength, surface properties, surface quality, and hardness of the surface composites [66,67]. Apart from this, the axial force also influences the heat generated during the processing. Increase in axial load increases the friction between the tool and workpiece and thus increases the heat generation. The axial load generates major effect on the resulting hardness and mechanical strength of the surface composites [[68], [69], [70]]. The tool tilt angle reduces the tool wear along with the reaction force from the friction. Apart from this, providing tool tilt angle allows easy penetration and movement of tool in traverse direction. The tool tilt angle affects the dispersion of the reinforcement particles in the matrix [71,72]. The dimension of tool involves the pin height, pin diameter and the shoulder diameter. These dimension of tool dominates the depth along with the width of the surface composites. More specifically, dimensions of tool pin govern the stirring action required for manufacturing surface composites [[73], [74], [75]]. The profile of tool pin affects the quality of the surface composites by controlling the mixing of reinforcement particles with matrix and distributing the generated heat evenly [[76], [77], [78]]. Lastly, the number of passes takes care about the grain refinement, homogenous distribution of reinforcement particles, breaking the clusters of reinforcement particles, filling the void and other processing defects [[79], [80], [81], [82]].

**Fig. 5:**
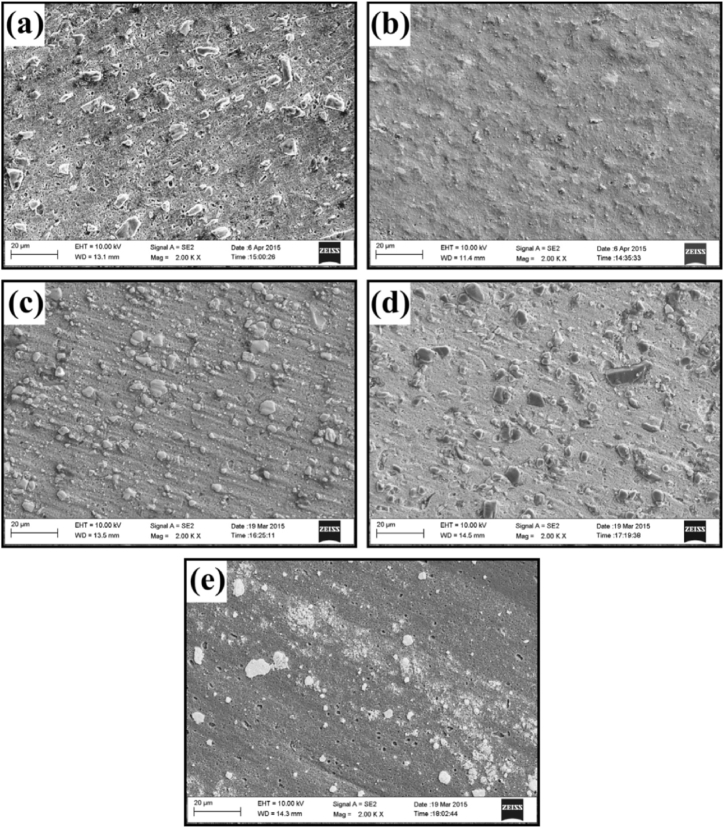
Microstructure of AA 6082 reinforced with (a) SiC (b) Al_2_O_3_ (c) TiC (d) B_4_C (e) WC [83].

From existing literature, it can be observed that commonly used alloys for the matrix phase are aluminium, magnesium and copper, whereas there exist a wide variety of secondary phase particles. Dinaharan [83] investigated the change in microstructure and mechanical properties of surface composites by varying ceramic particles. AA 6082 was used as the matrix phase and different reinforcement particles were SiC, Al_2_O_3_, TiC, B_4_C and WC. On investigating the microstructure and mechanical properties of various surface composites, negligible variation in grain size, tensile strength and hardness were reported. From Fig. 5 (a) – (e), it can be observed that the stirring action of the rotating tool leads to the refinement of secondary phase particles and tends to distribute these particles homogeneously throughout the surface of the matrix. However, both maximum tensile strength and hardness were observed for the AA 6082/TiC composite. It was reported that irrelevant to the type of ceramic particles, the microstructure of all surface composites showed the homogeneous distribution of secondary phase particles. At the same time, no interfacial reaction between the matrix and different reinforcement particles was reported. Due to the same, enhanced mechanical properties along with good interfacial bonding between matrix and reinforcement particles were observed. Also, due to the addition of several types of ceramic particles, fracture mode was found to shift from ductile to brittle.

**Fig. 6:**
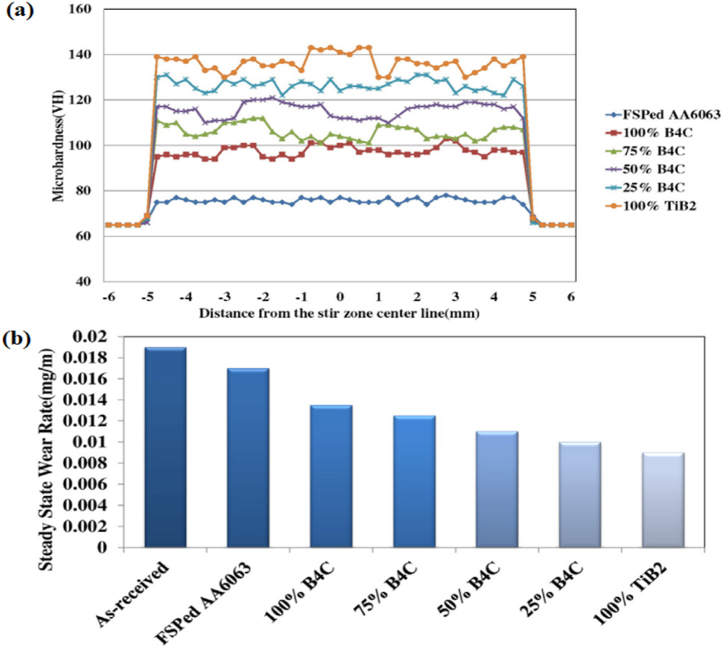
(a) Hardness profile and (b) Wear rate of AA 6063 based surface composite with different weight percentages of reinforcement particles [85].

Kurt [84] manufactured aluminium based composites and investigated variation in tensile strength in the light of process parameters and hybrid ratio. Considered process parameters were rotational speed, traverse speed and volume fraction of different reinforcement particles. Surface composites were prepared by reinforcing AA 5083 with different reinforcement particles such as carbon nanotube (CNT), graphite (Gr), SiC, ZrO_2_ and Al_2_O_3_. Using these reinforcement phases, hybrid composites were manufactured. It was reported that an increase in the volume of CNT, tool rotational speed and traverse speed ultimately improve the tensile strength of surface composites. Among hybrid composites, maximum tensile strength was obtained for AA 5083/10% Gr + 5% ZrO_2_. Results revealed that increasing the tool rotational speed and traverse speed leads to the refinement of secondary phase particles. Owing to this, an improvement in mechanical properties was reported. Narimani et al. [85] manufactured mono and hybrid surface composites of AA 6063 and investigated the consequence of weight fraction of secondary phase particles on microstructure and wear properties. Different types of aluminium based surface composites were manufactured by incorporating milled B_4_C and in-situ TiB_2_. Results of the pin-on-disc sliding wear test showed that with the improvement in hardness, the wear rate of composites was decreased. Superior hardness and wear resistance were observed for the sample consisting of 100% of TiB_2_. The effect of different weight percent of reinforcement particles on the resulting hardness and wear rate is shown in Fig. 6 (a) and (b). Characteristics of ceramic particles are higher hardness and better wear resistance and due to this, the addition of ceramic particles in the matrix results in enhancement of hardness and wear resistance. Also, enhancement in hardness is attributed to two facts: (1) friction stir processing leads to grain refinement which ultimately improves hardness and (2) temperature rise during processing results in annealing of material. Du et al. [86] also observed superior hardness and tensile strength when AA 6061 was reinforced with both Al_2_O_3_ and CNT. Sharma et al. [87] investigate the different reinforcement strategies for manufacturing hybrid composites using friction stir processing. The presence of molybdenum disulfide (MoS_2_) as a solid lubricant develops a mechanically mixed layer that reduces the metal to metal contact on the surface of the wear pin. This indeed enhances the wear resistance of the surface composites. Lastly, it was concluded that hybrid composites manufactured by the hole method revealed 13% lesser wear compared to the specimens manufactured by the groove method. Similarly, Sharma et al. [88] in their another research article manufactured Al 6061 + SiC + graphene and Al 6061 + SiC + carbon nanotube and evaluated the microstructural and mechanical properties of the surface composites. It was reported that the microstructure, microhardness, wear resistance and strength of Al + SiC + graphene was comparatively higher than that of Al 6061 + SiC + carbon nanotubes. The formation of layer by squeezed – out graphene ends to provide the resistance against the wear and applied load. This ultimately enhances the characteristics of the surface composites. Keshavara et al. [89] compared the properties of mono and hybrid composites and reported that hybrid composites tend to have better performance characteristics compared to that of mono composites. Apart from this, the article also discussed about the effect of hybrid composites ratio on resulting performance of the hybrid composites. It was reported that hybrid ratio of graphite/zirconium dioxide = 2 showed highest strength and microhardness with lower elongation. These hybrid composites are now being use by various industries, especially automotive/automobile sector. Various automotive manufacture such as Audi, BMW, Fiat, Ford, Volkswagen uses aluminium alloy based hybrid composites in manufacturing pistons, brake pads, front and rear bumpers, door panel, hood, roof panel, insulation, instrumental panel, boot lining and other such components [90]. Apart from this, owing to its better stiffness, these hybrid composites also find its application in manufacturing of aircraft and aerospace structures. Lastly, these hybrid composites are being widely used in other structural engineering applications such as frames sections, fuselage coating for helicopters and airplanes [[91], [92], [93], [94]].

Thankachan et al. [95] and Thankachan et al. [96] manufactured copper based surface composites reinforced with 5, 10 and 15% boron nitride (BN) and observed a similar trend in the enhancement of several characteristics of surface composites with the increase in weight percent of reinforcement particles. However, it was also reported that an increase in the content of reinforcement particles reduces the ductility and corrosion rate of the surface composites. The corrosion rate reduces with the increase in content of BN particles from 5% to 10%. It was observed that the increase in dispersion of BN particles on the surface of copper tends to move the corrosion potential towards anodic region. This movement confirms the formation of passive layer on the surface the copper which will protect the surface against the corrosion. However, the further increase in content of BN particles leads to improper dispersion and thus increases the corrosion rate for copper reinforced with 15% of BN particles.

**Fig. 7:**
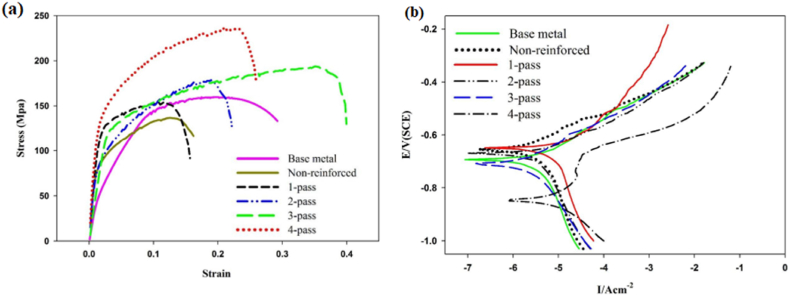
(a) Stress – strain curve and (b) The polarization curve of surface composites processed with different numbers of passes [98].

**Fig. 8:**
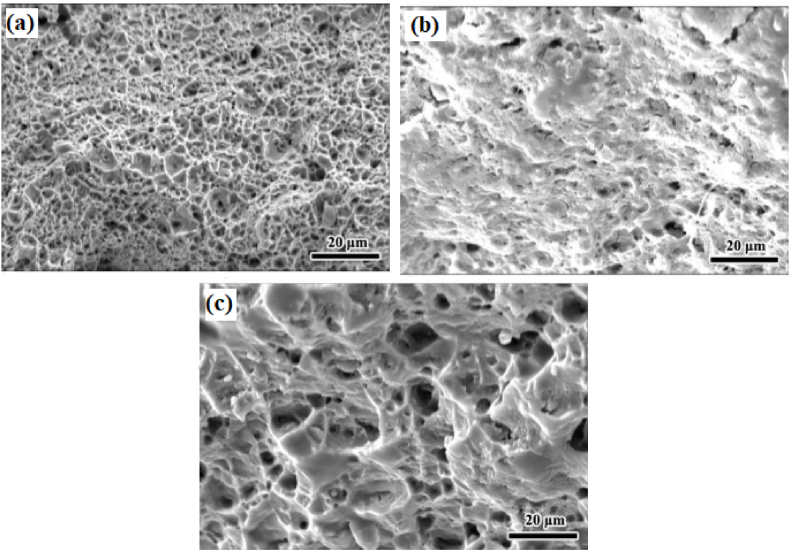
SEM fractrographs of (a) non-reinforced processed, (b) reinforced single pass and (c) reinforced four passes [98].

Dinaharan et al. [97] used fly ash (a waste product from burnt coal) as secondary phase particles and introduced it in AA 6061 to manufacture surface composite. By the addition of fly ash to the aluminium matrix, improvement in hardness, as well as wear resistance was reported. Rathee et al. [43] investigated the variation in microstructure and mechanical properties by changing the ratio of the groove width to the tool shoulder diameter. Five different samples were considered with a different dimension of groove width, constant dimension of shoulder diameter and constant value of optimized process parameters. An increase in groove width from 0 to 3 mm or 40% volume of reinforcement particles consequently improves microhardness. This enhancement in microhardness was observed due to quenching and pinning effects. However, for groove width exceeding 3 mm, a reduction in microhardness was observed. An increase in groove width indicates an increase in the volume of reinforcement particles and this increase in volume tends to reduce the inter-particle spacing. Also, with the increase in groove width, the formation of voids and other defects were observed. Due to the same, resulting composites were characterized by lower microhardness. Thus, 0.5 turnouts to be the optimum ratio of groove width to tool shoulder for obtaining the best characteristics of surface composites. Rahsepar et al. [98] performed multipass friction stir processing on AA 5052/ZrSiO_4_ and investigated the consequence of the same on corrosion behaviour and mechanical properties. It was reported that maximum tensile strength was obtained for specimens processed with four passes. However, maximum corrosion resistance was observed for specimens processed with three passes. The stress – strain curve and the polarization curve with the variation in the number of passes have been represented in Fig. 7(a) and (b). It should be noted that increasing the number of passes increases the depth of penetration of reinforcement particles and the content of particles present on the surface of composites. Apart from this, ZrSiO_4_ particles are cathodic to the aluminium matrix and the reduction in the amount of these particles on the surface will ultimately shift the corrosion potential to a more negative value. Apart from this, scanning electron fractrographs shown in Fig. 8(a)–(c) reveal maximum dimples for four pass processed composites. This indeed indicates that an increase in the number of passes also increases the ductility of the processed composites. Similarly, other researchers also reported higher performance characteristic of the surface composites manufactured using multipass friction stir processing [[99], [100], [101], [102], [103]].

Dadashpour et al. [104] manufactured AZ91C/SiO_2_ surface composite using friction stir processing. It was reported that Hall-Petch and Orowan effects were the dominating mechanisms behind the improvement of tensile strength and hardness. With the increase in the number of passes, a reduction in the aggregation and agglomeration of SiO_2_ particles was observed. Lack of distribution and aggregation of reinforcing particles in the matrix were found to support the cavity formation. These cavities were found to transform into initial cracks which will result in weak bonding. Due to the same, specimen manufactured using a single pass was found to have lower fractural toughness as compared to specimen manufactured using 3 passes. It was observed that the addition of SiO_2_ particles in the magnesium matrix creates stress concentration at the interface of matrix and reinforcement and thus reduces the toughness of resulting composites. Jamshidijam et al. [105] observed improvement in mechanical properties owing to the homogeneously distributed multiwall carbon nanotubes in the magnesium matrix. Compared to magnesium alloys, the grain size was found to reduce to 0.5 μm post processing. This grain reduction and high interfacial strength between reinforcement and matrix enhance the characteristics of the surface composites. The wear resistance of the resulting surface composite was twice the wear resistance of base alloys and this improvement was attributed to a lower coefficient of friction and higher hardness. Bhadouria et al. [106] performed friction stir processing in normal and underwater conditions and investigated the resulting microstructure and tribological properties. Optimum parameters were rotational speed of 1000 rpm, traverse speed of 50 mm/min and 6 numbers of passes. In comparison to specimen processed under normal condition, the underwater processed specimen revealed better microstructural, mechanical and tribological properties. This improvement was accredited to the removal of excess friction heat and dynamic recrystallization which will lead to post grain grown in immersed friction stir processing specimens.

Parikh et al. [13] performed friction stir processing to enhance the microstructure and microhardness of stir cast composites. The stir cast composites were having three compositions i.e. AA 2014 + 5%, 10% and 15% SiC. It was reported that friction stir processed composites were having comparatively higher microhardness and better distribution of reinforcement particles compared to that observed in stir cast composites. The optimum characteristic of composites was observed for AA 2014 + 10% SiC. Bates et al. [107] also implemented friction stir processing as an alternative for enhancing the properties of cast Al–Si alloys. Almost 80% reduction in porosity was reported by performing multiple passes (5 passes) of friction stir processing. The composition of various composites, range of process parameters, tensile strength and hardness of resulting surface composites is presented in Table 1. The data presented in Table 1 may prove helpful for selecting appropriate process parameters for manufacturing specific grades of surface composites.

**Table 1:** Range/magnitude of a process parameter, tensile strength and hardness of different surface composites manufactured using friction stir processing.

Sr. No.	Composite	Process parameter and their range or magnitude	Tensile Strength (MPa)	Hardness	Reference
1	AZ 31 + Multiwall Carbon Nanotubes	Types of Cylindrical Tool: Patterned Shoulder (SC), Patterned Shoulder Stepped Cylindrical (PSSC), Patterned Shoulder Stepped Square (PSSS) Rotational Velocity: 1250, 1600 and 2000 rpm Traverse Speed: 12, 25 and 31.5 mm/min Number of Pass: 1, 2 and 4	–	–	[108]
2	AA 6063 + SiC	Rotational Speed: 1400 rpm Traverse Speed: 56 mm/min Tool Tilt Angle: 2° Shoulder Diameter: 20 mm Pin Diameter: 6 mm Threaded pin length: 2.5 mm	180.7	57 HV	[109]
3	Copper + SiC	Groove Dimension: 1 mm × 1.2 mm Rotational Speed: 900 rpm Traverse Speed: 40 mm/min Tilt Angle: 2° Shoulder Diameter: 20 mm Pin Diameter: 5 mm Pin Length: 2 mm Number of Pass: 1, 2, 4 and 8	215 (8 Pass)	110.6 HV (8 Pass)	[110]
4	AA 2014 + 5% SiC AA 2014 + 10% SiC AA 2014 + 15% SiC	Plate thickness: 6 mm Rotational Speed: 270 rpm Traverse Speed: 78 mm/min Tilt Angle: 2° Shoulder Diameter: 19 mm Pin Length: 5.7 mm Larger Diameter of Pin: 6 mm Smaller Diameter of Pin: 3 mm Number of Pass: 1	–	81.25 HV 88.56 HV 86.97 HV	[111]
5	AA 2014 + 5% SiC AA 2014 + 10% SiC AA 2014 + 15% SiC	Plate thickness: 6 mm Rotational Speed: 190 rpm Traverse Speed: 50 mm/min Tilt Angle: 2° Shoulder Diameter: 19 mm Pin Length: 5.7 mm Larger Diameter of Pin: 6 mm Smaller Diameter of Pin: 3 mm Number of Pass: 1	–	75.36 HV 75.25 HV 81.98 HV	[111]
6	Al 5052 + Al_2_O_3_	Plate Thickness: 4 mm Shoulder Diameter: 13.6 mm Pin Diameter: 5 mm Pin Length: 3.7 mm Groove depth and Width: 2 and 1 mm Range of Rotational Speed to Traverse Speed ratio: 8 to 100 rev/min Range of tilt angle: 2.5° to 5° Number of Pass: 1, 2, 3 and 4	264 (4 Pass)	–	[112]
7	Al 356 + SiC	Plate thickness: 10 mm Shoulder Diameter: 20 mm Pin diameter: 6 mm Pin Length: 3.7 mm Rotational Speed: 1600 rpm Traverse Speed: 50 mm/min Groove Depth and Width: 3.5 and 0.6 mm Tilt Angle: 3°	–	87 HB	[113]
8	Al 356 + MoS_2_	Plate thickness: 10 mm Shoulder Diameter: 20 mm Pin diameter: 6 mm Pin Length: 3.7 mm Rotational Speed: 1600 rpm Traverse Speed: 50 mm/min Groove Depth and Width: 3.5 and 0.6 mm Tilt Angle: 3°	–	81 HB	[113]
9	AZ 91 + 8% SiO_2_	Plate Thickness: 8 mm Rotational Speed: 1200 rpm Traverse Speed: 20, 40 and 63 mm/min Tool having flat shoulder and square pin	–	124 HV	[114]
10	AZ 31 + MWCNTs	Groove Dimension: 1 mm × 2 mm Shoulder Diameter: 12 mm Pin diameter: 4 mm Pin Length: 1.8 mm Rotational Speed: 1500 rpm Traverse Speed: 25 to 100 mm/min Tilt Angle: 3°		78 HV	[115]
11	AZ 91 + SiC	Plate thickness: 5 mm Groove Dimension: 0.8 × 1.2 mm Shoulder Diameter: 15 mm Square Pin Dimension: 3.54 mm Pin Length: 2.5 mm Rotational Speed: 900 rpm Traverse Speed: 63 mm/min Tilt Angle: 3° Number of Pass: 1 and 8	251	139 HV	[116]
12	AZ 91 + Al_2_O_3_	Plate thickness: 5 mm Groove Dimension: 0.8 × 1.2 mm Shoulder Diameter: 15 mm Square Pin Dimension: 3.54 mm Pin Length: 2.5 mm Rotational Speed: 900 rpm Traverse Speed: 63 mm/min Tilt Angle: 3° Number of Pass: 1 and 8	244	134 HV	[116]
13	AZ 31 + Al_2_O_3_	Plate Thickness: 10 mm Groove Dimension: 1.2 mm × 5 mm Shoulder Diameter: 18 mm Pin Diameter: 6 mm Pin Types: Cylindrical without threads, Cylindrical with threads and cylindrical with threads and three flutes Traverse Speed: 45 mm/min Rotational Speed: 800, 1000 and 1200 rpm Tilt Angle: 2° Number of Pass: 2-4	–	92 HV (800 rpm and 4 pass)	[117]
14	AZ 91 + Al_2_O_3_	Plate Thickness: 3 mm Groove Dimension: 0.8 × 2 mm Tool Pin: Square and Triangle Shoulder Diameter: 15 mm Pin Diameter: 5 mm Pin Length: 1.8 mm Rotational Speed: 900 rpm Traverse Speed: 63 mm/min Tilt Angle: 3° Number of Pass: 1 and 3	–	103.2 HV (Square Pin)	[118]
15	AA 6061 + Al_2_O_3_	Plate Thickness: 5 mm Hole Depth: 3 mm Hole Diameter: 2 mm Tool Tilt angle: 3° Shoulder Diameter: 18 mm Pin Diameter: 4 mm Pin Length: 4 mm Rotational Speed: 1000 rpm Traverse Speed: 25 mm/min Number of Pass: 1 and 2	–	95.75 HV (single Pass) 94.90 HV (double Pass)	[119]
16	AA 7075 + Al_2_O_3_ (nano-particles)	Plate Thickness: 15 mm Groove dimension: 7.5 mm depth and 1 mm width Shoulder Diameter: 20 mm Probe Diameter: 6 mm Probe Length: 10 mm Rotational Speed: 840 rpm Traverse Speed: 40 mm/min Tilt Angle: 3° Number of Passes: 1, 3 and 4	–	132 HV (4 passes)	[120]
17	AA 6061 + 100% B_4_C AA 6061 + 75% B_4_C + 25% MoS_2_ AA 6061 + 50% B_4_C + 50% MoS_2_	Plate thickness: 6 mm Rotational Speed: 545 rpm Traverse Speed: 50 mm/min Tool Tilt Angle: 3° Hole Diameter: 1.5 mm Hole Depth: 2 mm Number of Passes: 3	–	112 HV 106 HV 102 HV	[121]
18	AA 6061 + SiC + Graphite	Groove Dimension: 2 × 3 mm Plate thickness: 6 mm Rotational Speed: 1800 rpm, 2200 rpm and 2500 rpm Traverse Speed: 25 mm/min Shoulder Plunge Depth: 0.2, 0.3 and 0.4 mm	–	0.35 GPa	[122]
19	AA 6082 + SiC	Plate Dimension: 150 × 75 × 6 mm Rotational Speed: 140 rpm Traverse Speed: 40 mm/min EN – 31 steel tool with square pin Shoulder Diameter: 21 mm Pin Length: 4.5 mm	259 MPa (3 passes)	154 HV (3 passes)	[123]
20	Copper + SiC	Plate Dimension: 200 × 100 × 6 mm Rotational Speed: 1000 rpm Traverse Speed: 30 mm/min Tool tilt angle: 2° Shoulder Diameter: 18 mm Pin Length: 4.5 mm Pin Diameter: 6 mm	185 MPa	128 HV	[124]

Thus, there exists a variety of literature whose major concern was to obtain desire mechanical properties i.e. tensile strength and hardness, better resistance towards wear and corrosion, homogenous distribution of secondary phase particles and attaining super – plasticity in manufactured surface composites and to optimize process parameters [[125], [126], [127], [128], [129]]. It should also be noted that friction stir processing has been implemented for modification of microstructure and thus enhancing the mechanical properties of the bulk composites [102,103,130,131]. However, it should be noted that friction stir processing fails to manufacture bulk composites or functionally graded materials. Thus to manufacture this, powder metallurgy can be implemented.

### 2.2 Powder metallurgy

Powder metallurgy is one more solid state technique using which bulk composites or functionally grade materials can be manufactured at low cost. The powder metallurgical is capable to manufacture complex geometry of various metals and alloys. Sandwich structures composed of a porous metallic foam core and metallic face sheets can also be produced along with higher mechanical properties. This closed loop of microstructure has an attractive application in industry where weight reduction and energy absorption are major issues [132]. Powder metallurgy is widely used to manufacture high strength Ti based alloys, Carbon nanotube with aluminium, nickel, copper, magnesium, and other metal matrix which has a wide application in aerospace industry [133]. This technique is divided into major four steps. The first step involves breaking primary material and converting it into powder form. In the second step, the primary material in the powder form is mixed or blended with powder of another metal or non-metal. This homogenously blended mixture is then introduced to a mould cavity or dies where it undergoes compression. This compression will ultimately result in the formation of a weak cohesive mass known as green compact [134]. At last, this green compact undergoes a sintering process during which it is subjected to high temperature and pressure for a pre-defined time. Nemat-Alla et al. [135] reported that the sintering process enhances the hardness of green compact. Chaira [136] has presented several derivatives of the powder metallurgy route implemented for the manufacturing of composite materials. The article discusses several conventional and state – of – the – art techniques covered under powder metallurgy.

**Fig. 9:**
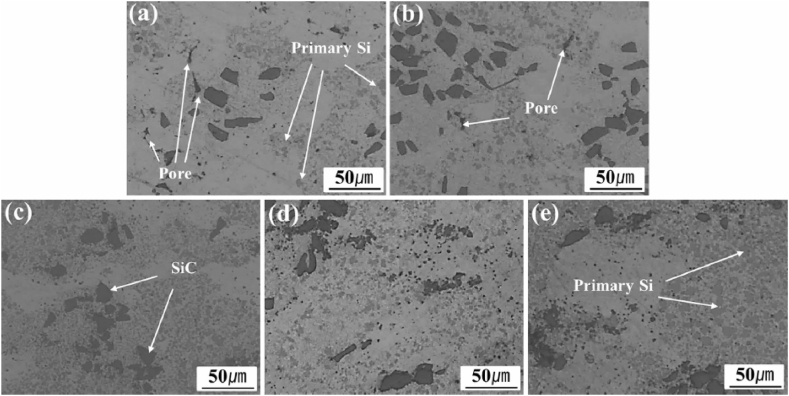
Microstructure of sintered samples manufactured at various sintering temperatures (a) 480 °C (b) 520 °C (c) 560 °C (d) 580 °C (e) 600 °C [139].

It should be noted that powder metallurgy results in the formation of a stepwise structure. Yankee et al. [137] used powder metallurgy and liquid phase sintering for the manufacturing iron based and aluminium based composites. These composites were reinforced with various reinforcement materials. Composite manufactured using this technique were Fe–C–Si and Fe–Cu, reinforced with ZrO_2._ Additionally, composites based on Al–Cu–Si–Mg alloy and reinforced with SiC or Al_2_O_3_ were also manufactured. Similarly, Ren et al. [138] used powder metallurgy to manufacture AZ31 reinforced with 5%, 10%, 15% and 20% of ZrB_2_. It was reported that homogeneously distributed secondary particles tend to improve the hardness and wear resistance. Bang et al. [139] examined the microstructure and mechanical properties of aluminium based composites. They studied the consequence of sintering temperature and sintering time on resulting microstructure and mechanical properties. The microstructure of composite at various sintered temperatures is shown in Fig. 9 (a) – (e). Due to the fine distribution of the Q-phase and θ-phase of Alumix 231 during sintering, improvement in ultimate tensile strength and elongation was reported. Orowan strengthening mechanism was also responsible for these enhanced properties. The Orowan strengthening mechanism indicates dislocation and distribution of hard particles. Enhancement in mechanical properties was also attributed to the densification and distribution of SiC particles. Among various sintering conditions, optimum results were obtained for a sintering temperature of 580 °C and a sintering time of 1 h.

Jeevan et al. [140] manufactured AA 6082 + x% ZTA (x = 3, 6 and 9) composite and evaluated variation in density. The green compact was prepared at a pressure of 500 MPa, sintering temperature of 620 °C and sintering time of 1 h. It was reported that the addition of reinforcement particles had a slightly negative effect on densification during the sintering process. Verma et al. [141] manufactured Al_2_O_3_, Al_2_O_3_ + 40% ZrO_2_ and Al_2_O_3_ + 30% ZrO_2_ + 10% CeO_2_ composites and evaluated the effect of sintering temperature. By maintaining constant pressure and sintering time of 100 MPa and 2 h respectively, sintering was performed at three different temperatures of 1600 °C, 1650 °C and 1700 °C. Among these composites, Al_2_O_3_ + 40% ZrO_2_ composite manufactured at a sintering temperature of 1700 °C was found to have the highest hardness and fracture toughness. Also, ZrO_2_ particles were found to restrict abnormal growth of alumina and thereby resulting in the formation of high bonding between them. Shabani et al. [142] while manufacturing copper based MMC, reported that composites manufactured using a sinter – forged process tend to have higher hardness and density compared to the specimens manufactured by the conventional sintering process. Similar results were also reported by Necina and Pabst [143] for sinter forged MgAl_2_O_3_ composites. Also, a lower sintering temperature was required under compressive stress during the sintered forged process.

Yang et al. [144] investigated the effect of two different sintering additives i.e. Y_2_O_3_ and CeO_2_ on the resulting microstructure and mechanical properties of Si_3_N_4_/SiC ceramics. It was reported that the addition of these sintering additives tends to reduce the density and flexural strength of Si_3_N_4_/SiC ceramics. The reason for this reduction was an increase in porosity in the resulting composites. On performing the chemical composition, the main composition of the sample was β-Si_3_N_4_ and SiC. Apart from this, the liquid phase of Y–Si–O–N and Ce–Si–O–N was also reported in the composition of composites. On the other hand, Oguntuyi et al. [145] used SiC as a sintering additive and investigated the resulting microstructure, hardness, wear resistance and densification of TiB_2_ ceramics. Two different composites i.e. TiB_2_ + 10 wt% SiC and TiB_2_ + 20 wt% SiC were manufactured using the spark plasma sintering method. These composites were manufactured at a sintering temperature of 1850 °C for 10 min under the pressure of 50 MPa. Among both manufactured compositions, TiB_2_ + 20 wt% SiC revealed better characteristics. During characterization, diverse in-situ phase and microstructure alteration was reported. The in-situ phase of TiC was found to serve as the contributing parameter for the enhancement of the characteristics of the resulting sintered composites.

Klein and Binder [146] investigated the effect of the addition of phosphorous quantity in the matrix of a nickel alloy. Two different quantities of phosphorous i.e. 0.3 wt% and 0.75 wt% was added to nickel alloy. Along with this, 10 vol% of h-BN was also added which normally acts as a solid lubricant. Using the plasma sintering process composites were manufactured at a sintering temperature of 1150 °C for a time period of 60 min under the pressure of 600 MPa. The average hardness of composition with 0.3 wt% and 0.75 wt% of the phosphorous was 236 HV and 326 HV respectively. The microstructure of nickel alloy with 0.3 wt% of the phosphorous revealed the presence of solid lubricant particles in the original size of 10–20 μm. Along with this, the microstructure was found to have low porosity. On the other side, the microstructure of nickel alloy reinforced with 0.75 wt% of the phosphorous revealed presence of lubricant having a size of 50–100 μm. The formation of these lubricant particles was a result of the liquid phase which was generated due to sintering. Also, the presence of a liquid phase during sintering supports the rearrangement of the solid lubricant phase and tends to distribute the lubricant particle more homogeneously. Zhang et al. [147] manufactured the Al_2_O_3_-SiCw-Si_3_N_4_ composite using spark plasma sintered process. Effect of sintering temperature on mechanical properties and microstructure were investigated. Enhancement in mechanical properties is observed at 1500 °C temperature at low thermal budget. Kitiwan et al. [148] also successfully manufactured SiO_2_-diamond composites with 75 mass% diamond by spark plasma sintering. Singh et al. [149] reported that microwave sintering is widely used to manufacture metal matrix composites and alloys at low a production costs, processing times along with enhancement in product properties. Similar analysis is also reported by Mishra et al. [150]. Wei et al. [151] manufactured four-layered diamond/W–Cu based functionally graded material by microwave sintering. The good interface was observed between the two layers along with improvement in the thermal conductivity of the composite.

**Fig. 10:**
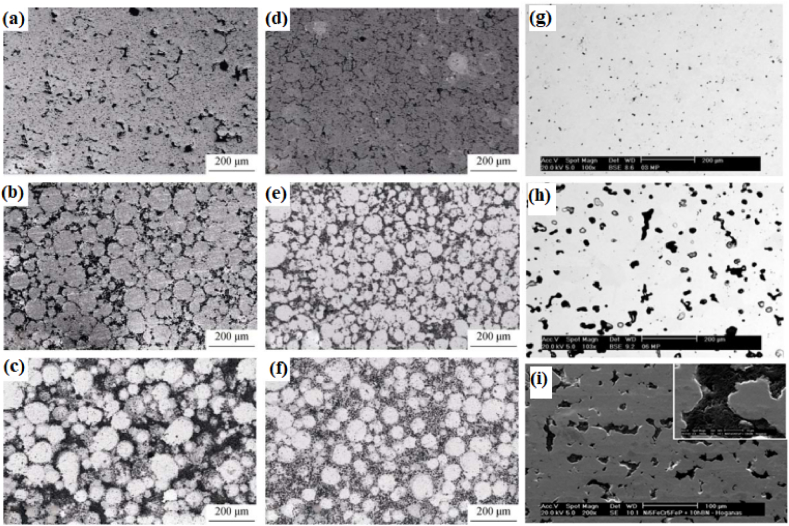
Microstructure of (a) sintered Cu, (b) Sintered Cu + 20 vol% SiC, (c) Sintered Cu + 40 vol% SiC, (d) sinter - forged Cu, (e) sinter - forged Cu + 20 vol% SiC, (f) Sinter - forged Cu + 40 vol% SiC [57], (g) Ni–Fe – Cr–P + 0.3% phosphorous, (h) Ni–Fe – Cr–P + 0.75% phosphorous (i) Ni–Fe – Cr–P + 0.75% phosphorous + 10% h-BN [142,146].

Ashwath and Xavior [152] investigate the effect of different weight percent of SiC and Al_2_O_3_ in the aluminium matrix. The weight percent of reinforcement particles were 3, 6 and 9. The composites were subjected to microwave sintering and it was observed that the hardness and compressive strength of sintered composites were found to increase with the increase in weight percent of reinforcement particles. However, good ductility, formability and stress-strain behaviour were obtained for composites reinforced with 6% of SiC and Al_2_O_3_. The microstructure of various composites manufactured by sintering + powder metallurgy or sinter – forged + powder metallurgy is shown in Fig. 10 (a)–(i). From Fig. 10 (a) – (i), it can be said that irrespective of the matrix or reinforcement phase, the composites manufactured using this technique leads to the homogenous distribution of reinforcement particles.

**Fig. 11:**
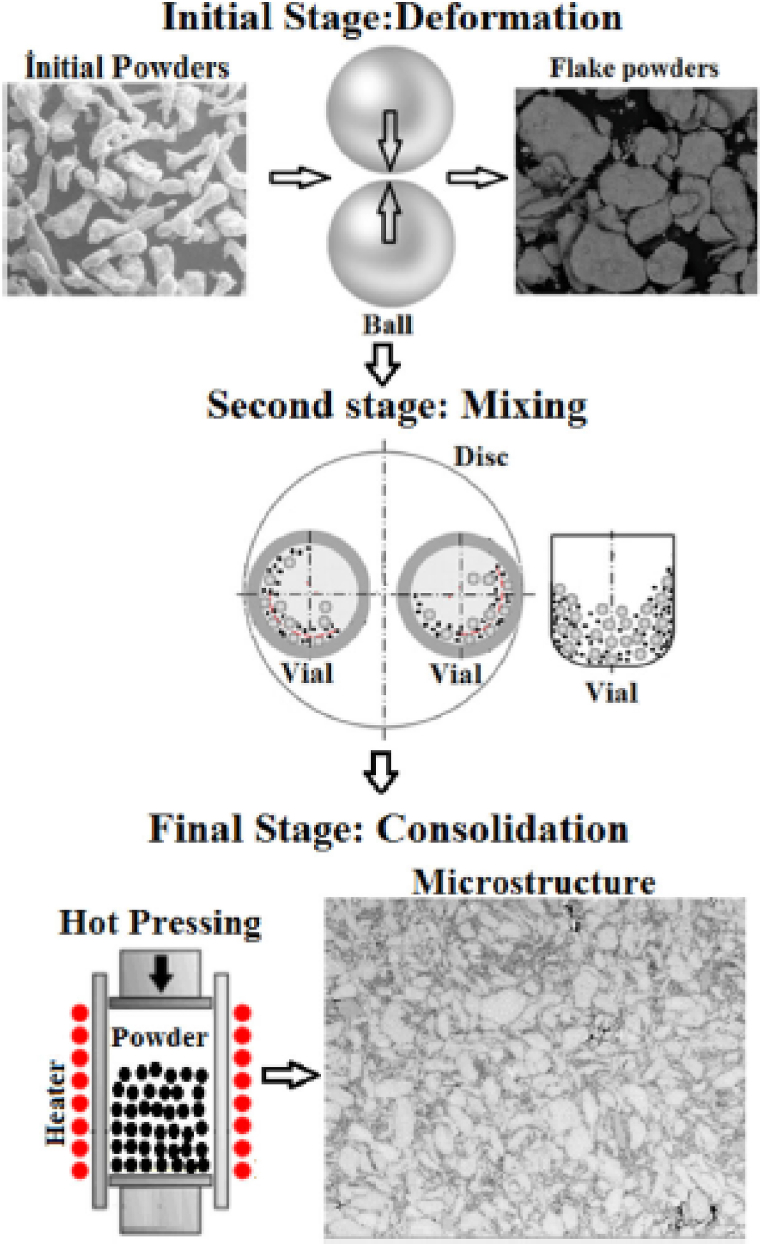
Steps involved in the flake powder metallurgy process [154].

Wang et al. [153] studied the effect of sintering temperature and content of the secondary phase particles (Si_p_) on microstructural and mechanical properties of AA 6061 based composite. The composites were manufactured using a pressureless sintering process. Two different composites i.e. Al 6061 + 30% Si_p_ and Al 6061 + 50% Si_p_ were manufactured. The sintering temperature was varied in the range of 660 °C–720 °C. The microstructure of 6061 + 50% Si_p_ specimens was found to have a few agglomerations of Si particles. Due to Orowan strengthening effects, an initial increase in sintering temperature leads to improve density, bending strength and hardness of both composites. However, after a certain value of sintering temperature, degradation in these properties was reported. Varol et al. [154] manufactured an aluminium based MMC using a flake powder metallurgy process. Fig. 11 shows the steps involved in flake powder metallurgy techniques. The consequence of size and amount of secondary particles on microstructure, relative density and hardness of the manufactured composites was investigated. Due to refinement in grains of matrix phase powder, the density and hardness of the resulting composites were found to improve.

Lin et al. [155] incorporated the vibration stage before cold and hot compaction and investigated microstructure. The vibration stage was found to modify the distribution of SiC particles from a stepwise structure to a smooth distribution of SiC particles. Apart from this, the agglomeration of SiC was lower compared to conventional powder metallurgy. Due to this, enhancement in mechanical properties was also reported. Aydin et al. [156] reinforced magnesium matrix with 10, 20 and 30 wt (%) of TiB_2_ and observed improvement in hardness and wear resistance of composites with the increase in weight fraction of reinforcement particles. The presence of hard particles of TiB_2_ will tend to resist wear and plastic deformation and thus improves the mechanical properties and tribological properties of resulting composites. However, an increase in the weight fraction of reinforcement particles was observed to have more agglomeration instead of the homogeneous distribution of particles. Velez et al. [157] manufactured AMC by reinforcing aluminium matrix with granulated slag (GS) and electric arc furnace dust (EAFD). Comparatively lower green and sintered densities were reported for both composites which were ranging from 2.9 to 3.1 g/cm^3^. The highest compressive strength of 372 MPa and 248 MPa was reported for composites having 15% GS and 10% EAFD. Literature revels that, increase the temperature up to certain value during the sintering is responsible for the enhancement of mechanical properties. However, plasma assisted sintering and microwave sintering are relatively more capable to enhance mechanical properties and new development field in powder metallurgy.

**Fig. 12:**
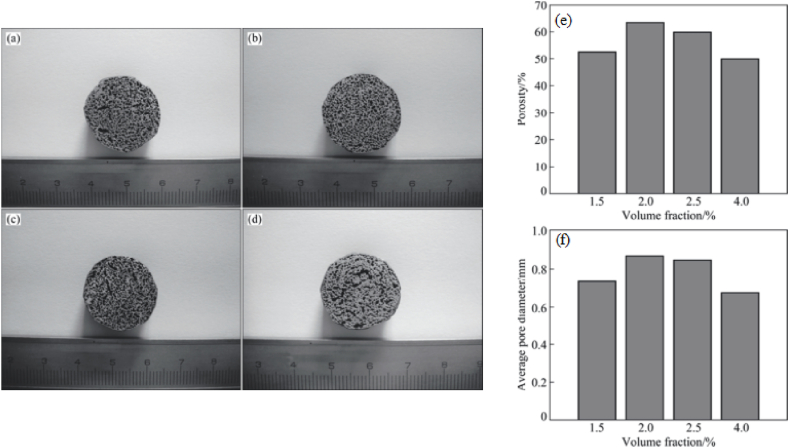
Specimen of AA 2014 + 10% SiC composites pore having (a) 1.5% CaCO_3_, (b) 2% CaCO_3_, (c) 2.5% CaCO_3_, (d) 4% CaCO_3_, (e) plot of porosity % and (f) plot of average pore diameter [160].

Prashanth et al. [158] manufactured glass reinforced aluminium matrix composites and observed that with an increase in the content of reinforcement, the compressive yield strength of the composite was increased but it didn't reach theoretical yield strength. This difference in compressive yield strength was observed due to non-uniform distribution. However, the presence of glass reinforcement particles tends to improve the abrasive wear resistance of the composite. Elkady et al. [159] coated carbon nanotube with either chromium or chromium carbide and avoided the problems related to wettability between matrix and reinforcement particles. As a result of coating, enhancement in properties such as transverse rupture strength, electrical conductivity and densities was reported. Li et al. [160] manufactured AA 2014 + SiC composite foam using CaCO_3_ as the foaming agent. The specimen of foam composite along with the porosity percentage and average pore diameter is shown in Fig. 12 (a) – (f). It was concluded that the addition of CaCO_3_ as a foaming agent fails to increase both the porosity percentage and average pore diameter. Korpe et al. [161] manufactured foams of aluminium composites in which boric acid was used for holding space in the matrix. Fly-ash with weight fractions of 1% and 10% was used as reinforcement particles which were added to the matrix of pure aluminium. By the addition of reinforcement particles, the compressive strength of composite foam was improved by 10% and the addition of boric acid was found to produce porosity of 40%. The plot of the experimental stress-strain curve revealed the elastic region at the initial stage and then the nearly constant flow of stress was reported for strain up to 70%. Beyond 70% of strain, a rapid increase in stress can be observed. Due to the constant flow of stress and densification of stain beyond 70%, improvement in mechanical properties of foam composite was observed.

The range of process parameters and the corresponding value of mechanical properties of various composites manufactured using powder metallurgy are presented in Table 2. For the data represented in Table 2, it can be observed that very few researchers have measured the tensile strength of composites manufactured using powder metallurgy. Most of the research articles focus on the evaluation of microstructure and microhardness of bulk composites.

**Table 2:** Range/magnitude of a process parameter, tensile strength and hardness of different bulk composites manufactured using powder metallurgy.

Sr. No.	Composite	Process parameter and their range or magnitude	Tensile Strength (MPa)	Hardness	Reference
1	Al–Si/SiC_P_ + Al–Si–Cu–Mg	Al–Si/SiC_P_ Powder: 25% Alumix 231 Powder: 75% Mixing Speed: 45 rpm Mixing Time: 24 h Hot Pressing: 3 × 10^−6^ Torr Heating rate: 10 °C/min Pressure: 70 MPa	229	–	[139]
2	Copper + 20% SiC	Sintering Temperature: 950 °C Sintering Time: 3 Hours Sinter-Forged Temperature: 750–850 °C Sinter-Forged Time: 2–6 Hours Sinter-Forging Stress: 50–100 MPa	–	140 HV	[142]
3	Copper + 40% SiC	Sintering Temperature: 1000 °C Sintering Time: 3 Hours Sinter-Forged Temperature: 800–900 °C Sinter-Forged Time: 3–7 Hours Sinter-Forging Stress: 75–175 MPa	–	181 HV	[142]
4	Copper + 60% SiC	Sintering Temperature: 1050 °C Sintering Time: 3 Hours Sinter-Forged Temperature: 850–950 °C Sinter-Forged Time: 4–8 Hours Sinter-Forging Stress: 175–250 MPa	–	253 HV	[142]
5	Cu (Sn) – TiC + Diamond	Ball Milling time: 03 Hours Sintering Temperature: 1000 °C Holding Time: 15 min Vacuum Pressure: 20 MPa	–	–	[162]
6	Copper + TiC + Graphite	Sintering Temperature: 700–850 °C Holding Time: 5–20 min Ramp Rate: 12 °C/min	–	98 HV (5% Graphite)	[163]
7	Ni plated Fe + 10% Al_2_O_3_	Pressure: 300 Bar Sintering Temperature: 1000–1400 °C Sintering Time: 01 h	–	115 HB	[164]
8	Al 2024 + 5% SiC	Ball Milling was performed Ball to Powder ratio: 10:1 Rotational Speed: 300 rpm Milling Time: 15 min Hot Pressing Pressure and Temperature: 150 MPa and 500 °C Soaking Time: 45 min	–	88.65 BHN	[154]
9	Titanium + TiC	Applied Pressure: 225 MPa Vacuum of: 5 × 10^3^ Pa Sintering Temperature: 1350 °C Sintering Time: 04 Hours	–	37 HRC	[165]
10	ZrO_2_ + NiCr	Ball milling for 24 h with ethanol as solvent Forming Pressure: 80–150 MPa Cold Isostatic Pressure: 250–350 MPa Sintering Temperature: 1400 °C Sintering Time: 3 Hours	–	–	[134]
11	AZ 31 + 20% ZrB_2_	Particles Size: Mg – 180 μm, Al – 90 μm, Zn – 50 μm and ZrB_2_ - < 10 μm Ball milling for 3 Hours at 120 rpm Cold Compact Load: 3150 kN Sintering temperature: 420 °C Sintering Time: 3 Hours After Sintering compact was sprayed with dry graphite lubricant at 360 °C for 1 h	–	95 HB	[138]
12	Al 2124 + 40% SiC	Powder Blended for: 1.5 Hours Die Vibration for: 0.75 to 2 Hours Cold Compact Pressure: 300 MPa Compact Heating Temperature: 500 °C Pressing Pressure: 1.5 GPa	–	163 HV	[155]
13	Al_2_O_3_ + 40% ZrO_2_	Ball Milling Time: 8 Hours Rotational Speed of ball mill: 250 rpm Green compact Pressure: 100 MPa Sintering temperature: 1600 °C, 1650 °C and 1700 °C Sintering Time: 2 Hours	–	14.37 GPa (1700 °C)	[141]
14	Al 6061 + 30% Si_p_	Preheating of Powders at 150 °C for 24 h Mixing of Powder for 24 Hours at 500 rpm Green compact Pressure: 400 MPa Sintering Temperature: 660, 680, 700 and 720 °C Sintering Time: 2 Hours	–	110.2 HB (680 °C)	[153]
15	Al 6061 + 50% Si_p_	Preheating of Powders at 150 °C for 24 h Mixing of Powder for 24 Hours at 500 rpm Green compact Pressure: 400 MPa Sintering Temperature: 660, 680, 700 and 720 °C Sintering Time: 2 Hours	–	145.8 HB (700 °C)	[153]
16	AlSi_7_Mg + 2% TiB_2_	K_2_TiF_6_ and KBF_4_ salts used for generation of TiB_2_ using in-situ method Compacting pressure: 165 MPa Compacting time: 60 s Sintering temperature: 560 °C Sintering Time: 2 Hours Heating condition: under vacuum of 10^−3^ MPa	344 MPa	–	[166]
17	AA7075–0.5 wt% Y_2_O_3_	Ball milling of powder for 0.25 Hours Tungsten Carbide ball of 10 mm diameter was used for ball milling Rotational speed: 400 rpm Compacting Pressure: 400 MPa Sintering temperature: 430 °C Sintering Time: 30 min	211.14 MPa	106.91 HB	[167]

## 3 Liquid state processing techniques

Manufacturing of MMC under liquid state processing occurs by melting the matrix phase and adding the secondary phase particles into the molten matrix. The molten mixture will be poured into the mould and thus will result in the formation of a composite having desired matrix and reinforcement phase. Commonly implemented techniques involved under liquid state processing are discussed in subsequent sections.

### 3.1 Manufacturing of composite under the action of centrifugal force

As the name suggests, this is the method in which the distribution of secondary phase particles or intermetallic compounds in the molten matrix will govern by centrifugal force [168]. It was observed that the motion of secondary phase particles in molten metal obeys Stoke's Law [169]. Apparatus involves a crucible with a plunger which will hold molten matrix and a spinning mould located in a mould heating furnace. When the plunger will move in an upwards direction, the molten matrix will enter the spinning mould through the inlet port. Due to the spinning action of the mould, centrifugal force will be generated which will distribute the reinforcement particles in the molten matrix. Post spinning, the mould is removed from the preheated furnace and it is allowed to cool until solidification [170]. Fukui et al. [171] manufactured aluminum based composite which was found to have varying composites of SiC from the inner surface to the outer surface. The measured strain value (i.e. 300 to 450 μ) obtained from the compression test was found to have good agreement with the theoretical value achieved from curved beam theory. Also, the strain generated due to thermal residual stress was measured and this strain was found to engender due to uniform cooling of composite from half of the melting point to room temperature. Tensile residual hoop stress was observed at the inner surface whereas, the outer surface was found to have compressive residual stresses. It was observed that deviation in thermal residual stress increases with the increase in thickness of the composite ring. This deviation will ultimately result in higher thermal mismatch/misfits.

**Fig. 13:**
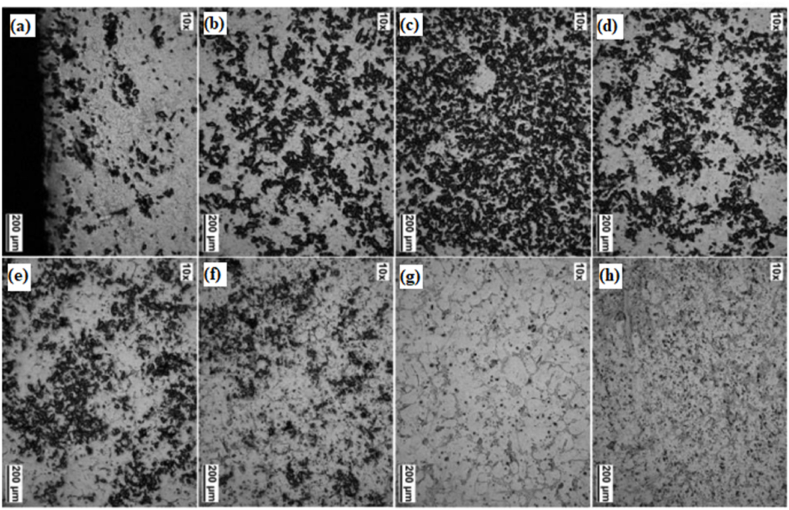
Optical microscope image taken at different locations from (a) 0.5 mm, (b) 12 mm, (c) 16 mm, (d) 20 mm, (e) 40 mm, (f) 46 mm, (g) 66 mm and (h) 82 mm from outer surface cast composite [173].

Watanabe et al. [170] classified the centrifugal method into two distinguished categories dependent upon the relation between working temperature and liquidus temperature of the matrix. The first category under this classification was the centrifugal solid particles method. During this method, the liquidus temperature of the base metal will be much higher than the processing temperature. Throughout the centrifugal solid particles method, the dispersed reinforcement material will remain in the solid state. Adelakin et al. [172] manufactured boron reinforced aluminium matrix composites and observed the gradient distribution of boron particles on the outer zone compared to the inner zone. Along with gradient distribution, the outer surface was also found to have few agglomerations of reinforcement particles. At the same time, scanning electron microscope analysis revealed the presence of a higher volume fraction of AlB_2_ and AlB_12_ on the external zone. The presence of Mg in the molten mixture was found to promote the transformation of AlB_12_ to AlB_2_. This transformation was found to enhance the Rockwell hardness and the Vickers microhardness of the resulting composite. The dominating parameter affecting the distribution of reinforcement particles and viscosity was the pouring temperature of the molten matrix, whereas rotational speed was the least affecting factor. Rajan et al. [173] manufactured Al–SiC ex-situ and Al–Si in-situ composite using centrifugal casting. The microstructure of ex-situ composites shown in Fig. 13 (a) – (h) revealed the gradient distribution of SiC particles from the inner zone to the external zone of the disc. However, Al–Si in-situ showed dispersion of Si particles towards the inner surface and due to the same, the hardness of the inner surface was higher than the external surface of the composite. During the centrifugal in-situ method, the liquidus temperature of the matrix is comparatively lower than that of the working temperature. Thus during solidification, centrifugal force is applied to both i.e. secondary phase as well as the matrix phase. Some of the composites which have been manufactured using this combined method are Al/Al_3_Ni and Al/Al_2_Cu. During the manufacturing of these composites, the liquidus temperature of both Al–Ni and Al–Cu alloys was lower than the processing temperature. Fukui [174] manufactured a hybrid composite Al_3_Ti + Al_3_Ni by combining the effect of centrifugal solid particles and the centrifugal in-situ method. Along with this, an empirical relationship was developed which will provide the gradient distribution of particles as a function of centrifugal force and volume fraction of secondary phase particles. The developed model was validated with experimental results and good agreement between predicted results and experimental results were reported.

Centrifugal slurry casting is another derivative of the centrifugal casting method. In the centrifugal slurry casting method, solid particles in molten metal will have two distinguish velocities i.e. high and low. During the manufacturing of composites, these particles will undergo the effect of centrifugal force. Once complete sedimentation of the particle is achieved, the liquid part of the slurry is taken out and thus it doesn't become a part of the composite. Ti/ZrO_2_ is an example of a composite manufactured using the centrifugal slurry method [169]. However, it should be noted that it becomes difficult to control the velocity of solid particles in the molten matrix which will be moving with higher velocity. This difference in velocity will ultimately affect the dispersion of solid particles in the molten matrix. Thus to avoid such difficulty, Watanabe et al. [175] proposed a novel processing technique named the centrifugal mixed powder method. This method involves the mixing of matrix particles with secondary phase particles in predefined quantities and pouring this mixture into the spinning mould. After pouring mixed powder, the molten matrix will also be poured into the spinning mould. Due to centrifugal force, the molten matrix will fill the empty space between particles. This molten matrix will tend to melt the matrix particles present in mixed powder and at last, the ring structure or disc shape of the composite will have an even distribution of reinforcement material. Yamauchi et al. [176] used the reaction centrifugal mixed powder method for manufacturing Al–Al_3_Ti composite. The reaction between molten Al and Ti particles results in the formation of the Al_3_Ti phase. The difference in disregistry value of Al_3_Ti intermetallic and Al matrix tends to be less than 10% and thus Al_3_Ti intermetallic will acts as a grain refiner. The presence of Al_3_Ti intermetallic acts as good nucleation in the Al matrix which is necessary for refined grain structure. It was observed that the external zone of the cast specimen was characterized by the α-Al phase having fine grain whereas, the inner zone was found to have coarse grains of α-Al. Miura-Fujiwara et al. [177] and Kunimine et al. [178] manufactured composites like Cu/SiC, Al/TiO_2_, Al/Al_3_Ti/Ti, Cu/Diamond and Al/Diamond using the centrifugal mixed powder method. However, the only drawback of this method is that; few types of mixed powder tend to flow away due to centrifugal force. Due to this, proper consolidation of the mixed powder and molten metal doesn't occur.

**Fig. 14:**
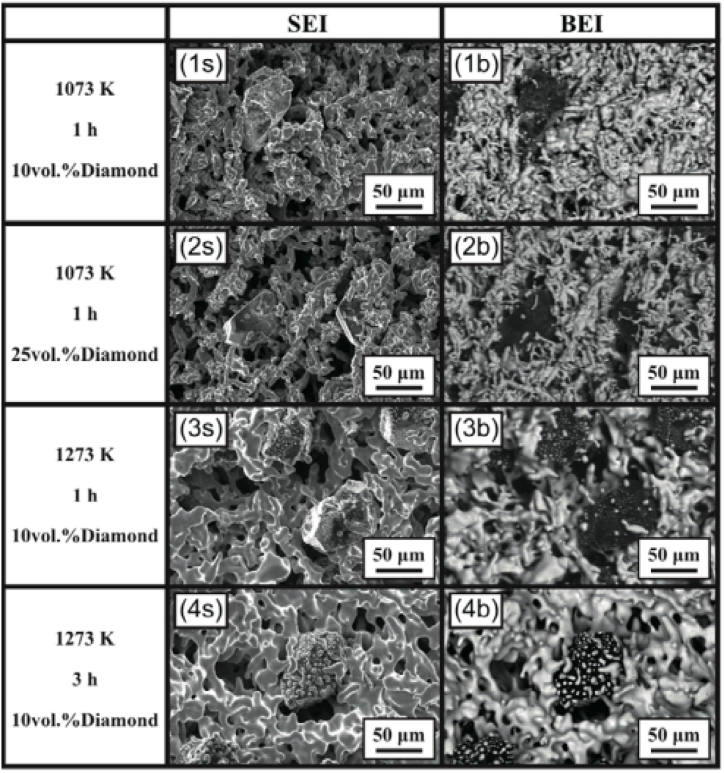
Microstructure images of a copper matrix reinforced with diamond particles (1s) – (4s) SEM-SEI images and (1b) – (4b) SEM-BEI images [179].

Kunimine et al. [179] preheated mixed powder and then this sintered powder was introduced to a spinning mould. This derivative of centrifugal mixed powder was then termed as the centrifugal sintered casting method. The rest of the entire procedure remains the same as mentioned in the centrifugal mixed powder method. Kunimine et al. [179] manufactured copper/diamond composite and reported a reduction in preform thickness with the increase in sintering temperature and holding time. An increase in these parameters will avoid the melting of dendritic shape copper particles. The secondary electron images (SEM-SEI) and backscattered electron compositional images (SEM-BEI) of copper/diamond composite are shown in Fig. 14 (1s) – (4s) and Fig. 14 (1b) – (4b) respectively. From the same, it can be observed that the preform of the composite cast at 1073 K consists of a dendritic shape copper matrix. The shapes of this dendritic copper matrix were similar to primary copper powder. However, it should be noted that when the temperature was increased to 1273 K, a reduction in dendritic shape copper particles was observed. It is believed that these dendritic shape copper undergoes melting and results in a good interfacial bond between copper and diamond particles. Due to the same, a reduction in the number of microscopic defects such as voids and pores was reported. Out of sintering temperature and holding temperature, sintering temperature was the dominating parameter which affects the preform thickness of the cast. It was reported that the volume fraction of diamond particles in the copper matrix was controlled by regulating several process parameters of centrifugal casting. Also, the structures of copper/diamond composite were found to have a definite amount of pores and will act as a chip space. Thus, these composites were used as a grinding wheel for machining carbon fibers reinforced polymers. By using this grinding wheel, precision drilling of carbon fibers reinforced polymer plates was done and thus finds industrial application for machining of carbon fibers reinforced polymer plates.

**Fig. 15:**
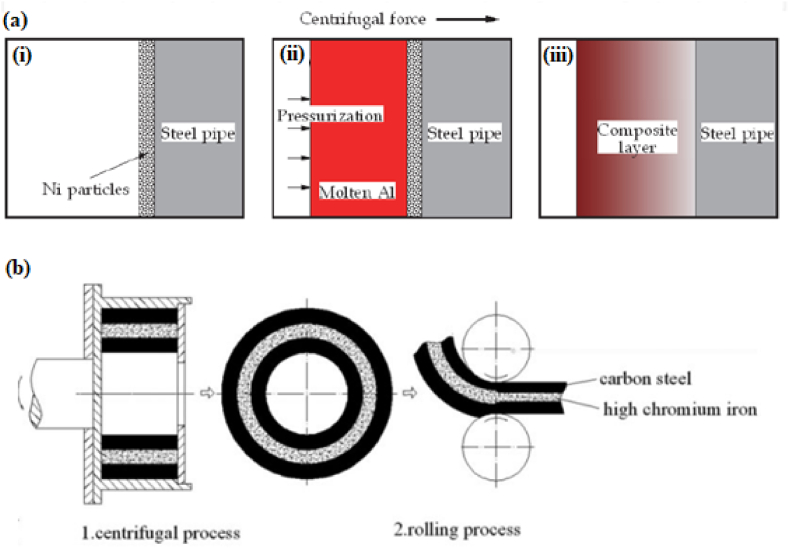
(a) Schematic representation of reactive centrifugal casting method [183] and (b) Schematic diagrams for manufactured sandwich composites [184].

Another novel technique which has been developed by Matsuura et al. [180] is known as the reactive casting method. It works on the concept of exothermic reaction which occurs between elemental liquids. This exothermic reaction generates a liquid which consists of intermetallic compounds having a high melting point. The method doesn't require any external heat source. Matsuura et al. [180] combined the reactive casting method with centrifugal casting and manufactured Ni–Al–Fe composite. Thus, Watanabe et al. [181] termed this process as a reactive centrifugal casting method. A graphical representation of this technique is shown in Fig. 15(a). Fig. 15(a (i)) represents a spinning steel pipe in which Ni powder is injected. The next step represented by Fig. 15(a (ii)) involves the pouring of molten aluminium into the spinning pipe. The pouring of molten aluminium will result in an exothermic reaction occurring between molten aluminium and Ni powder. Due to this exothermic reaction, the inner surface will be found to have a composite layer of Ni–Al and the same has been represented in Fig. 15(a (iii)). Due to exothermic reaction, the inner surface of steel pipe tends to melt and will result in bonding steel and composite layer. The benefit of this process is that it reduces the production cost due to inexpensive Al ingots. Ebhota and Inambao [182] developed a mathematical model which predicts the dispersal of secondary phase particles in the resulting cast composite manufactured using the centrifugal casting technique. The model was developed by considering several parameters such as forces acting on particles, solid/liquid interface forces and melt particles, heat transfer coefficient of composite, thermal conductivity, volume fraction resolution and particles matrix field temperature. The developed model may prove to be helpful for manufacturing composites with gradient/homogenous distribution of reinforcement particles.

**Fig. 16:**
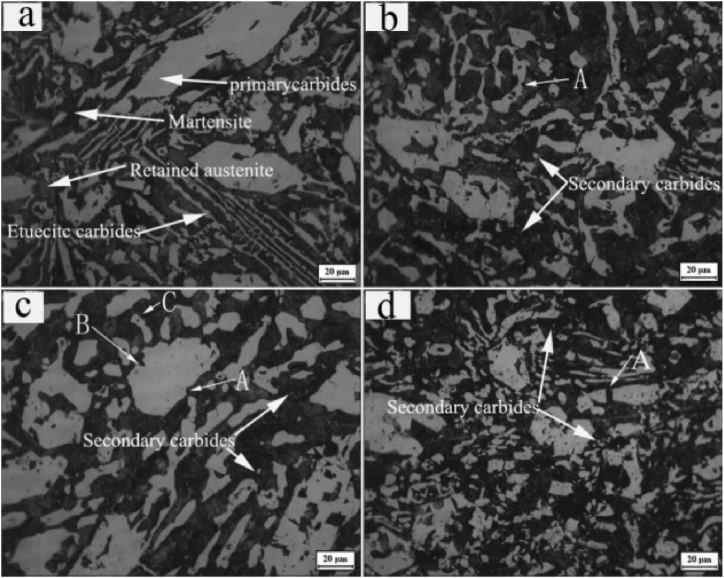
Microstructure of composites before and after hot rolling (a) as cast (b) one pass of hot rolling (c) two passes of hot rolling (d) four passes of hot rolling [184].

Liu et al. [184] used centrifugal casting along with hot rolling to manufacture a sandwich composite having a core of hypereutectic high chromium iron and claddings of low carbon steel. The manufactured composite was further subjected to various heat treatment processes which ultimately improve its performance. The schematic diagram of centrifugal casting followed by hot rolling used for manufacturing sandwich composite is shown in Fig. 15(b). A comparative study between as-cast component, hot rolled component and heat treated component was performed. Good metallurgical bonding between interfaces was observed even before hot rolling. Improvement in hardness was reported when the as-cast component undergoes a hot rolling process however, maximum hardness was observed for air quenched component. Also, the impact toughness observed for the heat treated component was thrice the impact toughness of the as-cast component. The various microstructures of as-cast and hot rolled component is shown in Fig. 16 (a)–(d). Point A in Fig. 16(b) represents the dissolution of carbides which results in the formation of grooves. The curve surfaces of the groove present in Fig. 16(b) were found to have sharp corners in Fig. 16(c) due to the second pass of rolling. After the fourth pass of rolling, the breaking of agglomerated carbide particles can be observed in Fig. 16(d). Radhika and Raghu [185] manufactured Al + AlB_2_ and investigated the hardness, tensile strength and wear properties. Hardness was measured on the inner, middle and outer surfaces; tensile strength was measured on the inner and outer surface and an abrasive wear test was conducted on the outer surfaces of the composite. The consequence of load, rotational speed and size of secondary phase particles on wear rate was investigated. Due to the higher concentration of AlB_2_ particles on the outer surface, tensile strength and hardness were found to degrade when measured from the outer surface to the inner surface of composites. Wear resistance was found to decrease with the increase in load whereas, the same was found to increase with the increase in rotational speed and reinforcement size.

**Fig. 17:**
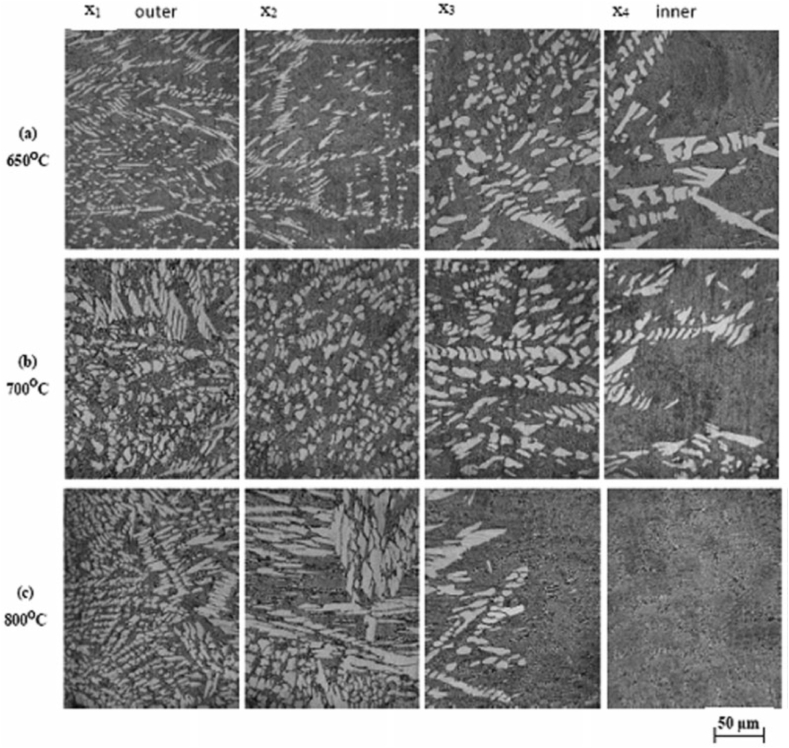
Variation in micrographs of composite manufactured at (a) 650 °C, (b) 700 °C and (c) 800 °C [188].

**Fig. 18:**
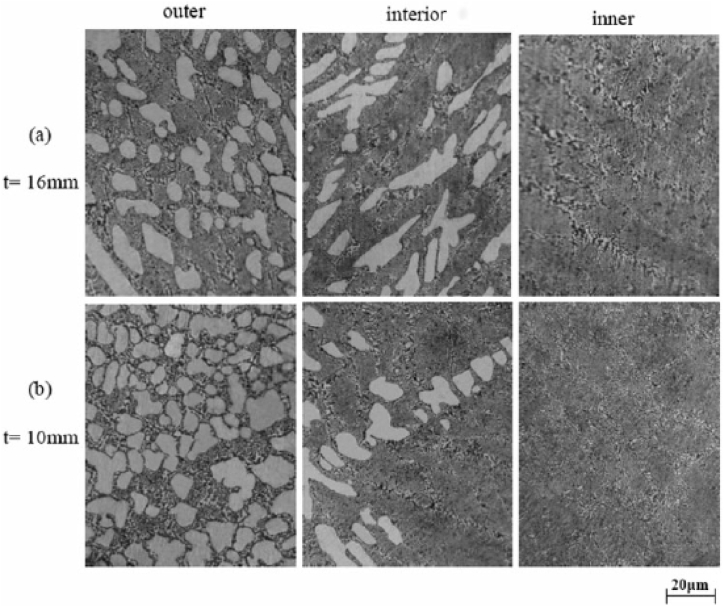
Variation in micrographs of manufactured composite at thickness of (a) 16 mm and (b) 10 mm [188].

Förster et al. [186] adopted centrifugal casting for manufacturing aluminium containing TiB_2_ generated using the in-situ method. It was reported that the increase in the mould rotational speed tends to break the clusters of reinforcement particles and leads to good bonding between matrix and reinforcement particles. Also, a mathematical model was developed which simulates the volume fraction profile of TiB_2_ for different rotational speeds. Zhang et al. [187] manufactured Al–Mg_2_Si in-situ composite and observed inhomogeneous distribution of Mg_2_Si particles along the radial direction. The outer surface was recognized as a rapid cool area and it was reported that in those areas, particles were found to move inwards. Due to the same, the middle part of the composite was found to have particles free regions. With the increase in the content of Mg_2_Si particles, the inner surface was characterized by an agglomeration of particles. Tensile strength was found to increase from the inner to the outer surface of composites. At the same time, it was also reported that 50–70% volume of Mg_2_Si particles tends to degrade the tensile strength of composites. Samadi and Shahbazkhani [188] investigated the dispersal of Al_2_Cu particles and the hardness of manufactured composite by varying pouring temperature and thickness of cast composite. Three different pouring temperatures were 650, 700 and 800 °C and the two different thicknesses of the cylinder were 10 and 16 mm. The micrograph taken at the outer and inner surface of composite manufactured by varying pouring temperatures is shown in Fig. 17 (a)–(c). From Fig. 17, it can be observed that an increase in pouring temperature adversely affects the particle size and distribution. When compared to other pouring temperatures, composites manufactured at 800 °C were found to have the highest size of Al_2_Cu particles. Irrespective of pouring temperature, when observed from outer to inner surface the size of Al_2_Cu particles was found to increase. However, the inner surface of the composite manufactured at 800 °C showed an absence of Al_2_Cu particles. It is a well-known fact that homogenous distribution and grain refinement play a crucial role towards the enhancement of mechanical properties. Higher pouring temperatures will lead to a lower rate of solidification. During solidification, enlargement in Al_2_Cu particles was observed and the particles were found to leave the inner surface of the composite. Due to the same, Al_2_Cu particles were more concentrated and segregated on the outer surface of composites. As shown in Fig. 18 (a) and (b), with the variation in thickness from 16 mm to 10 mm the volume fraction of Al_2_Cu particles increases.

Due to several reasons, centrifugal casting finds its application in industries for manufacturing rings, hollow cylinders, tubes, pipes and large motor housing. Industries use this casting technique as it eliminates the use of core and the wall thickness can be regulated just by controlling the volume of molten metal during the process. Also, rollers or bushings can be manufactured with high concentricity, straightness and uniform thickness [189]. Presently, functionally graded materials having porous structure finds several applications in industries. Also, these porous graded materials find application as medical implants, thermal protection for re-entry of space vehicles, thermos-electrical converter for energy conversion and many more [190]. The data of process parameters, hardness and tensile strength of composites manufactured using the centrifugal casting method provided in Table 3 may prove helpful to the industries dealing in the aforementioned sectors. The limitation of various processing methods under the effect of centrifugal force is that it results in the manufacturing of only cylindrical shapes i.e. disc, rings or pipe like structures. Apart from this, the cast composites manufactured using centrifugal casting techniques are normally restricted to a length to diameter ratio of around 2:1. If this ratio is exceeded, the molten composite tends to have uneven thickness due to lack of centrifugal force. Lastly, developing a proper database for composites i.e. material systems, control parameters, material preparation, performance evaluation and long term reliability along with repeatability are some other limitations [[191], [192], [193], [194]].

**Table 3:** Range/magnitude of a process parameter, tensile strength and hardness of different functionally graded composites materials manufactured using various centrifugal casting methods.

Sr. No.	Composite	Process parameter and their range or magnitude	Tensile Strength (MPa)	Hardness	Reference
1	A 319 + SiC (Heat treated)	Particle Addition Temperature: 730 °C to 740 °C Mould Rotation Speed: 250 rpm Stirring Time: 20 min (after particles were added)	270	87 HRB	[195]
2	356 Cast Aluminum + 15% SiC (Heat treated)	Slurry Temperature: 750–760 °C Mould Temperature: 250 ± 10 °C Rotational Speed: 1100 rpm	–	155 BHN	[196]
3	2124 Wrought Aluminum + 15% SiC (Heat treated)	Slurry Temperature: 750–760 °C Mould Temperature: 250 ± 10 °C Rotational Speed: 1100 rpm	–	145 BHN	[196]
4	Aluminum + Mg_2_Si + Si in-situ (T6 Treated)	Slurry Temperature: 760 °C Mould Temperature: 150 °C Rotational Speed: 3000 rpm	247 (at 100 °C)	–	[197]
5	Aluminum (Zl 104) + 20% SiC	Slurry Temperature: 750 °C Mould Temperature: 500 °C Rotational Speed: 800 rpm	–	122 HB	[198]
6	AlSi18CuMgNi + 17.2% SiC	Slurry Temperature: 850 °C Mould Temperature: 600 °C Rotational Speed: 800 rpm	–	23.7 HRB	[199]
7	Aluminum + SiC Aluminum + SiC + Mg_2_Si in-situ	Slurry Temperature: 840 °C Mould Temperature: 220–250 °C Rotational Speed: 220–250 rpm	–	45 HRB 56 HRB	[200]
8	Iron + Tungsten Carbide	Slurry Temperature: 1300 °C Mould Rotational Speed: 1340 rpm	–	–	[201]
9	Al–12Si –Cu + 12% B_4_C Al–12Si –Cu + 12% SiC Al–12Si –Cu + 12% Al_2_O_3_ Al–12Si –Cu + 12% TiB_2_	Particles Preheating temperature: 300 °C Stirring Speed: 200 rpm Mould Temperature: 350 °C Mould Rotational Speed: 1300 rpm	228 230 233 248	1.314 GPa 1.412 GPa 1.415 GPa 1.569 GPa	[201]
10	Aluminum + 15% Mg_2_Si (in-situ)	Mixture of NaCl, NaF and KCl in ratio of 15:35:10 was added to molten surface for refinement of grains. Slurry temperature: 800 °C Mould Rotational Speed: 1800 rpm	242	–	[187]
11	Aluminum + 6% (vol.) TiB_2_ (in-situ)	Slats used for in-situ generation of TiB_2_: KBF_4_ and K_2_TiF_6_ Slurry Temperature: 750 °C Mould Rotational speed: 180, 300, 500 and 700 rpm	–	–	[186]
12	Aluminum (LM 25) + 10% AlB_2_ (Centrifugal Casting + Stir Casting)	Particle Size: 74 μm Stirring Time: 15 min Stirring Speed: 230 rpm Mould rotational speed: 1220 rpm	184	152 HV	[185]
13	Aluminum + Al–B + Al–Mg	Melting Temperature: 850 °C Holding time: 15 to 25 min Mould Preheating temperature: 500 °C Casting time: 1 to 3 min Mould Rotational Speed: 300 to 400 rpm Pouring temperature: 690 to 820 °C	–	88 HV (400 rpm and 820 °C)	[172]
14	AZ91 + 10% SiC	Matrix melting temperature: 620–700 °C Heating temperature of reinforcement particles: 200 °C Mixture stirred for 10 min at 250 rpm Centrifugal casting in presence of argon gas	217 MPa	107 HV	[202]
15	Al + 15 wt %SiC	Melting Temperature: 670 °C Pouring Temperature: 725 °C Stirring Speed for mixing matrix and reinforcement: 100 rpm Rotational speed of mould: 800 to 1000 rpm	152 MPa	56 HB	[203]

### 3.2 Stir casting method

To avoid the aforementioned drawback of the centrifugal casting method researcher often adapts the stir casting method. A comparison carried out by Taha [21] and Surappa [204] revealed that stir casting is better in every aspect compared to several processes under the action of centrifugal force. This casting process is feasible for industrial purposes as it manufactures composite having complex shapes at a comparatively lower cost [[205], [206], [207], [208], [209]]. The dispersion of secondary phase particles into the molten phase of the matrix is governed by a stirrer which is externally driven by the motor placed vertically above it [210]. Kumaran et al. [211] and Hashim et al. [212] reported that the stirring action not only transfers the secondary phase particles in the molten matrix but also tends to rearrange them. The reinforcing material is directly added to the vortex created due to the stirring of the molten matrix. Various researchers suggested preheating of reinforcement particles to reduce the humidity content and avoid the thermal mismatch between the matrix and reinforcement particles [210,213,214].

There are various parameters such as stirring speed, stirring time, melting temperature, holding temperature, stirrer design and many more [[215], [216], [217], [218]]. Results reported by various researcher showed that stirring speed significantly dominates the dispersal of reinforcement particles in molten matrix along with the formation of casting defects. The higher stirring speed will lead to homogenous distribution of reinforcement particles. On the other side, low stirring speed increases the bonding between the reinforcement and matrix but leads to generation of casting defects [219,220]. Another crucial parameter of stir casting process is stirring time. The increase in stirring time, increase the possibility of uniform distribution of reinforcement particles and thus results in higher mechanical properties. However, lower stirring time increases the chances of formation of clusters/agglomeration of reinforcement particles [221,222]. The melting temperature dominated the viscosity of the molten matrix whereas, holding temperature affects the reaction and bonding between the reinforcement particles. However, holding temperature beyond certain limits will promote the formation of deleterious phases as a result of reaction between matrix and reinforcement [223,224]. Lastly, the number of stirrer blades and angle between blades dominates the flow pattern generated in molten mixture [225,226].

**Fig. 19:**
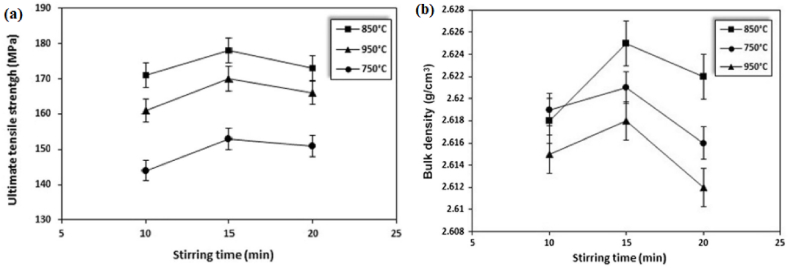
Effect of different pouring temperatures and stirring time on (a) ultimate tensile strength and (b) bulk density [228].

**Fig. 20:**
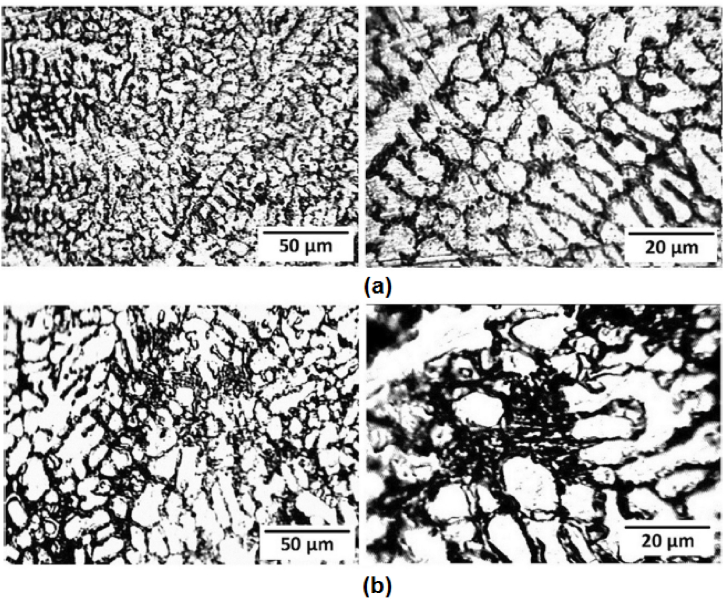
Optical Microscopy of as-cast composites constant stirring time of 15 min and (a) pouring temperature 850 °C and (b) pouring temperature 950 °C [228].

Mollaei et al. [227] manufactured Al/SiO_2_ nano composites and investigated the variation in microstructure, mechanical and tribological properties by varying pouring temperature and stirring time. A comparative study between reinforced composite and aluminium alloy was also reported. Initially, Al–Si alloy and SiO_2_ nano particles powder were mixed in presence of argon gas using a planetary ball mill machine. Then, this prepared mixture was heated in a furnace and reinforcement powders were added to the molten matrix. It was observed that by using powders of respective matrix materials and reinforcement materials, agglomeration in microstructure was less than 100 μm. On the addition of SiO_2_ nano particles in the molten matrix at higher temperature i.e. 800-850 °C, enhancement in amount and size of Al–Ni intermetallic phase was reported. Due to the same, the mechanical properties of those composites were higher. Enhancements in wear resistance, hardness and elastic modulus were reported due to the homogenous distribution of SiO_2_ nano particles. A reduction in porosity was observed when nano composites were manufactured by maintaining the pouring temperature at 750 °C and varying the stirring time from 2 min to 4 min. Khademian et al. [228] investigated the variation in microstructure and mechanical properties of A356/B_4_C composites by varying pouring temperature and stirring time. It was observed that for constant pouring time, an increase in pouring temperature initially tends to enhance mechanical properties. However, after a specific point, an increase in pouring temperature was found to degrade the mechanical properties. The effect of stirring time and pouring temperature on tensile strength and bulk density is shown in Fig. 19 (a) and (b). The optimum results were observed for a pouring temperature of 850 °C and a stirring time of 15 min. The optical microscopy images of composites manufactured by stirring time of 15 min and varying pouring temperature is shown in Fig. 20 (a) and (b). While comparing both figures, it can be observed that an increase in pouring temperature from 850 °C to 950 °C reduces the grain refinement of matrix reinforcement particles. Also with a higher pouring temperature, the formation of dendritic structure along with agglomeration of reinforcement particles can be observed. Also with a higher pouring temperature, the formation of dendritic structure along with agglomeration of reinforcement particles can be observed. It was also reported that the high pouring temperature and long stirring time supports the chemical reaction which leads to the formation of undesirable compounds. This undesirable chemical compounds and dendritic structure along with the agglomerated particles reduce the mechanical properties of manufactured composites. Khademian et al. [228] also investigated the consequence of hot rolling and hot extrusion on the mechanical properties of manufactured composites. Due to the same, reduction/refinement in size of pores and defects were observed and thus enhancement in mechanical properties was reported. Furthermore, this deformation process tends to modify the microstructure of manufactured composites. Due to hot rolling and hot extrusion, clusters of secondary phase particles were observed to be broken up and were homogenously distributed across the matrix phase. On performing mechanical testing, the hot extruded specimens were found to have superior hardness and tensile strength compared to that of hot rolling. Naher et al. [218] in their study attempted to optimize process parameters. A mixture of glycerol and water was created so that it mimics the molten aluminium as the viscosity of this mixture was similar to that of molten aluminium. Particles of SiC were then added to this mixture. It was observed that a stirrer or impeller with four or three blades which are at an angle of 60° or a turbine blade impeller tends to distribute reinforcement particles homogeneously.

**Fig. 21:**
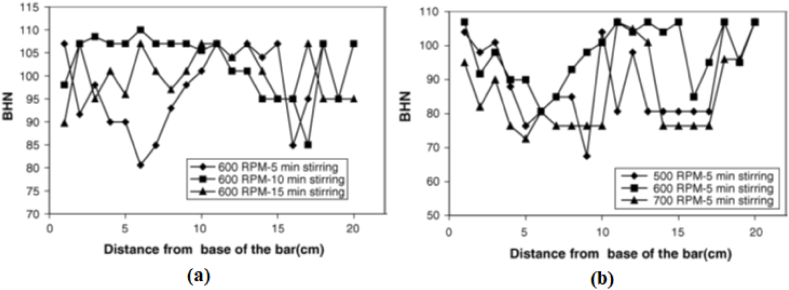
Variation in hardness of Al–SiC composites manufactured by (a) a constant stirring speed of 600 rpm and varying stirring time from 5 min to 15 min and (b) a constant stirring time of 5 min and varying stirring speed from 500 rpm to 700 rpm [232].

Sozhamannan et al. [229] from their study reported that melting temperature and holding temperature dominate the wettability and particle distribution. If the temperature of the molten matrix is higher than the melting point of the matrix, then it will have low viscosity. This lower viscosity of the molten matrix will improve the distribution of secondary phase particles and will also provide better retention of particles. Ezatpour et al. [230] suggested that wettability can also be improved by removing the gas layer from the top side. Hashim et al. [212] reported that wettability can be improved by providing stirring action such that it overcomes surface tension. Ghosh et al. [231] manufactured Al_2_O_3_ reinforced composites and reported that wettability can be improved with the stirring speed of 960 rpm, stirrer height of 0.81, stirrer diameter of 0.63 and holding temperature between 605 and 615 °C. However, Naher et al. [218] also reported that excessive stirring reduces wettability. Prabu et al. [232] studied the dispersal pattern of SiC particles in molten aluminium by varying stirring speed and stirring time. They reported that for Al–SiC composites, the homogenous distribution of SiC particles can be obtained for a stirring speed of 600 rpm for a time period of 10 min. From the plot of hardness represented in Fig. 21(a), it can be observed that the increase in stirring time from 5 min to 10 min ultimately enhances the hardness of composites. However, no subsequent improvement in hardness was observed for the increase in stirring time beyond 10 min. Similarly, from Fig. 21(b), it can be found that the hardness increases with the increase in the stirring speed from 500 rpm to 600 rpm. Whereas, the hardness was found to degrade when the stirring speed exceeds 600 rpm.

**Fig. 22:**
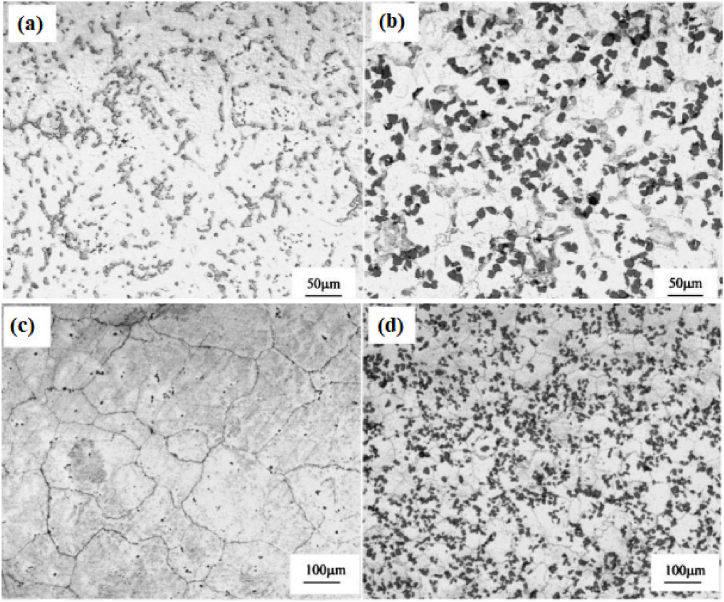
Microstructure of (a) Mg–Al_9_Zn magnesium alloy, (b) as-cast Mg–Al_9_Zn/15%SiC composite, (c) T4 treated Mg–Al_9_Zn magnesium alloy and (d) T4 treated Mg–Al_9_Zn/15%SiC composite [234].

**Fig. 23:**
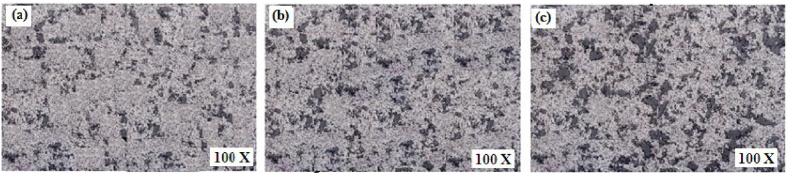
Microstructure of aluminium 356 reinforced with (a) 5% (b) 10% and (c) 15% of SiC [235].

Das et al. [233] examined the consequence of different particles size of zircon sand incorporated in the aluminium matrix. In comparison with finer particles, coarsen particles were found to have a substantial amount of dispersion in the aluminium matrix. Also, it was reported that abrasive wear resistance improves when the volume of the reinforcement particles increases or particle size decreases. Gui et al. [234] manufactured magnesium based composite using a vacuum stir casting process. A comparative study between the microstructure of as-cast composite and heat treated composite was carried out. The microstructure of both i.e. Mg–Al_9_Zn magnesium alloy and as-cast Mg–Al_9_Zn/15%SiC composite is shown in Fig. 22 (a) and (b). While comparing Fig. 22 (a) and (b) with Fig. 22 (c) and (d) it can be observed that by providing T4 heat treatment, the distribution of SiC particulates became more homogenous. Dwivedi et al. [235] used the electromagnetic assisted stir casting process for manufacturing aluminium alloy 356 reinforced with SiC particles. The major advantage of this process was that it leads to grain refinement and tends to have more homogenously distributed secondary phase particles compared to the conventional stir casting process. The same can be observed in Fig. 23 (a) – (c). Also, composites manufactured using electromagnetic stir casting was found to have lower porosity. Due to lower porosity, significant improvement in mechanical properties such as tensile strength, hardness, toughness and fatigue strength was reported.

**Fig. 24:**
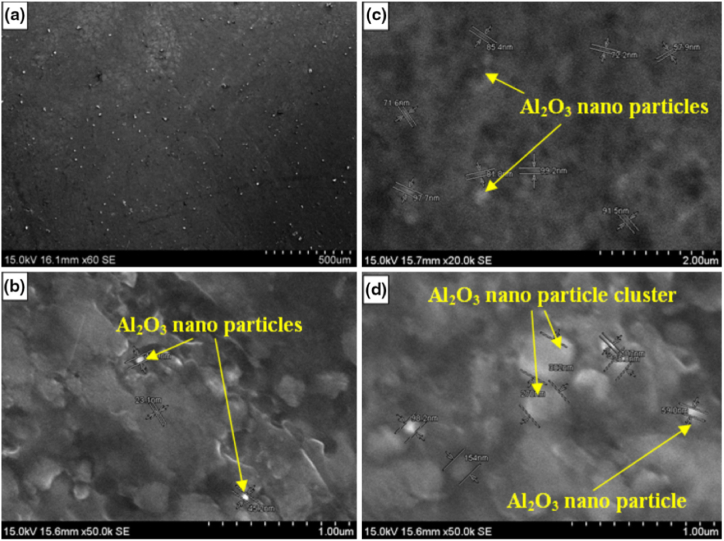
Scanning electron microscope images of (a) as-cast A356 (b) A356 + 0.5% Al_2_O_3_ (c) A356 + 1% Al_2_O_3_ and (d) A356 + 1.5% Al_2_O_3_ [238].

Bharath et al. [236] performed two stage stir casting and manufactured aluminium 2014 based composite reinforced with 9% and 12% of Al_2_O_3_. Scanning electron microscope images of manufactured microstructure revealed the presence of Al_2_O_3_ particles which were uniformly distributed within the α-Al matrix. Along with this, defect free microstructure with the presence of a few agglomerations of Al_2_O_3_ particles was also observed. In comparison with the ordinary stir casting process, two stage stir casting process tends to improve the dispersion of secondary phase particles in the molten matrix as well as enhance the mechanical and tribological properties of cast composite. Similar results were also reported by Nagaral et al. [237] for AA 2014 + ZrO_2_ composites manufactured using two stage stir casting. Sekar et al. [238] combined the stir casting process with squeeze casting and manufactured aluminium alloy reinforced with Al_2_O_3_ particles. From Fig. 24 (a) – (d), it can be observed that the increase in the weight percent of reinforcement particles ultimately results in the formation of clusters or agglomeration of particles. This cluster formation was found to affect the wear properties of the manufactured composites. Dry wear studies reveal that composites with 0.5% and 1% of Al_2_O_3_ were found to have lesser wear loss compared to composites having 1.5% of Al_2_O_3_.

Juang et al. [239] examined the effect of the preheating and addition rate of fly ash in aluminium matrix composites. It was reported that porosity and cluster formation can be avoided or reduced by increasing preheating temperature and reducing the rate of addition of fly ash. Wang et al. [240] examined the interfacial characteristic of SiC/AZ91 composite and reported various phases such as Al_4_C_3_, MgO, and Mg_2_Si which were generated due to interfacial reactions. Three distinct types of interfaces were observed which were named Type I, Type II and Type III. The Type I interface surface revealed direct contact of interface product with the surface of SiC particles, whereas the Type II interface surface didn't reveal anything contact of interface product with the surface of SiC. Type III was only found to have two surfaces which were formed as a result of the reaction between matrix and reinforcement particles. Sekar et al. [241] combined the squeeze casting and the stir casting process to manufacture A356 reinforced with Al_2_O_3_ and molybdenum disulfide and observed the presence of agglomeration of reinforcement particles in hybrid composites. Singh et al. [242] used stir casting and manufactured aluminium based composite in a vacuum mould. The particle size of Al_2_O_3_ and SiC were varied and it was reported that the highest wear resistance was observed for samples having a single size of particles i.e. 100 μm.

Agarwala and Dixit [217] used the bottom pouring concept which avoids the impurities present on the top surface of the molten mixture. Similarly, Sekar et al. [243] and Rawal et al. [244] used the bottom pouring concept and observed a reduction in pouring time and also impurities were avoided. Singh et al. [245] developed a novel technique for the production of composites using the stir casting method. The route of stir casting was consisting of a melt-stir-squeeze-bottom pouring setup which leads to the production of composites. Composites such as A356/SiC nano particles, AA 6082/(Si_3_N_4_+Gr), AA 2024/B_4_C, AA 7075/(SiC + Al_2_O_3_), AA 6061/SiC, Aluminium LM_4_/Tungsten Carbide (WC), Al/SiC, Al/(SiC + MoS_2_) and many more were manufactured using the stir casting method [[246], [247], [248], [249]]. Suthar and Patel [250] critically analyzed the issues such as particle distribution, wettability and porosity faced during manufacturing composites.

Due to the prolonged liquid-reinforcement contact involved in the stir casting process, a considerable amount of interfacial reaction takes place. This interfacial reaction generates several interfacial products which tend to degrade the mechanical properties of the stir cast composites. For instance, during the manufacturing of Al–SiC composites, the interfacial reaction between matrix and reinforcement particles tends to generate the Al_4_C_3_ phase. This Al_4_C_3_ phase reacts with moisture present in the atmosphere and ultimately degrades the mechanical properties of the manufactured composites. However, it should be noted that Shorowordi et al. [251] and Pech-Canul et al. [252] suggested several ways for minimizing the formation of the Al_4_C_3_ phase. The formation of the deleterious interfacial phase can be controlled by (i) modifying the chemical composition of the matrix, (ii) controlling the process parameters of the stir casting process and (iii) surface modification of reinforcement by coating or passive oxidization. It has been reported that the presence of silicon plays a crucial role in the enhancement of several characteristics of Al–SiC composites. The presence of silicon as alloying element retards the kinetics of chemical reaction which leads to the formation of the unwanted phase of Al_4_C_3_ and Al_4_SiC_4_.

It should be noted that the stir casting process is still under research phases and thus doesn't find many industrial applications. However, Table 4 will prove helpful for considering the range of dominating process parameters of the stir casting process. Apart from this, Table 4 also provides the observed tensile strength and hardness of bulk composites manufactured by various researchers. Several researchers and academicians are working on various aspects of the stir casting process with prime focus on (i) analyzing the microstructure, (ii) evaluating the mechanical and tribological properties, (iii) consequence of the process parameters and their optimization, (iv) machinability, (v) consequence of the size of the secondary phase particles and many more.

**Table 4:** Range/magnitude of a process parameter, tensile strength and hardness of different bulk composites manufactured using the stir casting method.

Sr. No.	Composite	Process parameter and their range or magnitude	Tensile Strength (MPa)	Hardness	Reference
1	Aluminum + Al_1_Fe_4_ in-situ (Stir Casting + Hot Rolling)	Melting Temperature: 760 °C to 800 °C Stirring Time: 06 min Stirring Speed: 400 rpm	163.1–185.2	60.1–82.7 HV	[253]
2	AA 6061 + 5% B_4_C AA 6061 + 10% B_4_C	Melting Temperature: 850 °C Particles Preheating Temperature: 250 °C Die Preheated at: 600 °C Stirring Time: 10–15 min Stirring Speed: 400 rpm Stirring Temperature: 800 °C	128 143	64 HV 68 HV	[213]
3	AA 7075 + 6% SiC	Pouring Temperature: 720 °C Stirring Time: 10 min Stirring Speed: 400 rpm	298	109 HV	[254]
4	AA 6063 + 5% B_4_C 1545 K + 5% B_4_C	Stirring Speed: 450 rpm Stirring Temperature: 900 °C	440 455	49 ± 2 HB 67 ± 2 HB	[255]
5	Al + 5% Cu Al + 10% Cu Al + 15% Cu	Melt temperature: 720 °C (for pure aluminium) Degassing agent: Coverall Degassing time: 01 min Inert atmosphere: Argon gas Pressure of argon gas: 10–15 MPa Stirrer Rotational speed: 700–750 rpm Position of stirrer: 1″ height from the bottom of crucible and centrally located. Mould preheating temperature: 200 °C Particles preheating temperature: 200 °C	45 MPa 47 MPa 35 MPa	–	[256]
6	A 356 + 1.5% SiC	Melting Temperature: 800 °C Particles Preheating Temperature: 800 °C Particles Preheating time: 1 h Stirring Time: 7 min Stirring Speed: 600 rpm	479	–	[257]
7	AA 6061 + 31% B_4_C AA 1100 + 31% B_4_C	Melting Temperature: 750 °C Preheating Temperature of Particles: 400 °C Preheating Time: 2 Hours Particles addition rate: 1000 g/min Stirring Time: 15 min Stirring Speed: 550 rpm	340 160	–	[214]
8	AA 2024 + 1.5% Ni AA 2024 + 3% Ni AA 2024 + 4.5% Ni	Stirring Speed: 300 rpm Stirring Time: 20 min Stirring Temperature: 750 °C Homogenization Heat treatment at 500 °C for 24 h		115.36 ± 4.3 VHN 144.05 ± 7.4 VHN 118.54 ± 4.6 VHN	[258]
9	AA 6061 + 5% B_4_C AA 6061 + 10% B_4_C AA 6061 + 15% B_4_C	Stirrer Speed: 800 rpm Matrix Melting Temperature: 700 °C Stirring Time: 10 min	–	–	[259]
10	Aluminum + SiO_2_	Pouring Temperature: 700 to 850 °C Stirring Time: 2 to 4 min	–	172 VHN	[227]
11	Mg–Al_9_Zn + 15% SiC (Vacuum Stir Casting + T4 Heat Treatment)	Melting Temperature: 700–720 °C Particles Preheating Temperature: 250 °C Particles Preheating time: 3 Hours Stirring Speed: 1500 rpm Stirring Time: 25 min Stirring Temperature: 600 °C	218	–	[234]
12	Mg–Zn5Zr + 15% SiC (Vacuum Stir Casting + T4 Heat Treatment)	Melting Temperature: 700–740 °C Particles Preheating Temperature: 250 °C Particles Preheating time: 3 Hours Stirring Speed: 1500 rpm Stirring Time: 25 min Stirring Temperature: 600 °C	210	–	[260]
13	A 356 + B_4_C (Stir Casting + Hot Rolled + Hot Extruded)	Pouring Temperature: 750, 850 and 950 °C Stirring Speed: 300 rpm Stirring Time: 10, 15 and 20 min	352	103 HB	[228]
14	A 356 + 10% Rice Husk Ash (RHA) + 10% Fly Ash (Double Stir Casting)	Particles Preheating Temperature: 250 °C Melting Temperature: >650 °C Stirring time: 10 min +15 min Stirring Speed: 400 rpm Slurry Temperature: 720 °C	–	96 HV	[261]
15	Mg + 12% B_4_C (Stir Casting + T4 Heat Treatment)	Melting Temperature: 680 to 700 °C Preheating Temperature of Particles: 350 °C Stirring Speed: 400 rpm Stirring Time: 20 min	–	73 HV	[245]
16	Mg + 8% SiC + 2% Al_2_O_3_ + 1% Gr Mg + 4% Al_2_O_3_ + 2% SiC + 1% Gr (Gas Injection Stir Casting)	Magnesium Preheating Temperature and Time: 450 °C and 02 Hours Particles Preheating Temperature and Time: 1100 °C and 2 Hours Melting Temperature: 710 °C Stirring Temperature: 650 °C Stirring Speed: 300 rpm	230 189	65 HV 86 HV	[262]
17	AA 6061 + 15% SiC	Stirrer Speed: 100, 200, 300, 400 and 500 rpm Stirring Time: 5, 10, 15, 20 and 25 min Blade Angle: 0, 15, 30, 45 and 60° Casting temperature: 630, 730, 830, 930 and 1030 °C Particles Feed Rate: 30 g/min Particles preheating temperature: 600 °C Preheating time: 60 min Die Preheating Temperature: 250 °C Number of Blades: 3	190–230	–	[263]
18	A 356 + 15% SiC (Electromagnetic Stir Casting)	Melting Temperature: 650 °C Stirring time: 7 min Stirring Speed: 210 rpm	313.5	108 BHN	[235]
19	AA 6061 + 15% Al_2_O_3_ (Stir Casting + Extrusion)	Reinforcement particles were injected into melt by using Argon gas Injection Time: 10–30 min Stirring Temperature: 750 °C Stirring Time: 15 min Stirring Speed: 450 rpm	397	153 BHN	[208]
20	AA 6061 + 20% B_4_C	Melting Temperature: 780 °C Particles Preheating Temperature: 200 °C to 700 °C Stirring Speed: 650 rpm Particles Addition Rate: 40 g/min Mould Preheat temperature: 300 °C	–	106 HV	[264]
21	AA 7075 + 5% Graphite + 6% Bagasse-Ash	Melting Temperature: 750 °C Stirring Speed: 200–300 rpm Stirring Time: 15 min	299.4	99.6	[265]

### 3.3 Squeeze casting method

This process makes use of reusable die wherein solidification of molten composite is done under the action of high pressure. The squeeze casting process is also referred to as liquid metal forging, extrusion casting, liquid pressing, pressure crystallization and squeeze forming [[266], [267], [268]]. The squeeze casting involves the forging of molten metal present in preheated and lubricated die. Once the molten metal starts freezing, the load will be applied and will be maintained until solidification [266]. The dominating process parameters of squeeze casting process are squeezing pressure and pouring temperature. The squeezing pressure affects both microstructure and mechanical properties of the resulting composites. The increase in squeezing pressure leads to the fine dendrites, reduces the spacing between dendrites and avoids clusters of reinforcement particles. Apart from this, increase in the squeezing pressure enhances the tensile properties along with elongation to failure. Unlike stir casting process, squeeze casting process needs lower fluidity of molten metal. Thus, it is beneficial to have lower pouring temperature as it will reduce the fluidity of molten metal and filling will be achieved by pressurization only [[269], [270], [271], [272]]. Depending upon the application of the pressure, squeeze casting can be categorized as direct squeeze casting and indirect squeeze casting. As far as direct casting is concerned, the application of pressure will be directly on solidifying cast. By using piston cylinder assembly, the molten material can also be injected into the mould [273]. On the other side, in the case of indirect squeeze casting the application of pressure is through an intermediate feeding system [274].

**Fig. 25:**
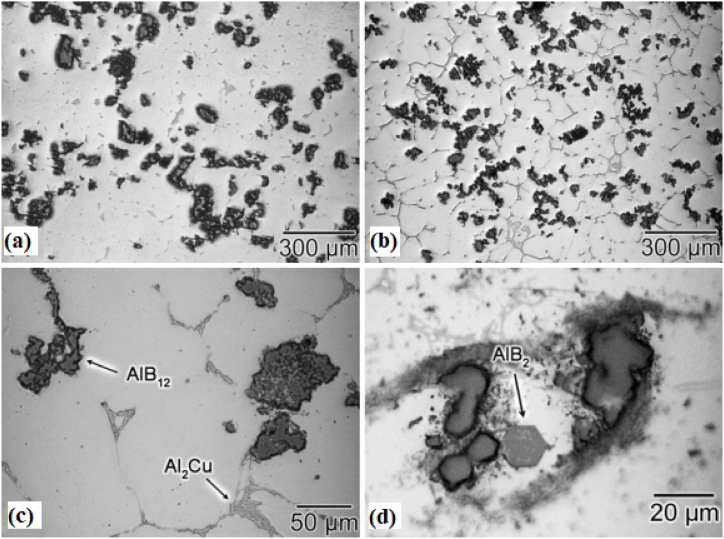
(a) Optical microscopy of the bottom surface, (b) Optical microscopy of top surface, (c) phases observed in composites manufactured with 62 MPa pressure and (d) phases observed in composites manufactured with 31 MPa [275].

Luengas et al. [275] used indirect squeeze casting for manufacturing aluminium matrix composites reinforced with 2% and 4% of boride particles. The bulk density reduces with an increase in squeeze pressure from 0 to 31 MPa whereas, the further increase in squeeze pressure from 31 MPa to 62 MPa, revealed an enhancement in bulk density. This enhancement in bulk density indicates that the composites had experienced a reduction in shrinkage porosity, which was also verified by microstructural analysis. The observed defect-free microstructure of the squeeze cast composite is represented in Fig. 25 (a) and (b). The average hardness of composite manufactured by maintaining squeeze pressure of 46 MPa and 62 MPa revealed enhancement in hardness. This enhanced hardness was due to the squeeze pressure which tends to compact the matrix and thus results in a denser composite. The Differential Thermal Analysis (DTA) experiments revealed the presence of eutectic reaction (αAl + θ = Liquid). As shown in Fig. 25 (c) and (d), the presence of θ eutectic phase (Al_2_Cu) was also observed in microstructure and X-ray diffraction analysis. X-ray diffraction analysis revealed the formation of AlB_2_ from the decomposition of AlB_12_. This decomposition of AlB_12_ and formation of AlB_2_ along with defect free microstructure ultimately enhances the resulting mechanical properties of the squeeze cast composite.

**Fig. 26:**
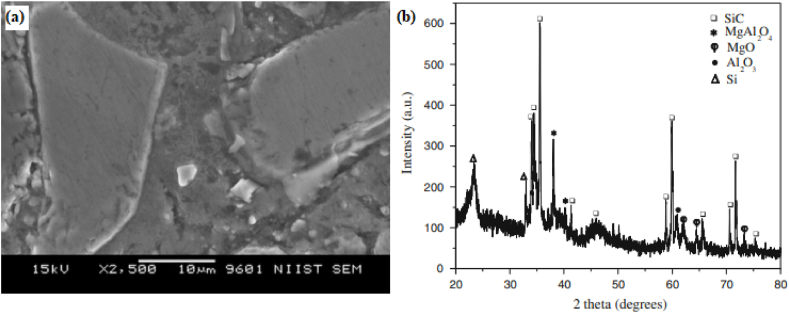
(a) Scanning electron microscopy and (b) X-ray diffraction pattern of AA 6061 + SiC composite [276].

Manu et al. [276] observed that characteristics of AA 6061-SiC composite were influenced by mould temperature, liquid metal superheat, squeezing pressure and rate of application of squeeze pressure. While examining the microstructure, gradient dispersion of the reinforcement particles in the molten matrix was observed. The scanning electron microscope image shown in Fig. 26(a) revealed good interfacial bonding between matrix and reinforcement. Proper infiltration along with the presence of aluminium binder results in the formation of good interfacial bonding between the matrix and the reinforcement particles. The plot of the X-ray diffraction pattern shown in Fig. 26(b), revealed the highest peak corresponding to SiC. Apart from this, a few peaks for the MgAl_2_O_4_ and MgO phases can also be observed. It is a known fact that magnesium is a powerful surfactant which extracts oxygen from molten metal and results in the formation of MgAl_2_O_4_ and MgO. The presence of MgAl_2_O_4_ and MgO improves the wettability and thus results in good bonding between the matrix and the reinforcement particles. It should be noted that the proportion of magnesium as a wetting agent should be restricted to 4.7%, as the addition of magnesium higher than 4.7% will result in the formation of brittle intermetallic compound Al_3_Mg_2_. Also, the X-ray diffraction pattern didn't reveal any peak corresponding to Al_4_C_3_. This Al_4_C_3_ is an unwanted precipitate which is brittle in nature and thus ultimately tends to degrade the mechanical properties of the resulting composite.

Xue et al. [277] performed an exothermic reaction between molten aluminium and salts of K_2_TiF_6_ and KBF_4_ and manufactured aluminium alloy 2014 reinforced with 5% TiB_2_ in-situ composite. By varying the squeeze pressure, variation in the microstructure and the mechanical properties of manufactured composites were investigated. For obtaining squeeze cast composites having maximum mechanical properties, pouring temperature and die temperature was maintained at 710 °C and 200 °C respectively whereas, squeeze pressure was maintained at 90 MPa. However, these parameters were increased to 780 °C, 250 °C and 120 MPa respectively, for manufacturing composites using the squeeze casting process and introducing TiB_2_ particles by the in-situ method. The in-situ formed TiB_2_ particle affects the solidification process, plasticity and fluidity of the composite and due to the same, higher pouring temperature, die temperature and squeeze pressure were required. By combining squeeze casting along with the in-situ method, refinement in microstructure was observed. The pressure applied during squeeze casting tends to eliminate the casting defects, gas porosity and thus improves the distribution of reinforcement particles. Due to the same, tensile strength was improved by 21%, yield strength was improved by 16% and elongation was found to increase by 200%. Lo et al. [278] used the numerical modelling approach to understand the relation between pre-form cracking and squeeze infiltration condition of magnesium composites. They reported that improper processing temperature can lead to preform cracks and deformation. Thus, it was suggested to maintain the uniform temperature in molten magnesium for manufacturing magnesium based squeeze cast composites. In the same line, Sampath et al. [268] developed an analytical model of porous fiber preform manufactured by liquid metal in the squeeze casting process. While modelling they assumed that the squeeze casting process was adiabatic and the flow during the process was unidirectional. It should be noted that the developed model didn't consider the thermal behaviour of the composites but it indeed studies the behaviour of the liquid metal and the fiber perform separately. Along with this, some simple equations were derived for calculating the process parameters like total time for completion and time for solidification. By examining the infiltration characteristics, the liquid superheat temperature, the pre-form preheat temperature and the squeeze pressure were also analyzed. Patel et al. [279] on the basis of the design of experiments performed several experiments by varying the squeeze pressure, the pouring temperature, the duration of applied pressure and the temperature of the die. The resulting outputs such as surface roughness, tensile strength and yield strength were analyzed by using the response surface methodology. Two nonlinear models were developed and the results of both models were compared with the experiments for validation purposes. It was reported that both developed models were statically adequate and were found to predict the results accurately.

The squeeze casting process is a potential casting process which finds application in several industries. Squeeze casting finds its niche in manufacturing safety and critical parts in automobile industries such as space frame joints [280]. Apart from this, squeeze casting finds its application in the infiltration of reinforced-ceramic fiber pistons for diesel engines. Popular automobile brand Porsche uses a squeeze casting process for manufacturing cylinder block banks for the horizontal V6 engine [281]. Porsche's Boxter engine having integrated AMC cylinder liner is also manufactured using a squeeze casting process [282]. Also, squeeze casting finds its application in manufacturing steering knuckles for low-volume vehicle lines.

### 3.4 In-situ method

Another method using which bulk composites can be manufactured is the in-situ method. In this process, the reaction between salts and the matrix results in the formation of secondary phase particles within the matrix itself. The manufactured composites will be characterized by an extremely fine and stable phase of reinforcement in the base alloy [283]. Zhang et al. [284] and Hsu et al. [285] reported that composites manufactured using the in-situ method offer various advantages over the ex-situ method such as higher wettability, improved bonding strength and compatibility between matrix and reinforcement. However, Birol [286] along with Tong and Fang [287] reported that in-situ composites suffer from a major drawback in which the reinforcement particles tend to segregate along the grain boundaries. To reduce agglomeration of these reinforcement particles several approaches such as extrusion, rolling, holding composite at a higher temperature than the melting point and many more have been adopted.

**Fig. 27:**
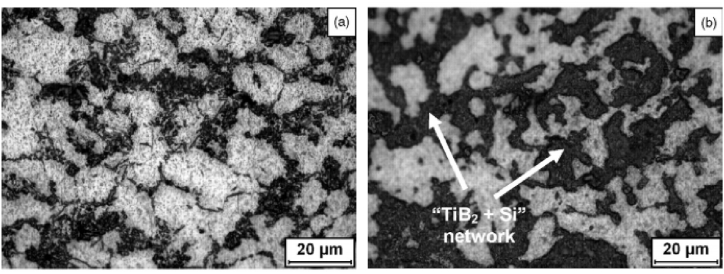
Image of optical microscopy (a) A356 + 5 wt% TiB_2_ and (b) A356 + 10 wt% TiB_2_ [289].

The pioneer in the manufacturing of aluminium based in-situ composites was done by Davies et al. [288]. The synthesis of Al–TiB_2_ was done by introducing salts of K_2_TiF_6_ and KBF_4_ in the molten matrix. The exothermic reaction between the molten matrix and salts results in the formation of an Al–TiB_2_ composite. Mandal et al. [289] manufactured A356-TiB_2_ composite using the in-situ method. X-ray diffraction pattern showed peaks of TiB_2_ particles only for composites having a higher weight percentage of reinforcement particles. Along with this, the X-ray diffraction pattern didn't reveal any peak corresponding to brittle Al_3_Ti particles. From Fig. 27 (a) – (b), it can be observed that an increase in the concentration of TiB_2_ particles tends to increase the agglomeration in the matrix. Also, it was observed that the increase in the concentration of TiB_2_ particles restricts the growth and dispersal of Si particles in the dendritic regions. It was observed that the presence of TiB_2_ particles significantly accelerates the aging kinetics. A higher weight fraction of TiB_2_ was found to narrow down the size of Si particles. This reduction in the size of Si particles contributes towards enhancement in the tensile strength of the manufactured composites. Sun et al. [290] manufactured Ti_2_(Al,Si)C reinforced Al composites in-situ composite and observed similar results. Aging time was found to reduce from 12 h for the base alloy to 4 h for manufactured composite. Post aging, the finer size of Si particles was found to a have round shape and these thermal modifications in Si particles were found to be beneficial for tensile strength and ductility of composite.

Lakshmi et al. [291] investigated the effect of reaction time on growth behaviour and weight percentage of TiB_2_ particles. The in-situ process was carried out at a constant reaction temperature of 850 °C and varying reaction time from 10 min to 40 min (in an interval of 10 min). It was observed that with the increase in reaction time up to 20 min, the concentration/amount of TiB_2_ particles increase. However, a further increase in the reaction time was found to have an adverse effect on the concentration of TiB_2_ particles. The variation in concentration of TiB_2_ particles with the change in reaction time was attributed to the increase in the volume of cryolite slag as the reaction time increases. Thus, it can be said that for the reaction time of 20 min, the rate of formation of TiB_2_ particles will be higher than the volume of cryolite slag. Optical microscopy revealed that TiB_2_ particles were distributed along the grain boundaries and minimum agglomeration was reported. The presence of TiB_2_ particles along the grain boundaries indicates that particles get segregated at the solid-liquid interface during solidification. Scanning electron micrographs for specimens manufactured by maintaining a reaction time of 10 min revealed the hexagonal shape of TiB_2_ particles. Also, a lack of reaction time was reported, as scanning electron micrographs showed the presence of residual salts which didn't undergo an exothermic chemical reaction. While increasing reaction time from 10 min to 30 min, a reduction in the grain size of the composite was reported. However, a further increase in reaction time was found to have an adverse effect on grain size. Charbhai et al. [292] and Nandam et al. [293] also manufactured Al/TiB_2_ composites by reacting the same salts in the molten phase of the matrix. Degradation in mechanical properties was observed due to the formation of the Al_3_Ti phase in the resulting in-situ composite. Zhang et al. [294] reported that the Al_3_Ti phase is brittle in nature and thus the formation of this phase ultimately increases the brittleness of manufactured composites. However, Rajan et al. [295] and Wang et al. [296] suggested that the formation of the Al_3_Ti phase can be avoided by several ways such as increasing the reaction time between salt and molten matrix, increasing temperature and controlling the fraction of salts. Mandal et al. [289] performed an X-ray diffraction analysis and observed that the Al_3_Ti phase was absent in in-situ Al/TiB_2_. It was also reported that maintaining 800 °C as melt temperature, reaction time between salts and molten matrix for 1 h and intermediate stirring at every 10 min avoids the formation of the Al_3_Ti phase.

**Fig. 28:**
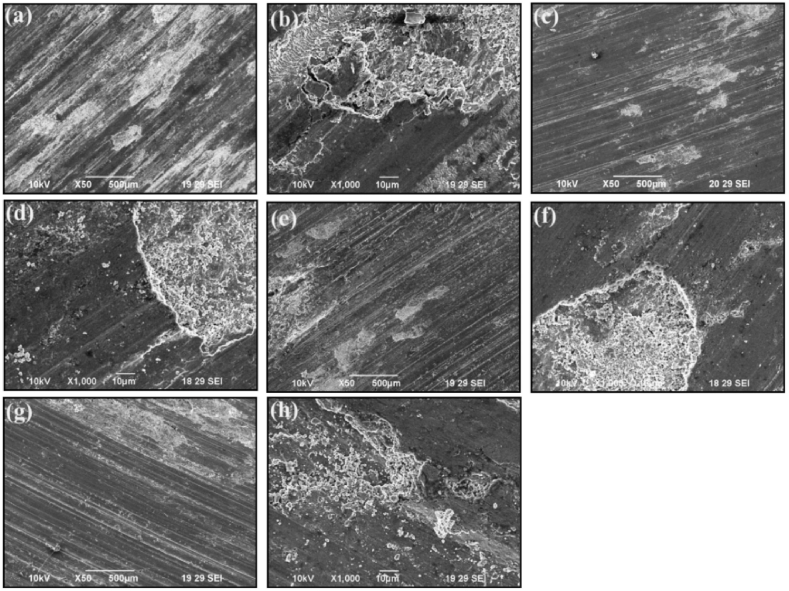
Micrographs of worn surfaces of AA 7075 + TiB_2_ in-situ composites (a, b) 0 wt%, (c, d) 3 wt%, (e, f) 6 wt% and (g, h) 9 wt% [295].

Bannan et al. [297] manufactured TiC reinforced copper and aluminium bronze composites. The carbon monoxide was generated as a by-product of the heat of the reaction between the induction field, graphite crucible and crucible lid. This carbon monoxide maintains an inert atmosphere for in-situ reactions to take place. At a temperature of 1250 °C aluminium bronze reinforced with TiC having a particle size in the range of 1–3 μm was obtained. Whereas, copper reinforced with TiC having a particle size of 1–6 μm requires a higher temperature of 1330 °C. The dispersion concentration of TiC in copper based composite and aluminium based composite was 20% and 6.5% respectively. Results of scanning electron microscopy reported the presence of iron in aluminium bronze which ultimately improves the dispersion of TiC in aluminium bronze. Balaji et al. [298] used the spark plasma sintering technique for manufacturing TiB/TiC in situ composites and investigated tribological properties. Results showed that increasing TiB and TiC particulates in the titanium matrix enhances the hardness and wear resistance. At the same time, the presence of Fe-rich debris serves as a solid lubricant and improves the wear resistance of the manufactured in-situ composite. Rajan et al. [295] examined the effect of TiB_2_ content on the morphology of worn surfaces. As represented in Fig. 28, the worn surfaces revealed the presence of parallel grooves which clearly indicates abrasive wear mode. Also, it was reported that with the increase in the content of TiB_2_, the depth of grooves and plastic deformation at the edges of grooves decreases. Apart from this, the cutting marks observed in Fig. 28 (a) and (b) were absent on the worn surface of the composite material (refer to Fig. 28(c)–(h)). This indeed indicates that the presence of TiB_2_ particles resists the cutting action and reduces wear rate.

Khorasania et al. [299] manufactured Mg–Al_2_Ca–Mg_2_Ca in-situ composites by considering various ratios of Ca/Al (Mg–17Al–8Ca, Mg–14Al–11Ca and Mg-12.5Al-12.5Ca) and investigated the microstructure and tensile properties. Two different conditions were focused i.e. as-cast and extruded. It was reported that increasing the ratio of Ca/Al slightly improves the tensile properties of as-cast composites. However, significant improvement in tensile properties was observed after the extrusion process. The formation of finely distributed hard (Al_2_Cu) and ductile (Mg_2_Ca) phases tends to improve the toughness. Herbert et al. [300] investigated the tensile properties of casted Al/4.5% Cu alloy and mushy state rolled Al + 4.5% Cu + 5% TiB_2_ in-situ composite. It was reported that mushy state rolled composite was having enhanced mechanical properties compared to cast alloy. Similarly, several researchers observed enhancement in mechanical properties by performing several post casting processes such as cold rolling, cold drawing, hot rolling process and hot pressing [[301], [302], [303], [304]].

Kumar et al. [305] investigate the wear property of manufactured composites at various loads and constant sliding speeds. The obtained results of the wear test were as per Archard's Law. At the same time, with the increase in the concentration of TiC particles, wear rate and friction coefficient was found to decrease. This reduction in wear rate was attributed to (i) higher hardness of TiC particles and (ii) the formation of a well compacted mechanically mixed layer of wear debris on the worn surface. Lee et al. [306] manufactured copper-chromium in-situ composite and investigated the thermomechanical behaviour of the resulting composite. The mechanical behaviour of the composite was investigated at three distinct temperatures i.e. cryogenic temperature, room temperature and 400 °C. Extensive damage in terms of reinforcement failure and cavitation was observed when the tensile test and isothermal creep were performed at cryogenic temperature and 400 °C temperature, whereas comparatively lower damage was observed at room temperature. Madhavan et al. [307] manufactured Al/TiB_2_ in-situ composite and examined the mechanism involved in failure along with deformation behaviour. Particle size and holding time were the dominating parameters. Major failure was observed due to the yielding of the matrix and this yielding was commenced due to slip lines. While examining the dimpled structure of the composite, micro-void coalescence failure was observed. Failure mechanism due to compression was found to have micro-buckling followed by particle failure and specimens were found to have splitting or splaying mode failure.

Chen et al. [308] in their recent article manufactured copper based hybrid composites reinforced with particles of TiB_2_ and Al_2_O_3_. These hybrid composites were manufactured using liquid based in-situ casting technique and the manufactured composites were processed by hot and cold rolling. It was reported that combining liquid phase in-situ casting technique with large deformation of around 90% enhances the mechanical properties of the hybrid composites. The large deformation generated by hot and cold rolling process leads to fill the casting defects, refines the grain structure and break the clusters of reinforcement particles. Zhang et al. [309] combined gel-casting with in-situ casting and manufactured porous materials. It was reported that this combination can be adopted for manufacturing porous composites materials which has both enhanced mechanical properties along with electromagnetic wave transparent properties. This porous silicon nitride ceramic composites can be used for bone substitute applications in medical sector. Xie et al. [310] used ultrasonic assisted in-situ casting technique for manufacturing AA 2195 reinforced with 2% of TiB_2_. It was reported that ultrasound further refines grain and prevent agglomeration of reinforcement particles. Furthermore, ultrasound enhances both the strength and ductility of the manufactured composites.

Similar to stir casting, the in-situ process is still under the research phase. Due to the same, no industrial application of the in-situ casting process exists. Several researchers and academicians are attempting to manufacture various composites using an in-situ process and the observed results are represented in Table 5. The data represented in Table 5 may prove to be helpful to researchers in the selection of the controlling parameters.

**Table 5:** Range/magnitude of process parameters, tensile strength and hardness of composites manufactured by combination of squeeze casting, stir casting and in-situ casting technique.

Sr. No.	Composite	Processing Technique	Process parameter and their range or magnitude	Tensile Strength (MPa)	Hardness	Reference
1	AA 7075 + h-BN/NiTi	Squeeze Casting + Stir Casting	Melting Temperature: 750 °C Preheating Temperature: 500 °C Stirring Speed: 350 rpm Squeezing Pressure: 400 MPa In presence of Argon gas, Ultrasonic wave introduced in molten mixture at 18 kHz for 10 min	–	–	[311]
2	Aluminium + Al_2_O_3_	Squeeze Casting + Stir Casting	Squeeze Pressure: 100 MPa Squeeze Time: 30 s Die Pre Heating Temperature: 350 °C Stirring Speed 450 rpm	152 MPa	45.55 HRB	[312]
3	Aluminium + Al_2_O_3_	Squeeze Casting + Stir Casting	Squeeze Pressure: 100 MPa Squeeze Time: 45 s Die Pre Heating Temperature: 250 °C Stirring Speed 525 rpm	151.7 MPa	60.9 HRB	[312]
4	AA 2014 + 5 vol % TiB_2_	In-Situ + Squeeze Casting	Salts: K_2_TiF_6_, KBF_4_ Exothermic Reaction Temperature: 850 °C Pouring Temperature: 710, 750 and 780 °C Die Temperature: 200 and 250 °C Squeezing Pressure: 0, 60, 90, 120 MPa	401 MPa (for 0 MPa Pressure) 452 MPa (for 60 MPa Pressure) 480 MPa (for 90 MPa Pressure) 487 MPa (for 120 MPa Pressure)	160 HV (for 0 MPa Pressure) 165 HV (for 60 MPa Pressure) 168 HV (for 90 MPa Pressure) 171 HV (for 120 MPa Pressure)	[277]
5	LM 24 + 7.5 wt % SiC +2.5 wt %Fly Ash	Squeeze Casting + Stir Casting	Melting temperature: 600 °C Particle Preheating Temperature: 400 °C Stirring Time: 5 min Die Preheating Temperature: 200 °C Squeezing Pressure: 125 MPa Squeezing Time: 60 s Mould Temperature: 300 °C Pouring Temperature: 725 °C	385 MPa	–	[313]
6	Al–Cu–Mg alloy + 40% Al_2_O_3_ (fiber) + 40% Al_2_O_3_ (particles)	Squeezing Casting	Binding Agent: Polyvinyl Alcohol Melting Temperature of Matrix: 1073 K Preform Temperature: 973 K Mould Temperature: 573 K Squeezing Pressure: 40 MPa Squeezing Time: 60 Seconds	290 MPa	–	[314]
7	A356 + 10 vol % SiC	Squeeze Casting + Stir Casting	Melting Temperature: 750, 800, 850 and 900 °C Particle Preheating Temperature: 1000 °C Particle Preheating Time: 02 Hours Stirring Time: 10 min Die Temperature: 400 °C Squeezing Pressure: 100 MPa	174 MPa (for melting temperature of 850 °C)	89.22 HB (for melting temperature of 850 °C)	[315]
8	A356 + 10 vol % SiC	Squeeze Casting + Stir Casting	Melting Temperature: 800 °C Particle Preheating Temperature: 1000 °C Particle Preheating Time: 02 Hours Stirring Time: 10 min Die Temperature: 250, 300, 350 and 400 °C Squeezing Pressure: 100 MPa	245.75 MPa (for die temperature of 350 °C)	91.90 HB (for die temperature of 350 °C)	[315]
9	AA 7075 + 5% TiB_2_ AA 7075 + 7.5% TiB_2_	In-situ casting + Stir Casting	Melting Temperature: 750 °C Degassing Agent: C_2_Cl_6_ Slats: K_2_TiF_6_, KBF_4_ and Halide Stirring Time: 10 min Reaction Time: 01 Hour	218 MPa 225 MPa	78 HV 88 HV	[316]

## 4 Comparison of manufacturing technique

To achieve desired characteristics of the composites, the manufacturing process plays a crucial role. Thus, it becomes necessary to compare various manufacturing techniques discusses in the aforementioned sections. The manufacturing techniques have been compared based on the criteria mentioned in Table 6. The data presented in Table 6 will be helpful for industries to select the appropriate technique for manufacturing composite materials.

**Table 6:** Comparison of various techniques using which MMC can be manufactured.

Sr. No.	Criteria	Solid State Processing		Liquid State Processing			
		Friction Stir Processing	Powder Metallurgy	Centrifugal Casting	Stir Casting	Squeeze Casting	In-Situ Technique
1	Capability of manufacturing composites	Surface composites	Bulk Composites Functionally Graded Composites (FGM) Composites having step-wise structure	Functionally Graded Composites	Bulk Composites	Bulk Composites Functionally Graded Composites	Bulk Composites
2	Shape of resulting composites	Plates	Depends upon the die and punch, possible to manufacture complex shape	Hollow shape	Depends upon the mould, possible to manufacture complex shape	Depends upon the die and punch, possible to manufacture complex shape	Depends upon the mould, possible to manufacture complex shape
3	Requirement of major equipment	Vertical milling machine & fixture for holding workpiece Friction Stir Welding Setup	Melt atomization or Ball miller (for powder preparation) Rotating Drum or double cone screw mixer blade mixture (for mixing powders) Hydraulic Press (for compacting) Furnace (for sintering)	Furnace (for preheating particles and melting matrix) Rotating Mould (for generating centrifugal force)	Furnace (for preheating particles and melting matrix) Mechanical Stirrer (for mixing molten mixture) Mould (for getting required shapes)	Furnace (for preheating particles and melting matrix) Hydraulic Press (for compacting) Die and Punch (for manufacturing complex shapes)	Furnace (for preheating particles and melting matrix) Mould (for getting required shapes)
4	Types of defects that manufactured composites may have	Cracks, pores, voids and tunnel, fragment, lack of penetration, kissing bond, hooking, flash, and other surface defects	Ejection cracks, density variations, micro-laminations, and poor sintering	Gas porosity, shrinkage defects, mould material defects, pouring metal defects, cracking/tearing around circumferences and metallurgical defects	Gas porosity, shrinkage defects, mould material defects, pouring metal defects, cracking/tearing around circumferences and metallurgical defects	Gas porosity, shrinkage defects, mould material defects, pouring metal defects, cracking/tearing around circumferences and metallurgical defects	Gas porosity, shrinkage defects, mould material defects, pouring metal defects, cracking/tearing around circumferences and metallurgical defects
5	Level of defects	Microscopic and sometimes Macroscopic	Microscopic and sometimes Macroscopic	Both Microscopic and Macroscopic	Both Microscopic and Macroscopic	Both Microscopic and Macroscopic	Both Microscopic and Macroscopic
6	Frequency of defects in manufactured composites	Moderate	Low	High	High	Moderate	High
7	Controlling content of reinforcement particles	Difficult to control weight or volume percent of reinforcement	Easier to control weight percent of reinforcement particles	Easier to control both weight and volume percent of reinforcement particles	Easier to control both weight and volume percent of reinforcement particles	Difficult to control weight or volume percent of reinforcement	Difficult to control weight or volume percent of reinforcement
8	Distribution of reinforcement particles	Homogenous distribution	Homogenous, Step wise distribution, Varying distribution along the thickness	Varying distribution along the thickness	Homogenous distribution	Heterogeneous or Homogenous distribution	Heterogeneous or Homogenous distribution
9	Equipment and production cost	Moderate	High	Low	Low	Moderate	Low
10	Highlighting Feature	Green manufacturing technique for altering the microstructure	Ability to combine materials in powder form that are otherwise immiscible	Composites can be made in almost any length, thickness and diameter	Simplicity, flexibility and applicability to large quantity production with cost advantage	Minimize both solidification shrinkage and gas compression	Cheapest method, suitable for large scale production
11	Limitation	Low production rate, lesser flexible, cannot process non-forgeable materials	Intricate designs cannot be made, economical only for mass production	Requires skilled labour, difficult to control internal diameter of composites, limited strength of cast composites	Thermal mismatch, poor wettability, possibility of interfacial reaction, requires post processing techniques to resolve agglomeration and casting defects	Low flexible, high cycle time, difficult to maintain homogeneity and higher possibility of reaction between matrix and reinforcement	Composites with higher content of reinforcement particles cannot be manufactured

## 5 Summary, critical analysis, shortfall and future scope

The present article attempts to review the literature available in the area of manufacturing MMC using different techniques. Major solid state processing techniques and liquid state processing techniques have been assessed in the present article and critical comments have been made. The subsequent section presents the summary, critical analysis and shortfall of individual processes.

### 5.1 Friction stir processing

Friction stir processing is an innovative technique that distributes secondary phase particles homogeneously in the matrix phase. This processing technique is only capable of manufacturing surface composites. However, this technique leads to grain refinement with the improvement in the mechanical properties and avoids the interfacial reaction between matrix and reinforcement particles. Among several process parameters, groove width, number of passes, traverse speed and rotational speed are the dominating parameters. However, the application of friction stir processing is restricted as bulk composites and gradient distribution of reinforcement particles is not possible using this process. Furthermore, the implementation of friction stir processing requires extensive understating related to the selection of process parameters and groove dimensions. Existing literature shows a lack of study related to optimization of process parameters and thus creates scope for further perusal.

### 5.2 Powder metallurgy

Powder metallurgy is capable of manufacturing bulk composites or functionally graded composites having stepwise configurations. Composites manufactured using powder metallurgy tends to have superior quality of microstructure and better mechanical properties. Dominating process parameters governing the quality of the resulting composite are sintering temperature; sintering time and compact pressure. There exist pre-processing techniques, post-processing techniques and certain derivatives which are associated with powder metallurgy. Powder metallurgy when combined with the in-situ method, forging method, flake powder metallurgy and vibration of the mixture will further enhance several characteristics of the bulk composite. However, it becomes necessary to select the appropriate powder size for matrix and reinforcement particles. This indeed requires an ample amount of research and thus creates scope for future study.

### 5.3 Centrifugal casting

Composites having different weight percent of reinforcement particles on the inner zone and external zone can be manufactured using centrifugal casting. Due to thermal mismatch, the inner zone will tend to have tensile residual stress whereas, the external zone will have compressive residual stress. The pouring temperature of the molten matrix, the rotational speed of mould and the velocity of reinforcement particles in the molten matrix are the dominating parameters affecting the distribution of reinforcement particles. However, a lack of interfacial bonding between matrix and reinforcement particles was observed. Due to the same, reinforcement particles were either embedded in the matrix or were agglomerated in several zones of the matrix. Thus, the issue related to the lack of interfacial bonding between matrix and reinforcement particles needs to address so that agglomeration of reinforcement particles can be reduced or avoided. At the same time, it requires a deep understanding of various process parameters and control over certain parameters became difficult. There is a need for research related to the determination of temperature and the solidification process.

### 5.4 Stir casting

The stir casting method is capable of manufacturing composites having complex shapes at a comparatively cheaper cost. Stirring speed, stirring time and stirrer design need to select wisely as these parameters govern the distribution of reinforcement particles. From the state-of-the-art of literature, it can be observed that several aspects such as wettability between molten alloy and reinforcement particles, secondary phase particles distribution, defects, precautions for avoiding slag formation, reducing casting defects and chemical reaction require further investigation. Existing solutions related to improvement in wettability are restricted to certain grades of matrix and reinforcement particles. Also, there exists an extensive need for research in the area of optimization of several process parameters. Despite of several post processing techniques such as hot/cold rolling and drawing, the microstructure of resulting composites still has some agglomeration of reinforcement particles along with the casting defects. Thus, the aforementioned issues related to the distribution of reinforcement particles and defects need special attention.

### 5.5 Squeeze casting

Squeeze casting results in the gradient distribution of reinforcement particles in the matrix. Dominating parameters which affect the properties of manufactured composites are mould temperature, liquid metal superheat, squeezing pressure and rate of application of squeeze pressure. Apart from this, it can be said that forging action is the major advantage of this method. However, the diversity in the refinement of reinforcement particles at the bottom and top surface needs special attention and thus, lays the foundation for the scope of research. Along with this, squeeze casting offers less flexibility in part geometry, lower productivity, higher equipment cost and larger initial capital. It should also be noted that the flow of molten metal in the die should be laminar which is challenging to attain. Lastly, squeeze casting combined with the in-situ method tends to improve mechanical properties. Also, this combined process results in grain refinement, reduction in casting defects and improvement in the dispersion of reinforcement particles.

### 5.6 In-situ technique

Regarding the in-situ technique, it can be observed that the majority of the researchers have manufactured Al/TiC in-situ composites by introducing salts of K_2_TiF_6_ and KBF_4_ in the molten aluminium. Comparatively, there exists less literature which focuses on the manufacturing of composites other than Al/TiC. It also requires extensive research regarding the selection of several salts such that the reaction between those salts and molten metal results in the formation of a specific composite. A detailed study regarding reaction time, melting temperature, holding temperature and holding time is also missing in the existing literature. Apart from this, the in-situ method fails to manufacture composites reinforced with higher weight/volume percent of reinforcement particles. Besides this, to achieve enhanced mechanical properties it becomes necessary to adopt several post processing techniques.

## Author contribution statement

All authors listed have significantly contributed to the development and the writing of this article.

## Funding statement

This research did not receive any specific grant from funding agencies in the public, commercial, or not-for-profit sectors.

## Data availability statement

No data was used for the research described in the article.

## Declaration of interest’s statement

The authors declare no conflict of interest.

## References

[1] Aleksendric D., Carlone P. (2015).

[2] Tokaji K. (2005). Effect of stress ratio on fatigue behaviour in SiC particulate-reinforced aluminum alloy composites. Fatig. Fract. Eng. Mater. Struct..

[3] Yang Y., Boom R., Irion B., Heerdern D., Kuiper P., Wit H. (2012). Recycling of composites materials. Chem. Eng. Process: Process Intensif..

[4] Pandya D., Badgujar A., Ghetiya N. (2021). A Novel perception towards welding of Stainless Steel by Activated TIG welding: a review. Mater. Manuf. Process..

[5] Garshin A.P., Kulik V.I., Nilov A.S. (2018). Main areas for improving refractory fiber-reinforced ceramic matrix composite corrosion and heat resistance (review). Refract. Ind. Ceram..

[6] Tan S.J., Zeng X.X., Ma Q., Wu X.W., Guo Y.G. (2018). Recent advancements in polymer-based composite electrolytes for rechargeable lithium batteries. Electrochem. Energy Rev..

[7] Adalarasan R., Shanmuga P.C., Arunachalam R., Sudhir R. (2011). An evaluation of mechanical properties and microstructure of dispersion strengthened Al-6063 obtained by in-situ fabrication. Int. J. Des. Manuf. Technol..

[8] Parikh V.K., Badgujar A.D., Ghetiya N.D. (2019). Joining of metal matrix composites using friction stir welding: a review. Mater. Manuf. Process..

[9] Chawla K.K. (1998).

[10] Chawla N., Chawla K.K. (2006).

[11] Sahraeinejad S., Izadi H., Haghshenas M., Gerlich A.P. (2015). Fabrication of metal matrix composites by friction stir processing with different Particles and processing parameters. Mater. Sci. Eng., A.

[12] Ibrahim M.F., Ammar H.R., Alkahtani S.A., Samuel F.H. (2016). Metallographic investigation of tensile and impact tested aluminum composites. J. Compos. Mater..

[13] Parikh V.K., Badheka V.J., Badgujar A.D., Ghetiya N.D. (2021). Fabrication and processing of aluminium alloy metal matrix composites. Mater. Manuf. Process..

[14] Naseer A., Ahmad F., Aslam M., Guan B.H., Harun W.S.W., Muhamad N., Raza M.R., German R.M. (2019). A review of processing techniques for graphene-reinforced metal matrix composites. Mater. Manuf. Process..

[15] Lopez V.H., Scoles A., Kennedy A.R. (2003). The thermal stability of TiC particles in an A17 wt.% Si alloy. Mater. Sci. Eng..

[16] Bandyopadhyay N.R., Ghosh S., Basumallick A. (2007). New generation metal matrix composites. Mater. Manuf. Process..

[17] Sankhla A., Patel K.M. (2022). Metal matrix composites fabricated by stir casting process – a review. Adv. Mater. Process. Techn..

[18] Panwar N., Chauhan A. (2018). Fabrication methods of particulate reinforced Aluminium metal matrix composite - a review. Mater. Today Proc..

[19] Khan K.B., Kutty T.R.G., Surappa M.K. (2006). Hot hardness and indentation creep study on Al- 5% Mg alloy matrix-B4C particle reinforced composites. Mater. Sci. Eng..

[20] Jayalakshmi S., Kailas S.V., Seshan S., Kumar K., Srivatsan T.S. (2005). Damage tolerant magnesium metal matrix composites: influence of reinforcement and processing. Mater. Manuf. Process..

[21] Taha M.A. (2001). Industrialization of cast aluminum matrix composites (AMCCs). Mater. Manuf. Process..

[22] Taha M.A., El-Mahallawy N.A., El-Sabbagh A.M. (2008). Some experimental data on workability of aluminium-particulate-reinforced metal matrix composites. J. Mater. Process. Technol..

[23] Padhy G.K., Wu C.S., Gao S. (2018). Friction stir based welding and processing technologies - processes, parameters, microstructures and applications: a review. J. Mater. Sci. Technol..

[24] Sharma V., Prakash U., Manoj B.V. (2015). Surface composites by friction stir processing: a review. J. Mater. Process. Technol..

[25] Arifi A., Sulong A.B., Muhamad N., Syarif J., Ramli M.I. (2014). Material processing of hydroxyapatite and titanium alloy (HA/Ti) composite as implant materials using powder metallurgy: a review. Mater. Des..

[26] Bains P.S., Sidhu S.S., Payal H.S. (2016). Fabrication and machining of metal matrix composites: a review. Mater. Manuf. Process..

[27] Dhandapani S., Rajmohan T., Palanikumar K., Charan M. (2016). Synthesis and characterization of dual particle (MWCT þ B4C) reinforced sintered hybrid aluminum matrix composites. Part. Sci. Technol..

[28] Kandpal B.C., Kumar J., Singh H. (2018). Manufacturing and technological challenges in Stir casting of metal matrix composites-A Review. Mater. Today Proc..

[29] Annigeri U.K., Kumar G.B.V. (2017). Method of stir casting of Aluminum metal matrix Composites: a review. Mater. Today Proc..

[30] Yigezu B.S., Jha P., Mahapatra M. (2013). The key attributes of synthesizing ceramic particulate reinforced Al-based matrix composites through stir casting process: a review. Mater. Manuf. Process..

[31] Arunachalam R., Krishnan K., Muraliraja R. (2019). A review on the production of metal matrix composites through stir casting-furnace design, properties, challenges and research opportunities. J. Manuf. Process..

[32] Naebe M., Shirvanimoghaddam K. (2016). Functionally graded materials: a review of fabrication and properties. Appl. Mater. Today.

[33] Page M.J. (2021). The PRISMA 2020 statement: an updated guideline for reporting systematic reviews. Syst. Rev..

[34] Moher D., Liberati A., Tetzlaff J., Altman D.G., PRISMA Group (2009). Preferred reporting items for systematic reviews and meta-analyses: the PRISMA statement. Ann. Intern. Med..

[35] Thomas W, Nicholas E, Needham J, Murch M, Temple-Smith P, Dawes C. Friction stir butt welding. International patent No. PCT/GB92/02203, GB Patent No. 9125978.8. U.S.Patent No. 5,460,317.

[36] Mishra R.S., Ma Z.Y., Charit I. (2003). Friction stir processing: a novel technique for fabrication of surface composite. Mater. Sci. Eng..

[37] Heidarzadeh A., Mironov S., Kaibyshev R., Çam G., Simar A., Gerlich A., Khodabakhshi F., Mostafaei A., Field D.P., Robson J.D., Deschamps A., Withers P.J. (2021). Friction stir welding/processing of metals and alloys: a comprehensive review on microstructural evolution. Prog. Mater. Sci..

[38] Liu S., Paidar M., Mehrez S., Ojo O.O., Cooke K.O., Wang Y. (2022). Fabrication of AA6061/316 composites via a double pin FSP tool. J. Mater. Res. Technol..

[39] Luo J., Liu S., Paidar M., Vignesh R.V., Mehrez S. (2022). Enhanced mechanical and tribological properties of AA6061/CeO2 composite fabricated by friction stir processing. Mater. Lett..

[40] Parikh V.K., Badgujar A.D., Ghetiya N.D. (2022). Investigation of microstructural and wear properties of stir cast and friction stir processed AA 2014-based metal matrix composites. Adv. Mater. Process. Techn..

[41] Mishra R.S., Mahoney M.W., McFadden S.X., Mara N.A., Mukherjee A.K. (2000). High strain rate superplasticity in a friction stir processed 7075 Al alloy. Scripta Mater..

[42] Ma Z.Y. (2008). Friction stir processing technology: a review. Metall. Mater. Trans..

[43] Rathee S., Maheshwari S., Siddiquee A.N., Srivastava M. (2017). Investigating effects of groove dimensions on microstructure and mechanical properties of AA6063/SiC surface composites produced by friction stir processing. Trans. Indian Inst. Met..

[44] Sunil B.R., Reddy G.P.K.R., Patle H., Dumpala R. (2016). Magnesium based surface metal matrix composites by friction stir processing. J. Magnes. Alloys.

[45] Devaraju A., Kumar A., Kumaraswamy A., Kotiveerachari B. (2013). Influence of reinforcements (SiC and Al2O3) and rotational speed on wear and mechanical properties of aluminum alloy 6061 – T6 based surface hybrid composites produce via friction stir processing. Mater. Des..

[46] Shafiei-Zarghani A., Kashani-Bozorg S.F., Zarei-Hanzaki A. (2011). Wear assessment of Al/Al2O3 nano-composite surface layer produced using friction stir processing. Wear.

[47] Avettand-Fènoël M.N., Simar A., Shabadi R., Taillard R., Meester B. (2014). Characterization of oxide dispersion strengthened copper based materials developed by friction stir processing. Mater. Des..

[48] Choi D.H., Kim Y.H., Ahn B.W., Kim Y.I., Jung S.B. (2013). Microstructure and mechanical property of A356 based composite by friction stir processing. Trans. Nonferrous Metals Soc. China.

[49] Bharti S., Ghetiya N.D., Patel K.M. (2020). Micro-hardness and wear behavior of AA2014/Al2O3 surface composite produced by friction stir processing. SN Appl. Sci..

[50] Jafari J., Givi M.K.B., Barmouz M. (2015). Mechanical and microstructural characterization of Cu/CNT nanocomposite layers fabricated via friction stir processing. Int. J. Adv. Manuf. Technol..

[51] Sagar P., Handa A. (2021). Selection of tool traverse speed considering trial run experimentations for AZ61/TiC composite developed via friction stir processing using triangular tool. Mater. Today Proc..

[52] Golmohammadi M., Atapour M., Ashrafi A. (2015). Fabrication and wear characterization of an A413/Ni surface metal matrix composite fabricated via friction stir processing. Mater. Des..

[53] Manochehrian A., Heidarpour A., Mazaheri Y., Ghasemi S. (2019). On the surface reinforcing of A356 aluminum alloy by nanolayered Ti3AlC2 MAX phase via friction stir processing. Surf. Coating. Technol..

[54] Tang J., Shen Y., Li J. (2019). Investigation of microstructure and mechanical properties of SiC/Al surface composites fabricated by friction stir processing. Mater. Res. Express.

[55] Tonelli L., Morri A., Toschi S., Shaaban M., Ammar A.R., Ahmed M.A.Z., Ramadan R.M., El-Mahallawi I., Ceschini L. (2019). Effect of FSP parameters and tool geometry on microstructure, hardness, and wear properties of AA7075 with and without reinforcing B4C ceramic particles. Int. J. Adv. Manuf. Technol..

[56] Gao J., Zhang S., Jin H., Shen Y. (2019). Fabrication of Al7075/PI composites base on FSW technology. Int. J. Adv. Manuf. Technol..

[57] Singh T., Tiwari S.K., Shukla D.K. (2019). Friction-stir welding of AA6061-T6: the effects of Al2O3 nano-particles addition. Res. Mater..

[58] Papantoniou I.G., Kyriakopoulou H.P., Pantelis D.I., Athanasiou-Ioannou A., Manolakos D.E. (2018). Manufacturing process of AA5083/nano-γAl2O3 localized composite metal foam fabricated by friction stir processing route (FSP) and microstructural characterization. J. Mater. Sci..

[59] Guo J.F., Liu J., Sun C.N., Maleksaeedi S., Bi G., Tan M.J., Wei J. (2014). Effects of nano-Al2O3 particle addition on grain structure evolution and mechanical behaviour of friction-stir-processed Al. Mater. Sci. Eng..

[60] Ahmoye D., Krstic V.D. (2015). Reaction sintering of SiC composites with in situ converted TiO2 to TiC. J. Mater. Sci..

[61] Liang X., Earthman J.C., Wolfenstine J., Lavernia E.J. (1992). A comparison of techniques for determining the volume fraction of particulates in metal matrix composites. Mater. Char..

[62] Fang C.K., Fang R.L., Weng W.P., Chuang T.H. (1999). Applicability of ultrasonic testing for the determination of volume fraction of particulates in alumina-reinforced aluminum matrix composites. Mater. Char..

[63] Sharma V., Tripathi P.K. (2022). Approaches to measure volume fraction of surface composites fabricated by friction stir processing: a review. Measurement.

[64] Sathiskumar R., Murugan N., Dinaharan I., Vijay S.J. (2013). Effect of traverse speed on microstructure and microhardness of Cu/B 4C surface composite produced by friction stir processing. Trans. Indian Inst. Met..

[65] Rathee S., Maheshwari S., Siddiquee A.N., Srivastava M.A. (2018). Review of recent progress in solid state fabrication of composites and functionally graded systems via friction stir processing. Crit. Rev. Solid State Mater. Sci..

[66] Tsai F.Y., Kao P.W. (2012). Improvement of mechanical properties of a cast Al-Si base alloy by friction stir processing. Mater. Lett..

[67] Mahmoud T.S., Mohamed S.S. (2012). Improvement of microstructural, mechanical and tribological characteristics of A413 cast Al alloys using friction stir processing. Mater. Sci. Eng. A.

[68] Karthikeyan L., Senthil Kumar V.S. (2011). Relationship between process parameters and mechanical properties of friction stir processed AA6063-T6 aluminum alloy. Mater. Des..

[69] Rajakumar S., Muralidharan C., Balasubramanian V. (2011). Influence of friction stir welding process and tool parameters on strength properties of AA7075-T6 aluminium alloy joints. Mater. Des..

[70] Çam G., Serindağ H.T., Çakan A., Mistikoglu S., Yavuz H. (2008). The effect of weld parameters on friction stir welding of brass plates. Mater. Sci. Engin. Techn..

[71] Rathee S., Maheshwari S., Noor Siddiquee A., Srivastava M., Kumar Sharma S. (2016). Process parameters optimization for enhanced microhardness of AA 6061/SiC surface composites fabricated via friction stir processing (FSP). Mater. Today Proc..

[72] Çam G., İpekoğlu G. (2017). Recent developments in joining of aluminum alloys. Int. J. Adv. Manuf. Technol..

[73] Cavaliere P., De Marco P.P. (2007). Friction stir processing of AM60B magnesium alloy sheets. Mater. Sci. Eng. A.

[74] Ma Z.Y., Mishra R.S. (2005). Development of ultrafine-grained microstructure and low temperature (0.48 Tm) superplasticity in friction stir processed Al-Mg-Zr. Scripta Mater..

[75] Mahoney M.W., Barnes A.J., Bingel W.H., Fuller C.B. (2004). Superplastic forming of 7475 Al sheet after friction stir processing (FSP). Mater. Sci. Forum.

[76] Palanivel R., Koshy Mathews P., Murugan N., Dinaharan I. (2012). Effect of tool rotational speed and pin profile on microstructure and tensile strength of dissimilar friction stir welded AA5083-H111 and AA6351-T6 Aluminum Alloys. Mater. Des..

[77] Elangovan K., Balasubramanian V. (2008). Influences of tool pin profile and tool shoulder diameter on the formation of friction stir processing zone in AA6061 aluminium alloy. Mater. Des..

[78] Elangovan K., Balasubramanian V., Valliappan M. (2008). Effect of tool pin profile and tool rotational speed on mechanical properties of friction stir welded AA6061 aluminium alloy. Mater. Manuf. Process..

[79] Zhang M., Paidar M., Ojo O.O., Narayanasamy S.M.S., Zain A.M., Mohanavel V. (2022). Impact of multiple FSP passes on structure, mechanical, tribological and corrosion behaviors of AA6061/316 stainless-steel reinforced Al matrix composites. Surf. Coating. Technol..

[80] Sharma A., Narsimhachary D., Sharma V.M., Sahoo B., Paul J. (2019). Surface modification of Al6061-SiC surface composite through impregnation of graphene, graphite & carbon nanotubes via FSP: a tribological study. Surf. Coating. Technol..

[81] Yang R., Zhang Z., Zhao Y., Chen G., Guo Y., Liu M., Zhang J. (2015). Effect of multi-pass friction stir processing on microstructure and mechanical properties of Al3Ti/A356 composites. Mater. Char..

[82] Huang G., Shen Y., Guo R., Guan W. (2016). Fabrication of tungsten particles reinforced aluminum matrix composites using multi-pass friction stir processing: evaluation of microstructural, mechanical and electrical behavior. Mater. Sci. Eng..

[83] Dinaharan I. (2016). Influence of ceramic particulate type on microstructure and tensile strength of aluminum matrix composites produced using friction stir processing. J. Asi. Ceram. Soci..

[84] Kurt H.L. (2016). Influence of hybrid ratio and friction stir processing parameters on ultimate tensile strength of 5083 aluminum matrix hybrid composites. Compos. B Eng..

[85] Narimani M., Lotfi B., Sadeghian Z. (2016). Evaluation of the microstructure and wear behaviour of AA6063-B4C/TiB2 mono and hybrid composite layers produced by friction stir processing. Surf. Coating. Technol..

[86] Du Z., Tan M.J., Guo J.F., Bi G., Wei J. (2016). Fabrication of a new Al-Al2O3-CNTs composite using friction stir processing (FSP). Mater. Sci. Eng..

[87] Sharma D.K., Patel V., Badheka V., Mehta K., Upadhyay G. (2020). Different reinforcement strategies of hybrid surface composites AA 6061/(B4C + MoS2) produced by friction stir processing. Mater. Sci. Eng. Techn..

[88] Sharma A., Sharma V.M., Paul J. (2019). A comparative study on microstructural evolution and surface properties of graphene/CNT reinforced Al6061−SiC hybrid surface composite fabricated via friction stir processing. Trans. Nonferrous Metals Soc. China.

[89] Keshavara H., Kokabi A., Movahedi M. (2023). Microstructure and mechanical properties of Al/graphite- zirconium oxide hybrid composite fabricated by friction stir processing. Mater. Sci. Eng. A.

[90] Ravishankar B., Nayak S.K., Kader M.A. (2019). Hybrid composites for automotive applications – a review. J. Reinforc. Plast. Compos..

[91] Safri S.N.A., Sultan M.T.H., Jawaid M., Jayakrishna K. (2018). Impact behaviour of hybrid composites for structural applications: a review. Compos. B Eng..

[92] Izadi H., Gerlich A.P. (2012). Distribution and stability of carbon nanotubes during multi-pass friction stir processing of carbon nanotube/aluminum composites. Carbon.

[93] Priya A., Shrivastava A., Khatun S., Chakraborty S.S., Roy P., Kazmi K.H., Kumar P., Mukherjee S. (2022). Mechanical and electrochemical properties of friction stir processed magnesium alloy AZ31 for biomedical applications: a pilot study. Mater. Today Proc..

[94] Kundurti S.C., Sharma A., Tambe P., Kumar A. (2022). Fabrication of surface metal matrix composites for structural applications using friction stir processing – a review. Mater. Today Proc..

[95] Thankachan T., Prakash K.S., Kavimani V. (2018). Investigation on the effect of friction stir processing on Cu-BN surface composite. Mater. Manuf. Process..

[96] Thankachan T., Prakash K.S., Kavimani V. (2018). Effect of friction stir processing and hybrid reinforcement on copper. Mater. Manuf. Process..

[97] Dinaharan I., Nelsonb R., Vijay S.J., Akinlabi E.T. (2016). Microstructure and wear characterization of aluminum matrix composites reinforced with industrial waste fly ash particulates synthesized by friction stir processing. Mater. Char..

[98] Rahsepar M., Jarahimoghadam H. (2016). The influence of multipass friction stir processing on the corrosion behavior and mechanical properties of zircon-reinforced Al metal matrix composites. Mater. Sci. Eng..

[99] Khodabakhshi F., Arab S.M., Švec P., Gerlich A.P. (2017). Fabrication of a new Al-Mg/graphene nanocomposite by multi-pass friction-stir processing: dispersion, microstructure, stability, and strengthening. Mater. Char..

[100] Mehdi H., Mishra R.S. (2021). Effect of multi-pass friction stir processing and SiC nanoparticles on microstructure and mechanical properties of AA6082-T6. Adv. Indus. Manuf. Eng..

[101] Ma Z.Y., Sharma S.R., Mishra R.S. (2006). Effect of multiple-pass friction stir processing on microstructure and tensile properties of a cast aluminum–silicon alloy. Scripta Mater..

[102] Ma Z.Y., Sharma S.R., Mishra R.S. (2006). Microstructural modification of as-cast Al-Si-Mg alloy by friction stir processing. Metall. Mater. Trans..

[103] Sharma A., Sagar S., Mahto R.P., Sahoo B., Pal S.K., Paul J. (2018). Surface modification of Al6061 by graphene impregnation through a powder metallurgy assisted friction surfacing. Surf. Coating. Technol..

[104] Dadashpour M., Mostafapour A., Yeşildal R., Rouhi S. (2016). Effect of process parameter on mechanical properties and fracture behavior of AZ91C/SiO2 composite fabricated by FSP. Mater. Sci. Eng..

[105] Jamshidijam M., Fakhrabadi A.A., Masoudpanah S.M., Hasani G.H., Mangalaraja R. (2013). Wear behavior of multiwalled carbon nanotube/AZ31 composite obtained by friction stir processing. Tribol. Trans..

[106] Bhadouria N., Thakur L., Kumar P., Arora N. (2017). An investigation of normal and submerged condition on microstructural and tribological properties of friction stir processes AZ91-D magnesium alloy. Canad. J. Metall. Mater. Sci..

[107] Bates W.P., Patel V., Rana H., Anderson J., Backer J.D., Igestrand M., Fratini L. (2023). Properties augmentation of cast hypereutectic Al–Si alloy through friction stir processing. Met. Mater. Int..

[108] Arab S.M., Zebarjad S.M., Jahromi S.A.J. (2017). Fabrication of AZ31/MWCNTs surface metal matrix composites by friction stir processing: investigation of microstructure and mechanical properties. J. Mater. Eng. Perform..

[109] Rathee S., Maheshwari S., Siddiquee A.N., Srivastava M. (2017). Effect of tool plunge depth on reinforcement particles distribution in surface composite fabrication via friction stir processing. Def. Techn..

[110] Barmouz M., Givi M.K.B. (2011). Fabrication of in situ Cu/SiC composites using multi-pass friction stir processing: evaluation of microstructural, porosity, mechanical and electrical behaviour. Composites Part A Applied Science and Manufacturing.

[111] Parikh V.K., Badgujar A.D., Ghetiya N.D. (2022). Effect of friction stir processing parameters on microstructure and microhardness of aluminium based metal matrix composites. Mater. Today Proc..

[112] Sharifitabar M., Sarani A., Khorshahian S., Afarani M.S. (2011). Fabrication of 5052Al/Al2O3 nanoceramic particle reinforced composite via friction stir processing route. Mater. Des..

[113] Alidokht S., Abdollah-Zadeh A., Soleymani S., Assadi H. (2011). Microstructure and tribological performance of an aluminium alloy based hybrid composite produced by friction stir processing. Mater. Des..

[114] Khayyamin D., Mostafapour A., Keshmiri R. (2013). The effect of process parameters on microstructural characteristics of AZ91/SiO2 composite fabricated by FSP. Mater. Sci. Eng..

[115] Morisada Y., Fujii H., Nagaoka T., Fukusumi M. (2006). MWCNTs/AZ31 surface composites fabricated by friction stir processing. Mater. Sci. Eng..

[116] Asadi P., Faraji G., Masoumi A., Besharati Givi M.K. (2011). Experimental investigation of magnesium-base nano composite produced by friction stir processing: effects of particle types and number of friction stir processing passes. Metall. Mater. Trans..

[117] Azizieh M., Kokabi A.H., Abachi P. (2011). Effect of rotational speed and probe profile on microstructure and hardness of AZ31/Al2O3 nano composites fabricated by friction stir processing. Mater. Des..

[118] Faraji G., Dastani O., Akbari Mousavi S.A.A. (2011). Effect of process parameters on microstructure and micro-hardness of AZ91/Al2O3 surface composite produced by FSP. J. Mater. Eng. Perform..

[119] Bharti S., Ghetiya N.D., Patel K.M. (2021). Fabrication of AA6061/Al2O3 surface composite by double pass friction stir processing and investigation on mechanical and wear properties. Adv. Mater. Process. Techn..

[120] Ahmed M.M.Z., Refat M., El-Mahallawi I. (2014). Manufacturing of nano-surface AA 7075 composites by friction stir processing. Light Met..

[121] Sharma D.K., Patel V.V., Badheka V.J., Mehta K., Upadhyay G. (2019). Fabrication of hybrid surface composites AA 6061/(B4C + MoS2) via friction stir processing. J. Tribol..

[122] Sharma A., Sharma V.M., Mewar S., Kanta S., Paul J. (2018). Friction stir processing of Al6061-SiC-graphite hybrid surface composites. Mater. Manuf. Process..

[123] Sivanesh P.M., Elaya P.A., Arulvel S. (2020). Development of multi-pass processed AA 6082/SiCp surface composite using friction stir processing and its mechanical and tribology characterization. Surf. Coating. Technol..

[124] Kumar H., Prasad R., Kumar P. (2020). Effect of multi-groove reinforcement strategy on Cu/SiC surface composite fabricated by friction stir processing. Mater. Chem. Phys..

[125] Mishra R.S., Bieler T.R., Mukherjee A.K. (1995). Superplasticity in powder metallurgy aluminum alloys and composites. Acta Metall. Mater..

[126] Mishra R.S., Mahoney M.W. (2001). Friction stir processing: a new grain refinement technique to achieve high strain rate superplasticity in commercial alloys. Mater. Sci. Forum.

[127] Yang Y., Paidar M., Mehrez S., Ojo O.O. (2023). Enhancement of mechanical properties and wear of AA5083/316 stainless steel surface-composite developed through multi-pass friction stir processing (MPFSP). Arch. Civ. Mech. Eng..

[128] Li Shaohai, Paidar M., Liu S., Mehrez S., Kumar P.S., Mohanavel V. (2022). Importance of pin number on mechanical properties and wear performance during manufacturing of AA6061/316 surface composite via FSP. Mater. Lett..

[129] Lim D.K., Shibayanagi T., Gerlich A.P. (2009). Synthesis of multi-walled CNT reinforced aluminium alloy composite via friction stir processing. Mater. Sci. Eng. A.

[130] Sharma A., Sharma V.M., Sahoo B., Pal S.K., Paul J. (2019). Effect of multiple micro channel reinforcement filling strategy on Al6061-graphene nanocomposite fabricated through friction stir processing. J. Manuf. Process..

[131] Berbon P.B., Bingel W.H., Mishra R.S., Bampton C.C., Mahoney M.W. (2001). Friction stir processing: a tool to homogenize nanocomposite aluminum alloys. Scripta Mater..

[132] Yu C., Harald H., Banhart E., Baumeister J. (1998). Metal foaming by a powder metallurgy method: production, properties and applications. Mater. Res. Innovat..

[133] Munir K., Kingshott P., Wen C. (2015). Carbon nanotube reinforced titanium metal matrix composites prepared by powder metallurgy—a review. Crit. Rev. Solid State Mater. Sci..

[134] Zhu J., Lai Z., Yin Z., Jeon J., Lee S. (2001). Fabrication of ZrO2-NiCr functionally graded material by powder metallurgy. Mater. Chem. Phys..

[135] Nemat-Alla M.M., Ata M.H., Bayoumi M.R., Khair-Eldeen W. (2011). Powder metallurgical fabrication and microstructural investigations of Aluminium/Steel functionally graded material. Mater. Sci. Appl..

[136] Chaira D. (2021). Powder metallurgy routes for composites materials production. Encyclop. Mater.: Composites.

[137] Yankee S.J., Janowski G.M., Pletka B.J. (1990). Liquid phase sintered metal matrix composite materials. Mater. Manuf. Process..

[138] Ren Z., Zhang X.G., Sui L., Zhang T., Pang L., Jin J.Z. (2013). Fabrication of ZrB2 particles reinforced AZ31 magnesium matrix composite by powder metallurgy and subsequent hot extrusion. Mater. Res. Innovat..

[139] Bang J., Oak J.J., Park Y.H. (2016). Fabrication and analysis of the wear properties of hot-pressed Al-Si/SiCp + Al-Si-Cu-Mg metal matrix composite. J. Mater. Eng. Perform..

[140] Jeevan V., Rao C.S.P., Selvaraj N., Rao G.B. (2018). Fabrication and characterization of AA6082 - ZTA composites by powder metallurgy process. Mater. Today Proc..

[141] Verma V., Kumar B.V.M. (2017). Synthesis, microstructure and mechanical properties of Al2O3/ZrO2/CeO2 composites with addition of nickel and titania processed by conventional sintering. Mater. Today Proc..

[142] Shabani M., Paydar M.H., Moshksar M.M. (2014). Fabrication and densification enhancement of SiC-particulate-reinforced copper matrix composites prepared via the sinter-forging process. Int. J. Min. Metall. Mater..

[143] Necina V., Pabst W. (2021). Transparent MgAl2O3 spinel ceramics prepared via sinter – forging. J. Eur. Ceram. Soc..

[144] Yang H., Li Q., Wang Z., Wu H., Wu Y., Cheng X. (2022). Effect of different sintering additives on microstructure, phase compositions and mechanical properties of Si3N4/SiC ceramics. ES Mater. Manuf..

[145] Oguntuyi S.D., Shongwe M.B., Tshabalala L., Johnson O.J., Malatji N. (2022). Effects of SiC on the microstructure, densification, hardness and wear performance of TiB2 ceramic matrix composite consolidated via spark plasma sintering. Arabian J. Sci. Eng..

[146] Klein A.N., Binder R. (2015). Influence of phosphorus on the development of nickel alloy/h-BN-based self-lubricating composites processed by powder metallurgy. Compos. Interfac..

[147] Zhang Z., Liu Y., Liu H. (2022). Mechanical properties and microstructure of spark plasma sintered Al2O3-SiCw-Si3N4 composite ceramic tool materials. Ceram. Int..

[148] Kitiwan M., Katsui H., Goto T. (2021). Preparation of SiO2-diamond composites by spark plasma sintering. J. Asi. Ceram. Soci..

[149] Singh S., Gupt D., Jain V., Sharma A. (2015). Microwave processing of materials and applications in manufacturing industries: a review. Mater. Manuf. Process..

[150] Mishra R., Sharma A.K. (2016). A review of research trends in microwave processing of metal-based materials and opportunities in microwave metal casting. Crit. Rev. Solid State Mater. Sci..

[151] Wei C., Cheng J., Zhang M., Zhou R., Wei B., Yu X., Luo L., Chen P. (2022). Fabrication of diamond/WeCu functionally graded material by microwave sintering. Nucl. Eng. Technol..

[152] Ashwath P., Xavior M.A. (2018). Effect of ceramic reinforcements of microwave sintered metal matrix composites. Mater. Manuf. Process..

[153] Wang B., Zhang Z., Zhang J., Yang L., Feng D., Wu Y., Tang W. (2018). Microstructures and properties of high-fraction Sip–6061Al composites fabricated by pressureless sintering. Mater. Sci. Technol..

[154] Varol T., Canakci A., Yalcin E.D. (2017). Fabrication of nano SiC-reinforced Al 2024 matrix composites by a novel production method. Arabian J. Sci. Eng..

[155] Lin C.Y., Bathias C., Mcshane H.B., Rawlings R.D. (1999). Production of silicon carbide Al 2124 alloy functionally graded materials by mechanical powder metallurgy technique. Powder Metall..

[156] Aydin F., Sun Y. (2018). Investigation of wear behavior and microstructure of hot pressed TiB2 particulate reinforced magnesium matrix composites. Canad. J. Metall. Mater. Sci..

[157] Velez L.M., Chavez J., Hernandez L., Dominguez O. (2001). Characterization and properties of aluminium composites materials prepared by powder metallurgy techniques using ceramic solid waste. Mater. Manuf. Process..

[158] Prashanth K.G., Kumar S., Scudino S., Murty B.S., Eckert J. (2011). Fabrication and response of Al70Y16Ni10Co4 glass reinforced metal matrix composites. Mater. Manuf. Process..

[159] Elkady O.A., Abou Tabl M.A., Hamid Z.A., Moustafa S.F. (2007). Processing and evaluation of Cu/carbon fibre composites by vortex and powder metallurgy techniques. Can. Metall. Q..

[160] Li A.B., Xu H., Geng L., Li B.L., Tan Z.B., Ren W. (2012). Preparation and characterization of SiCp/2024 Al composite foams by powder metallurgy. Trans. Nonferrous Metals Soc. China.

[161] Korpe N.O., Ozkan E., Tasci U. (2017). Production of aluminium-fly ash particulate composite by powder metallurgy technique using boric acid as foaming agent. Adv. Mater. Process. Techn..

[162] Liang B., Han D., Zhang W. (2018). Fabrication and wear performance of (Cu-Sn) solution/TiCx bonded diamond composites. J. Superhard Mater..

[163] Chandrakanth R.G., Rajkumar K., Aravindan S. (2010). Fabrication of copper-TiC-graphite hybrid metal matrix composites through microwave processing. Int. J. Adv. Manuf. Technol..

[164] Yonetken A. (2015). Fabrication of electroless Ni plated Fe-Al2O3 ceramic-metal matrix composites. Trans. Indian Inst. Met..

[165] Shin H., Park H.L., Chang S.N. (2020). Fabrication, microstructures and high-strain-rate properties of TiC-reinforced titanium matrix composites. Met. Mater. Int..

[166] Chen C., Sun C., Wang W., Qi M., Han W., Li Y., Lui X., Yang F., Guo L., Guo Z. (2022). Microstructure and mechanical properties of in – situ TiB2/AlSi7Mg composites via powder metallurgy and hot extrusion. J. Mater. Res. Technol..

[167] Salur E., Aslan A., Kuntoglu M., Acarer M. (2021). Effect of ball milling time on the structural characteristics and mechanical properties of nano-sized Y2O3 particle reinforced aluminum matrix composites produced by powder metallurgy route. Adv. Powder Technol..

[168] Watanabe Y., Shibuya M., Sato H. (2013). Fabrication of Al/diamond particles functionally graded materials by centrifugal sintered-casting method. J. Phys. Conf..

[169] Watanabe Y., Sato H. (2011).

[170] Watanabe Y., Kim S.I., Fukui Y. (2005). Microstructures of functionally graded materials fabricated by centrifugal solid-particle and in-situ method. Met. Mater. Int..

[171] Fukui Y., Watanabe Y. (1996). Analysis of thermal residual stress in a thick-walled ring of duralcan base Al-SiC functionally graded. Mater. Metall. Mater. Trans. A.

[172] Adelakin T.K., Suarez O.M. (2011). Study of boride-reinforced aluminum matrix composites produced via centrifugal casting. Mater. Manuf. Process..

[173] Rajan T.P.D., Pillai R.M., Pai B.C. (2008). Centrifugal casting of functionally graded aluminium matrix composites components. Int. J. Cast Metals Res..

[174] Fukui Y. (1991). Fundamental investigation of functionally gradient material manufacturing system using centrifugal force. JSME Int. J. Ser. III.

[175] Watanabe Y., Inaguma Y., Sato H., Miura-Fujiwara E. (2009). A novel fabrication method for functionally graded materials under centrifugal force: the centrifugal mixed-powder method. Materials.

[176] Yamauchi K., Kunimine T., Sato H., Watanabe Y. (2015). Grain refinement of Al3Ti dispersed aluminum matrix composites by reaction centrifugal mixed-powder method. Mater. Trans..

[177] Miura-Fujiwara E., Sato H., Yamada M., Watanabe Y. (2012). Fabrication of metal-based functionally graded grinding wheel by a centrifugal mixed-powder method. Mater. Sci. Forum.

[178] Kunimine T., Yamada M., Sato H., Watanabe Y., Weiland H., Rollett A.D., Cassada W.A. (2012). ICAA13 Pittsburgh.

[179] Kunimine T., Shibuya M., Sato H., Watanabe Y. (2015). Fabrication of copper/diamond functionally graded materials for grinding wheels by centrifugal sintered-casting. J. Mater. Process. Technol..

[180] Matsuura K., Jinmon H., Hirashima Y., Khan T.I., Kudoh M. (2000). Reactive casting of Ni-Al-Fe ternary intermetallic alloys 200. ISIJ Int..

[181] Watanabe Y., Eryu H., Matsuura K. (2001). Evaluation of three-dimensional orientation of Al3Ti platelet in Al based FGMs fabricated by a centrifugal casting technique. Acta Mater..

[182] Ebhota W.S., Inambao F.L. (2017). Functionally graded metal matrix composite by centrifugal casting technique mathematical correlation. Afr. J. Sci. Techn. Innov. Develop..

[183] Watanabe Y., Watanabe S., Matsuura K. (2004). Nickel-Aluminides/steel clad pipe fabricated by reactive centrifugal casting method from liquid aluminum and solid nickel. Metall. Mater. Trans..

[184] Liu F., Jiang Y., Lu D., Xiao H., Tan T. (2015). Microstructure evolution and impact toughness of sandwich structured composite prepared by centrifugal casting and hot rolling process. Material Sci. Technol..

[185] Radhika N., Raghu R. (2017). The mechanical properties and abrasive wear behavior of functionally graded aluminum/AlB2 composites produced by centrifugal casting. Part. Sci. Technol..

[186] Forster M.F., Hamilton R.W., Dashwood R.J., Lee P.D. (2003). Centrifugal casting of aluminium containing in situ formed TiB2. Material Sci. Technol..

[187] Zhang J., Wang Y.Q., Zhou B.L. (1999). Microstructure and tensile properties of graded Al–Mg2 Si in situ composites fabricated by centrifugal casting. Material Sci. Technol..

[188] Samadi A., Shahbazkhani H.R. (2014). Effect of pouring temperature and casting thickness on distribution gradient of in situ formed Al2Cu particles during centrifugal casting of hypereutectic Al–Cu alloy. Int. J. Cast Metals Res..

[189] Campbell J. (2015).

[190] Bharti I., Gupta N., Gupta K.M. (2013). Novel application of functionally graded nano, optoelectronic and thermoelectric materials. Int. J. Mach. Mach. Mater..

[191] Rajan T.P.D., Pillai R.M., Pai B.C. (2007). Centrifugal casting: a potential technique for making functionally graded materials and engineering components. Indian Foundry J..

[192] Rao P., Iwasa M., Tanaka T., Kondoh I. (2003). Centrifugal casting of Al2O3–15 wt.%ZrO2 ceramic composites. Ceram. Int..

[193] Chang J.C., Velamakanni B.V., Lange F.F., Pearson D.S. (1991). Centrifugal consolidation of Al2O3 and Al2O3/ZrO2 composite slurries vs interparticle potentials: particles packing and mass segregation. J. Am. Ceram. Soc..

[194] Lange F.F. (1989). Powder processing science and technology for increased reliability. J. Am. Ceram. Soc..

[195] Jayakumar E., Jacob J.C., Rajan T.P.D., Joseph M.A., Pai B.C. (2016). Processing and characterization of functionally graded aluminum (A319)-SiCp metallic composites by centrifugal casting technique. Metall. Mater. Trans..

[196] Rajan T.P.D., Pillai R.M., Pai B.C. (2010). Characterization of centrifugal cast functionally graded aluminum-silicon carbide metal matrix composites. Mater. Char..

[197] Li B., Wang K., Liu M.X., Xue H.S., Zhu Z.Z., Liu C.M. (2013). Effects of temperature on fracture behavior of Al-based in-situ composites reinforced with Mg2Si and Si particles fabricated by centrifugal casting. Trans. Nonferrous Metals Soc. China.

[198] Wang K., Xue H.S., Zou M.H., Liu C.M. (2009). Microstructural characteristics and properties in centrifugal casting of SiCp/Zl104 composite. Trans. Nonferrous Metals Soc. China.

[199] Huang X., Liu C., Lv X., Liu G., Li F. (2011). Aluminum alloy pistons reinforced with SiC fabricated by centrifugal casting. J. Mater. Process. Technol..

[200] Zhai Y., Liu C., Wang K., Zou M., Xie Y. (2010). Characteristics of two Al based functionally gradient composites reinforced by primary Si particles and Si/in situ Mg2Si particles in centrifugal casting. Trans. Nonferrous Metals Soc. China.

[201] Radhika N., Raghu R. (2016). Development of functionally graded aluminium composites using centrifugal casting and influence of reinforcements on mechanical and wear properties. Trans. Nonferrous Metals Soc. China.

[202] Fathi R., Ma A., Saleh B., Xu Q., Jiang J. (2020). Investigation on mechanical properties and wear performance of functionally graded AZ91-SiCp composites via centrifugal casting. Mater. Today Commun..

[203] El-Galy I.M., Ahmed M.H., Bassiouny B.I. (2017). Characterization of functionally graded Al-SiCp metal matrix composites manufactured by centrifugal casting. Alex. Eng. J..

[204] Surappa M. (1997). Microstructure evolution during solidification of DRMMCs (discontinuously reinforced metal matrix composites): state of art. J. Mater. Process. Technol..

[205] Soltani S., Khosroshahi R.A., Mousavian R.T., Jiang Z.Y., Boostani A.F., Barbazon D. (2017). Stir casting process for manufacture of Al-SiC composites. Rare Met..

[206] Dasgupta R. (2012). Aluminium alloy-based metal matrix composites: a potential material for wear resistant applications. Int. Sch. Res. Notices.

[207] Singla M., Singh L., Chawla V. (2009). Study of wear properties of Al-SiC composites. J. Miner. Mater. Char. Eng..

[208] Abdizadeh H., Ebrahimifard R., Baghchesara M.A. (2014). Investigation of microstructure and mechanical properties of nano MgO reinforced Al composites manufactured by stir casting and powder metallurgy methods: a comparative study. Compos. B Eng..

[209] Ramachandra M., Radhakrishna K. (2007). Effect of reinforcement of flyash on sliding wear, slurry erosive wear and corrosive behavior of aluminium matrix composite. Wear.

[210] Sijo M.T., Jayadevan K.R. (2016). Analysis of stir cast aluminium silicon carbide metal matrix composite: a comprehensive review. Proced. Techn..

[211] Kumaran S.T., Uthayakumar M., Slota A., Aravindan S., Zajac J. (2016). Machining behavior of aa6351-SiC-B4C hybrid composites fabricated by stir casting method. Part. Sci. Technol..

[212] Hashim J., Looney L., Hashmi M. (1999). Metal matrix composites: production by the stir casting method. J. Mater. Process. Technol..

[213] Ravi B., Naik B.B., Prakash J.U. (2015). Characterization of aluminium matrix composites (AA6061/B4C) fabricated by stir casting technique. Mater. Today Proc..

[214] Yu L.I., Qiu-lin L.I., Dong L.I., Liu Wei, Guo-gang S.H.U. (2016). Fabrication and characterization of stir casting AA6061−31%B4C composite. Trans. Nonferrous Metals Soc. China.

[215] Sajjadi S., Ezatpour H., Beygi H. (2011). Microstructure and mechanical properties of Al-Al2O3 micro and nano composites fabricated by stir casting. Mater. Sci. Eng..

[216] Arunachalam R., Piya S., Krishnan P.K., Muraliraja R., Christy J.V., Mourad A.H., Maharbi M. (2020). Optimization of stir-squeeze casting parameters for production of metal matrix composites using a hybrid analytical hierarchy process–Taguchi-Grey approach. Eng. Optim..

[217] Agarwala V., Dixit D. (1981). Fabrication of aluminium base composite by foundry technique. Trans. Japan Inst. Metals.

[218] Naher S., Brabazon D., Looney L. (2003). Simulation of the stir casting process. J. Mater. Process. Technol..

[219] Rangrej S., Mehta V., Ayar V., Sutaria M. (2021). Effects of stir casting process parameters on dispersion of reinforcement particles during preparation of metal composites. Mater. Today Proc..

[220] Sharma O., Gupta P., Kumar T. (2022). Characterization of AA6063-T6/SiC/Walnut shell powder hybrid composite fabricated by electromagnetic stir-casting process with vacuum. Mater. Today Proc..

[221] Karthik R., Gopalakrishnan K., Venkatesh R., Krishnan A.M., Marimuthu S. (2022). Influence of stir casting parameters in mechanical strength analysis of Aluminium Metal Matrix Composites (AMMCs). Mater. Today Proc..

[222] Reddy G.B., Karu R., Kumar J.V., Prabunivas C.M.A., Jain R.A., Marichamy S., Ramaswamy S. (2022). Optimization of stir casting process parameter on copper aluminium composite. Mater. Today Proc..

[223] Dutta S., Narala S.K.R. (2020). Experimental investigation to study the effects of processing parameters on developed novel AM (Al-Mn) series alloy. Mater. Manuf. Process..

[224] Alagarsamy S.V., Ravichandran M., Meignanamoorthy M. (2022). Multi-objective optimisation of dry sliding wear control parameters for stir casted AA7075- TiO2 composites using Taguchi-Grey relational approach. Aust. J. Mech. Eng..

[225] Kumar A., Rana R.S., Purohit R. (2020). Effect of stirrer design on microstructure of MWCNT and Al alloy by stir casting process. Adv. Mater. Process. Techn..

[226] Chitra R., Jegan T.M.C., Bamini A.M.A., Glivin G., Frankin V.A., Vignesh R.V., Padmanaban R., Govindaraju M. (2023). Advances in Processing of Lightweight Metal Alloys and Composites. Materials Horizons: from Nature to Nanomaterials.

[227] Mollaei M., Azadi M., Tavakoli H. (2018). A parametric study on mechanical properties of aluminum-silicon/SiO2 nano-composites by a solid-liquid phase processing. Appl. Phys. A.

[228] Khademian M., Alizadeh A., Abdollahi A. (2017). Fabrication and characterization of hot rolled and hot extruded boron carbide (B4C) reinforced A356 aluminum alloy matrix composites produced by stir casting method. Trans. Indian Inst. Met..

[229] Sozhamannan G.G., Prabu S.B., Venkatagalapathy V.S.K. (2012). Effect of processing parameters on metal matrix composites: stir casting process. J. Surf. Eng. Mater. Adv. Technol..

[230] Ezatpour H.R., Sajjadi S.A., Sabzevar M.H., Huang Y. (2014). Investigation of microstructure and mechanical properties of Al6061-nanocomposite fabricated by stir casting. Mater. Des..

[231] Ghosh P., Ray S., Rohatgi P. (1984). Incorporation of alumina particles in aluminium-magnesium alloy by stirring in melt. Trans. Japan Inst. Metals.

[232] Prabu S.B., Karunamoorthy L., Kathiresan S., Mohan B. (2006). Influence of stirring speed and stirring time on distribution of particles in cast metal matrix composite. J. Mater. Process. Technol..

[233] Das S., Udhayabanu V., Das S., Das K. (2006). Synthesis and characterization of zircon sand/Al-4.5 wt% Cu composite produced by stir casting route. J. Mater. Sci..

[234] Gui M.C., Han J.M., Li P.Y. (2004). Microstructure and mechanical properties of Mg-Al9Zn/SiCp composite produced by vacuum stir casting process. Mater. Sci. Technol..

[235] Dwivedi S.P., Sharma S., Mishra R.K. (2014). Microstructure and mechanical properties of A356/SiC composites fabricated by electromagnetic stir casting. Procedia Mater. Sci..

[236] Bharath V., Ajawan S.S., Nagaral M., Auradi V., Kori S.A. (2018). Characterization and mechanical properties of 2014 aluminum alloy reinforced with Al2O3p composite produced by two-stage stir casting route. J. Inst. Eng.: Series C.

[237] Nagaral M., Hiremath V., Auradi V., Kori S.A. (2018). Influence of two-stage stir casting process on mechanical characterization and wear behavior of aa2014-ZrO2 nano-composites. Trans. Indian Inst. Met..

[238] Sekar K., Allesu K., Joseph M.A. (2015). Mechanical and wear properties of Al–Al2O3 metal matrix composites fabricated by the combined effect of stir and squeeze casting method. Trans. Indian Inst. Met..

[239] Juang S.H., Fan L.J., Yang H.P.O. (2015). Influence of preheating temperatures and adding rates on distributions of fly ash in aluminum matrix composites prepared by stir casting. Int. J. Precis. Eng. Manuf..

[240] Wang X.J., Hu X.S., Wu K., Zheng M.Y., Zheng L., Zhai Q.J. (2009). The interfacial characteristic of SiCp/AZ91 magnesium matrix composites fabricated by stir casting. J. Mater. Sci..

[241] Sekar K., Manohar M., Jayakumar K. (2018).

[242] Singh R., Podder D., Singh S. (2015). Effect of single, double and triple particle size SiC and Al2O3 reinforcement on wear properties of AMC prepared by stir casting in vacuum mould. Trans. Indian Inst. Met..

[243] Sekar K., Allesu K., Joseph M. (2013). Design, construction and performance evaluation of multiple casting machine. Niger. J. Techn..

[244] Rawal S., Das T., Sidpara A.M., Paul J. (2022). Fabrication and characterization of Al/GNPs composite by bottom pouring stir casting. Mater. Lett..

[245] Singh A., Bala N. (2017). Fabrication and tribological behavior of stir cast Mg/B4C metal matrix composites. Metall. Mater. Trans..

[246] Singh A.K., Soni S., Rana R.S. (2022). Microstructure evolution, mechanical behavior, and fracture analysis of ultrasonic-assisted stir-squeeze cast high strength AA7068/ZrO2p/Grp composite under thermal aging. Part. Sci. Technol..

[247] Pandian V., Kannan S. (2021). Processing and preparation of aerospace-grade aluminium hybrid metal matrix composite in a modified stir casting furnace integrated with mechanical supersonic vibration squeeze infiltration method. Mater. Today Commun..

[248] Singh N., Belokar R.M., Walia R.S. (2021). Experimental investigation on microstructural and mechanical attributes of Al 7075-T6/SiC/CR/MoS2 based green hybrid composite via advanced vacuum-sealed bottom pouring stir casting. Silicon.

[249] Verma A.S., Cheema M.S., Kant S., Suri N.M. (2019). Porosity study of developed Al–Mg–Si/bauxite residue metal matrix composite using advanced stir casting process. Arabian J. Sci. Eng..

[250] Suthar J., Patel K.M. (2018). Processing issues, machining, and applications of aluminum metal matrix composites. Mater. Manuf. Process..

[251] Shorowordi K.M., Laoui T., Haseeb A.S.M.A., Celis J.P., Froyen L. (2003). Microstructure and interface characteristics of B4C, SiC and Al2O3 reinforced Al matrix composites: a comparative study. J. Mater. Process. Technol..

[252] Pech-Canul M.I., Katz R.N., Makhlouf M.M. (2000). Optimum parameters for wetting silicon carbide by aluminum alloys. Metall. Mater. Trans..

[253] Pradhan S.K., Chatterjee S., Mallick A.B., Das D. (2016). A simple stir casting technique for the preparation of in-situ Fe-aluminides reinforced Al-matrix composites. Persp. Sci..

[254] Balaji V., Sateesh N., Hussain M.M. (2015). Manufacture of aluminium metal matrix composite (Al7075-SiC) by stir casting technique. Mater. Today Proc..

[255] Pozdniakov A.V., Zolotorevskiy V.S., Barkov R.Y., Lotfy A., Bazlov A.I. (2015). Microstructure and material characterization of 6063/B4C and 1545K/B4C composites produced by two stir casting techniques for nuclear applications. J. Alloys Compd..

[256] Madhusudan S., Sarcar M.M.M., Bhargava N.R.M.R. (2018). Microstrucutre and mechanical behaviour assessment of Al-Cu composites fabricated through stir casting. Part. Sci. Technol..

[257] Amouri K., Kazemi S., Momeni A., Kazazi M. (2016). Microstructure and mechanical properties of Al nano/micro SiC composites produced by stir casting technique. Mater. Sci. Eng., A.

[258] Farajollahi R., Jamshidi Aval H., Jamaati R. (2021). Effects of Ni on the microstructure, mechanical and tribological properties of AA2024-Al3NiCu composite fabricated by stir casting process. J. Alloys Compd..

[259] Lee D., Kim J., Lee S.K., Kim Y., Lee S.B., Cho S. (2020). Experimental and thermodynamic study on interfacial reaction of B4C - Al6061 composites fabricated by stir casting process. J. Alloys Compd..

[260] Gui M., Han J., Li P. (2003). Fabrication and characterization of cast magnesium matrix composites by vacuum stir casting process. J. Mater. Eng. Perform..

[261] Vinod B., Ramanathan S., Ananthi V., Selvakumar N. (2019). Fabrication and characterization of organic and in-organic reinforced A356 aluminium matrix hybrid composite by improved double-stir casting. Silicon.

[262] Hira J., Mangal S.K., Manna A. (2015). Fabrication of hybrid Mg/(Al2O3p + SiCp + Grp) metal matrix composite on developed gas injection liquid stir casting setup. Arabian J. Sci. Eng..

[263] Moses J.J., Sekhar S.J. (2016). Investigation on the tensile strength and microhardness of AA6061/TiC composites by stir casting. Trans. Indian Inst. Met..

[264] Raj R., Thakur D.G. (2016). Qualitative and quantitative assessment of microstructure in Al-B4C metal matrix composite processed by modified stir casting technique. Arch. Civ. Mech. Eng..

[265] Mohammed I., Khan A.R., Sadananda M., Shoaib S. (2016). Study of hardness and tensile strength of Aluminium-7075 percentage varying reinforced with graphite and bagasse-ash composites. Res. Eff. Techn..

[266] Dorcic JL, Verma SK. Squeeze Casting, ASM Handbook, Volume 15: Casting, ninth ed., ASM International, Materials Park, OH 198: 323-327.

[267] Qi L.H. (1998). The influence of liquid extrusion on the microstructure and properties of an Al-Si alloy. Mater. Manuf. Process..

[268] Sampath V., Ramanan N., Palaninathan R. (2006). Modeling of liquid metal infiltration of porous fiber preform during squeeze casting. Mater. Manuf. Process..

[269] Zhang Y., Wu G., Liu W., Zhang L., Pang S., Wang Y., Ding W. (2014). Effects of processing parameters and Ca content on microstructure and mechanical properties of squeeze casting AZ91–Ca alloys. Mater. Sci. Eng. A.

[270] Dao V., Zhao S., Lin W., Zhang C. (2012). Effect of process parameters on microstructure and mechanical properties in AlSi9Mg connecting-rod fabricated by semi-solid squeeze casting. Mater. Sci. Eng. A.

[271] Maeng D.Y., Lee J.H., Won C.W., Cho S.S., Chun B.S. (2000). The effects of processing parameters on the microstructure and mechanical properties of modified B390 alloy in direct squeeze casting. J. Mater. Process. Technol..

[272] Srivastava N., Anas M. (2020). An investigative review of squeeze casting: processing effects & impact on properties. Mater. Today Proc..

[273] Zhang M., Zhang W., Zhao H., Zhang D., Li Y. (2007). Effect of pressure on microstructure and mechanical properties of Al-Cu-based alloy prepared by squeeze casting. Trans. Nonferrous Metals Soc. China.

[274] Ghomashchi M.R., Vikhrov A. (2000). Squeeze casting: an overview. J. Mater. Process. Technol..

[275] Luengas L.O., Perez E.E., Munoz L., Suarez O.M. (2010). Fabrication and characterization of squeezed cast aluminum matrix composites containing boride reinforcements. J. Mater. Eng. Perform..

[276] Manu K.M.S., Resmi V.G., Brahmakumar M., Narayanasamy P., Rajan T.P.D., Pavithran C., Pai B.C. (2012).

[277] Xue J., Han Y.F., Wang J., Sun B.D. (2013). Study on squeeze casting of an in situ 5 vol.-% TiB2/2014 Al composite. Material Sci. Technol..

[278] Lo J., Shen G., Santos R. (2004). Preform cracking in squeeze cast magnesium based composites-effects of tooling temperature. Int. J. Cast Metals Res..

[279] Patel M.G.C., Krishna P., Papappagoudar M. (2015). Modelling of squeeze casting process using design of experiments and response surface methodology. Int. J. Cast Metals Res..

[280] Powell B.R., Luo A.A., Krajewski P.E. (2012).

[281] Kridli G.T., Friedman P.A., Boileau J.M. (2010).

[282] Benedyk J.C. (2010).

[283] Rosso M. (2006). Ceramic and metal matrix composites: routes and properties. J. Mater. Process. Technol..

[284] Zhang Q., Xiao B.L., Wang D., Ma Z.Y. (2011). Formation mechanism of in situ Al3Ti in Al matrix during hot pressing and subsequent friction stir processing. Mater. Chem. Phys..

[285] Hsu C.J., Chang C.Y., Kao P.W., Ho N.J., Chang C.P. (2006). Al–Al3Ti nanocomposites produced in situ by friction stir processing. Acta Mater..

[286] Birol Y. (2008). In situ synthesis of Al–TiCp composites by reacting K2TiF6 and particulate graphite in molten aluminium. J. Alloys Compd..

[287] Tong X.C., Fang H.S. (1998). Al-TiC Composites in Situ-processed by ingot metallurgy and rapid solidification technology: Part I. Microstructural evolution. Metall. Mater. Trans..

[288] Davies P., Kellie J.L.F., Patent Parton DP. (1993).

[289] Mandal A., Chakraborty M., Murty B.S. (2008). Ageing behaviour of A356 alloy reinforced with in-situ formed TiB2 particles. Mater. Sci. Eng..

[290] Sun Y., Zhao Z., Wu G. (2022). Microstrucutre characterization and mechanical properties of in situ synthesized Ti2(Al,Si)C reinforced Al composites. Mater. Char..

[291] Lakshmi S., Lu L., Gupta M. (1997). In situ preparation of TiB2 reinforced Al based composites. J. Mater. Process. Technol..

[292] Charbhai N., Murty B.S., Sankaran S. (2011).

[293] Nandam S.H., Sankaran S., Murty B.S. (2011).

[294] Zhang Y., Ma N., Wang H., Le Y., Li X. (2007). Damping capacity of in situ TiB2 particulates reinforced aluminium composites with Ti addition. Mater. Des..

[295] Rajan H.B.M., Ramabalan S., Dinaharan I., Vijay S.J. (2014). Effect of TiB2 content and temperature on sliding wear behavior of AA7075/TiB2 in situ aluminum cast composites. Arch. Civ. Mech. Eng..

[296] Wang M., Chen D., Chen Z., Wu Y., Wang F., Ma N., Wang H. (2014). Mechanical properties of in-situ TiB2/a356 composites. Mater. Sci. Eng..

[297] Bannan J., Temple R.I., Jones R. (2003). In situ fabrication of titanium carbide reinforced copper MMC. Mater. Sci. Technol..

[298] Balaji V.S., Kumaran S. (2015). Dry sliding wear behavior of titanium-(TiB+TiC) in situ composite developed by spark plasma sintering. Tribol. Trans..

[299] Khorasania F., Pourbaharia B., Emamya M., Malekana M., Salehian S. (2018). Effects of Ca/Al ratio and extrusion process on Mg–Al–Ca alloys to produce a high toughness in-situ composite. Phil. Mag..

[300] Herbert M.A., Das G., Maiti R., Chakraborty M., Mitra R. (2010). Tensile properties of cast and mushy state rolled Al-4·5Cu alloy and in situ Al-4·5Cu-5TiB2 composite. Int. J. Cast Metals Res..

[301] Liu X., Huang S., Zhang J., Hu J., Zhu H. (2022). Microstructure and mechanical properties of in-situ TiC reinforced Fe1.2MnCo0.8 medium-entropy alloy matrix composites. Mater. Today Commun..

[302] Wang Y., Tan H., Feng Z., Zhang F., Shang W., Clare A., Lin X. (2022). Enhanced mechanical properties of in situ synthesized TiC/Ti composites by pulsed laser directed energy deposition. Mater. Sci. Eng. A.

[303] Yang S., Yang Y., Chen Z. (2022). Effects of rolling method on the microstructure and anisotropy of mechanical properties of Cu–15Cr in-situ composites. Mater. Sci. Eng. A.

[304] Zhang X., Chen Z., Liu Z., He M., Yang Z., Wang J. (2022). Microstructure and enhanced mechanical properties of ZrC/Zr composites added by in-situ Y2O3 reinforced particles. Vacuum.

[305] Kumar A., Gautam R.K., Tyagi R. (2016). Dry sliding wear characteristics of in situ synthesized Al-TiC composites. Compos. Interfac..

[306] Lee K.L., Carroll H.E., Whitehouse A.F. (2000). Thermomechanical behaviour of a copper-chromium in situ composite. Mater. Sci. Technol..

[307] Madhavan S., Prabu S.B. (2013). Deformation behaviour and failure mechanisms of Al–TiB2 in situ composites. Material Sci. Technol..

[308] Chen W., Gao G., Meng X., Zhao X., Jiang Y., Wang M., Li Z., Xiao L. (2022). Microstructure, properties and strengthening mechanism of Cu-TiB2-Al2O3 composite prepared by liquid phase in-situ reaction casting. J. Alloys Compd..

[309] Zhang C., Ye F., Cheng L., Li M., Zhou J., Zhang Q. (2021). Electromagnetic wave-transparent porous silicon nitride ceramic prepared by gel-casting combined with in-situ nitridation reaction. J. Eur. Ceram. Soc..

[310] Xie Z., Jiang R., Li X., Zhang L., Li A., He Z. (2022). Microstructural evolution and mechanical properties of TiB2/2195 composites fabricated by ultrasonic-assisted in-situ casting. Ultrason. Sonochem..

[311] Lakshmanan M., Rajadurai J.S., Chakkravarthy V., Rajakarunakaran S. (2021). Tribological investigations on h-BN/NiTi inoculated Al7075 composite developed via ultrasonic aided squeeze casting. Mater. Lett..

[312] Christy J.V., Arunachalam R., Mourad A.H.I., Krishnan P.K., Piya S., Majid M. (2020). Processing, properties, and microstructure of recycled aluminum alloy composites produced through an optimized stir and squeeze casting processes. J. Manuf. Process..

[313] Arulraj M., Davim J.P., Hashmi M.S.J. (2021). Prediction of tensile strength in squeeze casted hybrid aluminium matrix composites using conventional statistical approach. Adv. Mater. Process. Techn..

[314] Asano K., Yoneda H. (2002). Microstructure and mechanical properties of AlCu-Mg alloy matrix hybrid composites fabricated by squeeze casting. Int. J. Cast Metals Res..

[315] Gurusamy P., Prabu S.B., Paskaramoorthy R. (2015). Influence of processing temperatures on mechanical properties and microstructure of squeeze cast aluminium alloy composites. Mater. Manuf. Process..

[316] Meti V.K.V., Shirur S., Nampoothiri J., Ravi K.R., Siddhalingeshwar I.G. (2018). Synthesis, characterization and mechanical properties of AA7075 based MMCs reinforced with TiB2 particles processed through ultrasound assisted in-situ casting technique. Trans. Indian Inst. Met..

